# Overexpression of FGF9 in colon cancer cells is mediated by hypoxia-induced translational activation

**DOI:** 10.1093/nar/gkt1286

**Published:** 2013-12-10

**Authors:** Tsung-Ming Chen, Yu-Heng Shih, Joseph T. Tseng, Ming-Chih Lai, Chih-Hao Wu, Yi-Han Li, Shaw-Jenq Tsai, H. Sunny Sun

**Affiliations:** ^1^Department of Physiology, College of Medicine, National Cheng Kung University, Tainan 70101, Taiwan, ^2^Institute of Basic Medical Sciences, College of Medicine, National Cheng Kung University, Tainan 70101, Taiwan, ^3^Institute of Bioinformatics and Biosignaling, College of Bioscience and Biotechnology, National Cheng Kung University, Tainan 70101, Taiwan and ^4^Institute of Molecular Medicine, College of Medicine, National Cheng Kung University, Tainan 70101, Taiwan

## Abstract

Human fibroblast growth factor 9 (FGF9) is a potent mitogen involved in many physiological processes. Although *FGF9* messenger RNA (mRNA) is ubiquitously expressed in embryos, FGF9 protein expression is generally low and restricted to a few adult organs. Aberrant expression of FGF9 usually results in human malignancies including cancers, but the mechanism remains largely unknown. Here, we report that FGF9 protein, but not mRNA, was increased in hypoxia. Two sequence elements, the upstream open reading frame (uORF) and the internal ribosome entry site (IRES), were identified in the 5' UTR of *FGF9* mRNA. Functional assays indicated that FGF9 protein synthesis was normally controlled by uORF-mediated translational repression, which kept the protein at a low level, but was upregulated in response to hypoxia through a switch to IRES-dependent translational control. Our data demonstrate that *FGF9* IRES functions as a cellular switch to turn FGF9 protein synthesis ‘on’ during hypoxia, a likely mechanism underlying FGF9 overexpression in cancer cells. Finally, we provide evidence to show that hypoxia-induced translational activation promotes FGF9 protein expression in colon cancer cells. Altogether, this dynamic working model may provide a new direction in anti-tumor therapies and cancer intervention.

## INTRODUCTION

The fibroblast growth factor (FGF) family includes at least 24 distinct polypeptides with molecular masses ranging from 17 to 34 kDa and share 13–71% sequence identity (1). Many mammalian FGFs are abundantly expressed in a specific spatial and temporal pattern and are substantially involved in many cellular processes, including development (2) and angiogenesis (3). Human fibroblast growth factor 9 (*FGF9*) (MIN# 600921) is on human chromosome 13q11–q12 and consists of three exons (4). FGF9 is a highly conserved protein with >93% sequence identity with *Xenopus*, mouse, rat and human (5,6), which suggests that FGF9 is evolutionarily important and may have similar functions across species. Previous reports demonstrated that FGF9 acts as an autocrine and/or paracrine growth factor for many different types of cells, such as neurons (6,7), uterine endometrial stroma (8,9) and fibroblasts (10). In addition, FGF9 is vital for many key processes, including development of the lungs (11) and bone (12), and for steroidogenesis in postnatal Leydig cells (13).

Although *FGF9* messenger RNA (mRNA) is ubiquitously expressed in embryos (14,15), human FGF9 protein is expressed at a relatively low level under normal physiological conditions and is restricted to a few adult organs, such as the brain, kidney (5) and uterus (8). Studies have demonstrated that FGF9 exhibits mitogenic activity in glioma (16), epithelial and fibroblast cells (17). Overexpression of FGF9 has transforming potential in NIH3T3 fibroblasts (5) and stimulates the invasion of epithelial and endothelial cells. This suggests that FGF9 overexpression might result in uncontrolled cell proliferation and malignancy. Abnormal expression of FGF9 has been associated with several human cancers (17–20) and endometriosis (21). These studies demonstrated that the expression of FGF9 must be tightly controlled to maintain its homeostasis. Our previous studies showed that FGF9 expression is rigidly regulated at multiple levels including the pre-transcriptional (22,23) and post-transcriptional levels (24,25). Nevertheless, the mechanism of how FGF9 protein is elevated in cancer cells remains largely unknown.

Compared with transcriptional regulation, the translational control of existing mRNAs permits more rapid changes in the cellular concentrations of the encoded proteins. This provides dynamic control for maintaining homeostasis in cell physiology (26). In fact, dysregulation of protein synthesis has been associated with many pathological conditions, such as cancer and several neurological disorders (27). Although cancer cells are heterogeneous, hypoxia is known to be a common stress that all cancer cells encounter. Furthermore, it is well documented that hypoxia induces translational activation in many stress-response genes. Therefore, we set out to test the hypothesis that a microenvironmental change (i.e.hypoxia) in cancer development triggers FGF9 protein expression and that the overexpressed FGF9 promotes tumor progression. Here, we report the identification of two functional elements located on the 5' UTR of *FGF9* mRNA to control FGF9 protein expression. While the upstream open reading frame (uORF) maintains FGF9 protein synthesis at a low level in normal physiological conditions, the internal ribosome entry site (IRES) increases FGF9 protein synthesis in responding to the hypoxic stress. These data support the hypothesis that overexpression of FGF9 protein in cancer cells is mediated by hypoxia-induced translational activation.

## MATERIALS AND METHODS

### Cell culture

Human embryonic kidney 293 (HEK293) cells were routinely maintained in Eagle’s minimum essential medium supplemented with 10% heat-inactivated defined horse serum, 100 U/ml penicillin, 100 μg/ml streptomycin (these three regents were obtained from Invitrogen Life Tech, Carlsbad, CA, USA) and 1.0 mM sodium pyruvate (Sigma-Aldrich, St. Louis, MO, USA) in an atmosphere of 5% CO_2_ at 37°C. For hypoxia treatment, the cells were incubated for 3, 6, 9, 12, 24 or 36 h at 37°C in a hypoxic chamber with 1% O_2_.

### Computational analysis of *FGF9* 5' UTR sequences

The sequences of *FGF9* 5' UTR from the human (D14838.1), chimpanzee (XM_001150741.1), pig (NM_213801.1), horse (XM_001489697.2), dog (XM_844845.1), mouse (NM_013518.3) and rat (NM_012952.1) species were downloaded from NCBI and aligned with a multiple sequence alignment program (http://searchlauncher.bcm.tmc.edu/multi-align/multi-align.html). Images of multiple sequence alignment were output using BOXSHADE (http://www.ch.embnet.org/software/BOX_form.html). In addition, conserved *cis*-elements of *FGF9* 5' UTR were screened using UTRdb (28), UTRscan (29) and RegRNA (http://regrna.mbc.nctu.edu.tw/) (28,30).

### Plasmid constructs

All constructs were made based on the sequences from the original submission of human *FGF9* cDNA sequence (D14838.1). The cloning vectors of pcDNA3.1 myc/His A+ (Invitrogen), pGL3-P and pRL-TK (Promega, Madison, WI, USA) were used to construct recombinant clones. In addition, pRF and phpRF vectors that contain dual-luciferase reporter genes were provided by Dr A. E. Willis at the MRC Toxicology Unit, Leicester, UK (31). The full-length *FGF9* 5'UTR (FGF9-FL. nt.1–178), predicted IRES (FGF9-IRES, nt.84–178) and deletion of IRES (FGF9-ΔIRES, nt.1–125) were polymerase chain reaction (PCR)-amplified from human genomic DNA with the proper primer set (Supplementary Table S1) and subcloned into one of three vectors: pGL3-P, pRF or phpRF. To construct uORF mutations, the two upstream AUGs on *FGF9* 5' UTR were mutated (mATGI: 1st ATG → TTG; mATGII: 2nd ATG → TTG; dmATG: 1st and 2nd ATGs → TTGs) using adapted mutagenic PCR (32). In addition, the stop codon on *FGF9* uORF was mutated (mTGA: TGA→GGA) and an additional 1-base deletion mutant (mTGA-Infr) was generated to correct the reading frame of the downstream ORF. For bicistronic plasmid construction, full-length *FGF9* 5' UTR (pRF-FL and phpR-FL; nt. 1-178), predicted IRES (pRF-IRES and phpRF-IRES; nt. 84–178) and a deletion of IRES (pR-ΔIRES and phpR-ΔIRES; nt. 1–94) were inserted between the *Renilla* (Rluc) and firefly (Fluc) luciferase coding sequences. All constructs used in this study were sequenced to confirm their authenticity before further use.

### Transient transfection and luciferase reporter assay

HEK293 cells were seeded in each well of a 24-well tissue-culture plate (2.5 × 10^5^ cells/well) (TPP AG, Trasadingen) and transfected with each plasmid construct (0.5 µg) plus plasmids carrying *Renilla* luciferase as an internal control. The transfection was carried out with Lipofectamine 2000 (Invitrogen). The cells were incubated for 24 h and harvested by adding 100 µl of reporter lysis buffer (Luciferase Assay System; Promega). The activity of firefly and *Renilla* luciferase in cell lysates was measured using a dual-luciferase reporter assay system (Promega), and TD20/20 luminometer from Turner BioSystems. β-galactosidase activity was assayed using a β-galactosidase enzyme assay system (Promega). Luciferase activity was normalized to the β-galactosidase activity. The results are presented relative to normalized luminescence driven from empty-promoter and reported as fold increases. To co-transfect small interfering RNAs (siRNAs) targeting the *Renilla* luciferase coding region, 500 ng of bicistronic reporter plasmid and 30 pmol *Renilla* luciferase siRNA (Dharmacon, Lafayette, CO, USA) were transfected using Lipofectamine 2000. All experiments were done in triplicate and independently performed at least three times.

### Ribosomal complex pull-down assay

To investigate translational activity, a ribosome complex pull-down assay was used (33). Briefly, cycloheximide (CHX) (100 µg/ml) and formaldehyde (1%) were added to HEK293 cells. Five minutes later, the cells were harvested in phosphate-buffered saline and then pelleted and suspended in lysis buffer (10 mM HEPES, 100 mM KCl, 5 mM MgCl_2_, 1% Triton-X 100, 0.5% sodium deoxycholate, 10 U/ml RNaseOUT, 100 µg/ml CHX and 1× protease inhibitor cocktail). The cells were kept on ice for 10 min, the lysates were centrifuged at 8000 rpm for 10 min and the supernatant was saved as cytoplasmic lysate. Ribosomal protein S6 antibody (2 µg) (Santa Cruz Biotechnology) was added to 200 µg of cytoplasmic lysate and incubated at 4°C overnight. Protein A/G agarose (Sigma) was added to the mixture to pull down the ribosome complex. The mRNAs bound to the ribosome complex were extracted (Qiagen) and reverse-transcribed (Applied Biosystems). cDNA was further analyzed using quantitative real-time PCR (RT-qPCR) or slot-blot analysis.

### Quantitative real-time PCR

RT-qPCR of *FGF9* and β-actin was used TaqMan assays (Applied Biosystems) in a real-time PCR system (StepOne™; Applied Biosystems). The levels of *FGF9* mRNA were measured using the 2^−ΔΔCt^ method and normalized to the expression levels of β-actin.

### Enzyme-linked immunosorbent assay

HEK293 cells were plated in equal density in six-well plates, and supernatants were collected at each time point following treatment. The concentration of total protein was determined by Bradford method, and FGF9 protein was measured by enzyme-linked immunosorbent assay (R&D Systems, Oxfordshire, UK) according to our pervious study (25).

### Slot-blot hybridization

Ribosome-associated RNA was isolated by pull-down assay and followed by RNase one (1 U/ul) digestion for 30 min at room temperature to remove the unbound RNA. cDNA made from ribosome-associated RNA was biotin-labeled using a random prime labeling kit (Pierce, Rockford, IL, USA). The probes were designed to complement the entire *FGF9* 5' UTR and partial coding sequences (Supplementary Table S1). For slot-blot hybridization, 3.2 µg of oligos was diluted with an equal volume of 0.5 M NaOH and 1.5 M NaCl and then denatured by boiling for 5–10 min. Samples were placed on ice and neutralized by adding 0.5 M Tris and 1.5 M NaCl (pH 8.0). One microgram of oligo probe was slot-blotted on a positively-charged nylon membrane (Hoefer PR 648 Slot Blot apparatus; Thermo Fisher Scientific, Singapore). Probes were fixed on a membrane using utraviolet-crosslinking (1200 J for 2 min), and the membrane was hybridized with biotin-labeled cDNA oligonucleotides for 12–16 h at 42°C, followed by signal detection (Pierce). The signal intensities were quantified using the spot density function with a gel documentation system (AlphaImager, Alpha Innotech).

### Sucrose-gradient centrifugation and polysome profiles

Before lysis, cells were pre-treated with CHX (100 µg/ml, Sigma) for 5 min and collected in phosphate-buffered saline containing 100 µg/ml CHX. All subsequent steps were performed at 4°C. Cells were lysed on ice for 10 min in RSB-150 buffer containing 10 mM Tris-HCl, pH 7.4, 3 mM MgCl_2_, 150 mM NaCl, 100 µg/ml CHX, 40 µg/ml digitonin (Calbiochem, San Diego, CA, USA), 20 U/ml RNasin (Promega) and a protease inhibitor cocktail (Thermo Scientific Inc., Bremen, Germany). After incubation on ice, cells were disrupted by passage five to six times through a 26-gauge needle. The cell lysates were collected by centrifugation in a microcentrifuge at 3000 × *g* for 2 min and clarified by further centrifugation at 11 000× *g* for 15 min. The samples were loaded on a linear gradient of 15–40% sucrose and centrifuged at 38 000rpm at 4°C for 3 h in a Beckman SW41 rotor. After centrifugation, the polysome profile was monitored at 254 nm using a fractionation system (ISCO, Lincoln, NE, USA). Sucrose gradients were split into 11 subfractions each, from 1 (top) to 11 (bottom). Total RNA was isolated using phenol–chloroform extraction followed by ethanol precipitation. The abundance of *FGF9* and β-actin mRNAs in individual fraction was measured by RT-qPCR. The translational efficiency was calculated as the ratio of polyribosome-associated *FGF9* or β-actin mRNA (fractions 7–11) to total mRNA (all fractions).

### Immunohistochemistry

Immunohistochemical staining was performed using anti-FGF9 (Sigma F1672, 1:500) and anti-hypoxia inducible factor (HIF)-1α (Novus, NB100–105, 1:250) antibodies. Procedures for immunohistochemical staining were as described previously (8). The sections were counterstained with hematoxylin. Image analysis of HIF-1α- and FGF9-positive cells was further quantified using Histoquest software (Tissue Gnostics, Vienna, Austria).

### Clinical samples

Paired normal and tumor specimens were obtained from 54 patients with colorectal cancer (CRC) who underwent surgery at National Cheng Kung University Hospital. The stage of each tumor was classified and histologically confirmed by pathologists (Supplementary Table S2). This study was approved by the Clinical Research Ethics Committee at National Cheng Kung University Medical Center, and informed consent was given by each patient.

### Statistical analysis

All experimental data were analyzed using GraphPad Prism 5.0 (GraphPad Software, San Diego, CA, USA). All data are the means ± standard error of the mean (SEM). Results were also analyzed using a *t*-test or one-way analysis of variance; the Newman–Keuls *post-hoc* test was used when appropriate. Significance was set at *P* < 0.05.

## RESULTS

### Hypoxia induces endogenous FGF9 protein synthesis

**Figure 1.**
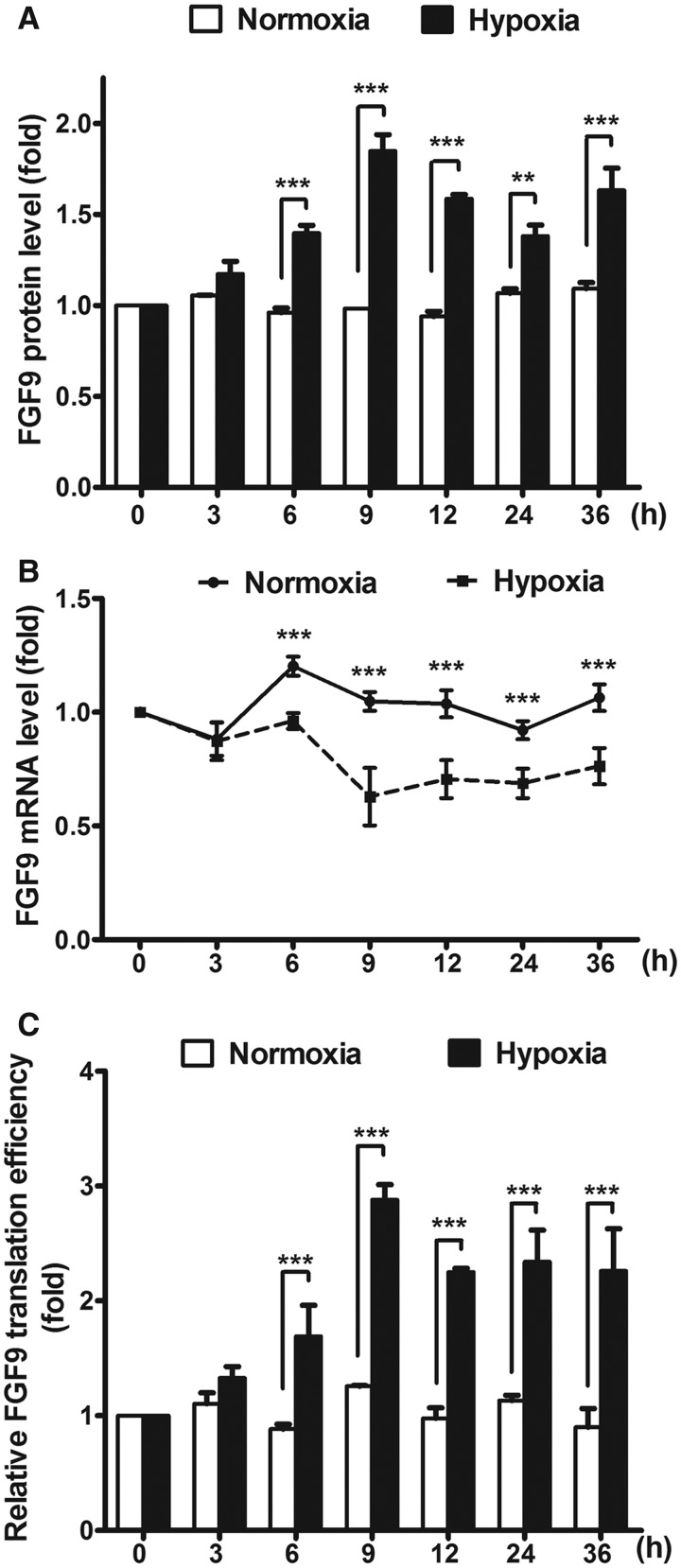
FGF9 protein expression is translationally increased in response to hypoxia. (**A** and **B**) The endogenous expression levels of *FGF9* mRNA (A) and protein (B) from HEK293 cells exposed to hypoxia or normoxia was measured at different time points and shows as relative fold-increase by normalizing to 0 h. *P < 0.05; **P < 0.01; and ***P < 0.005. (**C**) The relative translational efficiency of FGF9 was shown as ratio of FGF9 protein/mRNA. The bars represent the means ± SEM (*n* = 3). *P < 0.05; **P < 0.01****.

To study whether hypoxia can regulate FGF9 expression, we measured the endogenous mRNA and protein levels of FGF9 at different time points in HEK293 cells cultured under normoxic or hypoxic conditions. The FGF9 protein levels increased and reached a peak at 9 h after the onset of hypoxia, while the levels in normoxic cells did not change over time (Figure 1A; *P* < 0.01–0.001). Interestingly, the levels of *FGF9* mRNA decreased in hypoxic cells and were consistently lower than those in normoxic cells (Figure 1B; *P* < 0.05–0.001). To test whether the net effect of hypoxia was attributable to an increase in translational efficacy, FGF9 protein levels were normalized to the mRNA levels at the same time point. The protein-to-mRNA ratios did not change during the 36 h of normoxia (Figure 1C; *P* = 0.42), but there was a rise in FGF9 protein levels in the hypoxic cells (*P* < 0.001). The maximum ratio occurred at 9 h after hypoxic treatment and was sustained for at least an additional 25 h. This result supports the proposal that the FGF9 protein synthesis promoted by hypoxia is mediated by translational control.

### Human *FGF9* 5' UTR contains elements involved in translational regulation

**Figure 2.**
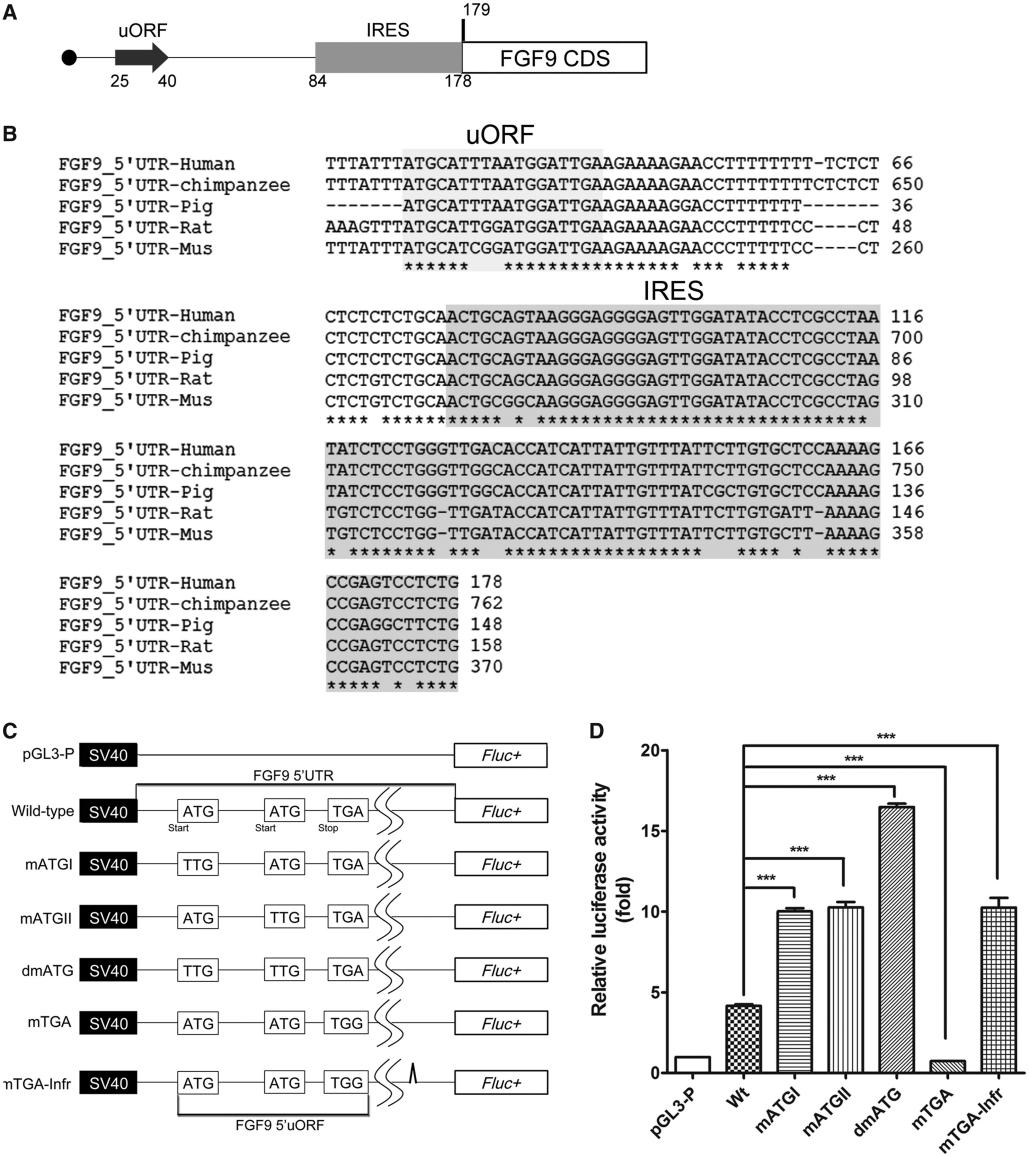
*FGF9* uORFs inhibit the expression of downstream coding sequence. (**A**) The predicted sequence elements corresponding to the uORF and IRES in the 5' UTR of *FGF9*. The ATG nucleotide sequence is the translation initiation codon. (**B**) Cross-species alignment of human, chimpanzee, pig, rat and mouse showed an overall 86% identity in the 5' UTR. (**C**) Schematic representation of pGL3-SV40-5'-UTR-Fluc reporter constructs. Six plasmids with mutations at the start (mATGI and mATGII) and stop (mTGA) codons of the uORF, double mutants of the two upstream ATG sites (dmATG) and one base deletion mutant of mTGA (mTGA-infr) were constructed. The relative positions of start and stop codons, and the base composition of each mutant plasmid are shown. (**D**) The luciferase activity of constructs shown in Figure 3C was transfected into HEK293 cell, normalized to pGL3-p and shown as means ± SEM (*n* = 3). **P < 0.01; ***P < 0.001.

To study the mechanism of increased translational efficiency under hypoxia, we first sought to identify the sequence elements in the 5' UTR region of *FGF9* that are potentially involved in translational control. A bioinformatics analysis suggested that at least two elements, the uORF with two potential initiation sites [uORFs: NCBI accession D14838.1, nucleotide (nt) 25 and nt 34] and an IRES (IRES; D14838.1, nt 84–178), were on the *FGF9* 5' UTR (Figure 2A). The predicted elements showed highly conserved features across mammalian species with an overall similarity >86%. The highly conserved sequences (Figure 2B) suggested that these two elements are functionally important in FGF9 protein synthesis.

To determine whether *FGF9* uORF functions as a regulatory element that controls FGF9 protein synthesis, a series of firefly luciferase (Fluc) reporter constructs were generated using *FGF9* 5' UTR sequences. The full-length *FGF9* 5' UTR or the uORF-mutated 5' UTR was cloned upstream of the Fluc gene. Two start codons and one stop codon were point-mutated (Figure 2C). The results showed that the luciferase activity of start codon-mutated constructs doubled compared with that in the wild-type (Figure 2D). An even stronger effect was found when both upstream AUGs were mutated (the activity tripled). In contrast, a significant decrease was found in stop codon-mutated constructs because translation from this long mRNA altered the reading frame of the downstream luciferase gene (Figure 2D). Introducing an additional base deletion to this construct corrected the reading frame and restored the luciferase activity (Figure 2D). These results indicate that the 5' short uORF of *FGF9* reduces the expression of the downstream gene, perhaps by inhibiting translational efficiency (34).

### *FGF9* IRES drives cap-independent translational initiation in cells

**Figure 3.**
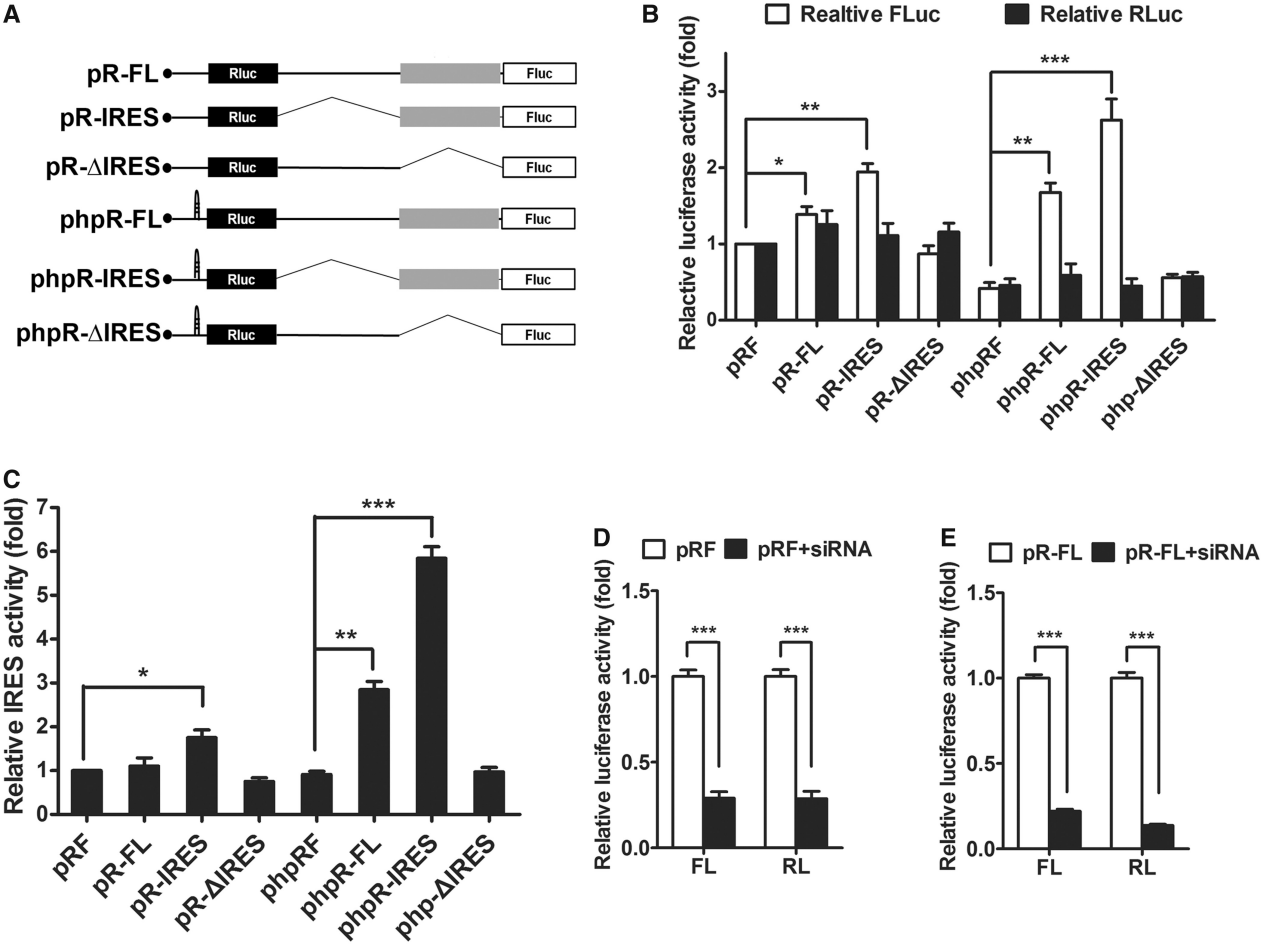
The IRES of *FGF9* regulates FGF9 protein expression through cap-independent translation. (**A**) Schematic representation of the dual-reporter constructs containing *FGF9* 5' UTR, IRES or ΔIRES. Both bicistronic systems with (phpRF) and without (pRF) an extra hairpin-loop structure in the 5' upstream of the Rluc coding sequence were used. (**B**) The relative activity of both firefly (white square) and *Renilla* (black square) luciferase was shown as fold increase by normalized to pRF. The bars represent the mean ± SEM (*n* = 3). *P < 0.05; **P < 0.01; and ***P < 0.001. (**C**) The relative IRES activity was calculated by the ratio of Fluc/Rluc and expresses as fold-increase over empty vector (pRF or phpRF). The bars represent the means ± SEM (*n* = 3). **P < 0.01; ***P < 0.001. (**D**). Repression of firefly and *Renilla* luciferase activities by cotransfection of Rluc siRNA (black square) are shown as fold-increase by normalized to pRF (white square). The bars represent the mean ± SEM (*n* = 3). ***P < 0.001. (**E**) Repression of firefly and *Renilla* luciferase activities by cotransfection of Rluc siRNA (black square) are shown as fold-increase by normalized to pRF-FL (white square). The bars represent the mean ± SEM (*n* = 3). ***P < 0.001. Proportional reduction of firefly luciferase activity and *Renilla* luciferase shows that both enzymes are derived from the same bicistronic mRNA.

To determine whether the predicted *FGF9* IRES element initiates cap-independent translation, the full-length (FL) of *FGF9* 5' UTR or the partial 5' UTR (IRES and ΔIRES) was cloned into the region between the *Renilla* luciferase (Rluc) and Fluc genes in a dual-reporter system (pRF series; Figure 3A). The activity of Rluc and Fluc represented cap-dependent and cap-independent translational efficiency, respectively. A stable RNA hairpin (ΔG = −55 kcal/mol) was introduced upstream of the Rluc gene (phpRF) to block most of the scanning ribosomes in the cap-dependent mechanism so that the relative contribution of cap-dependent and cap-independent mechanisms to FGF9 expression could be determined (phpRF series; Figure 3A). While the relative Rluc activity (i.e. cap-dependent translation) showed no difference among the pRF constructs and the phpRF constructs, an elevation of relative Fluc activity (i.e.cap-independent translation) was detected in IRES-containing pRF constructs compared with the vector controls (Figure 3B, *P* < 0.05 for pRF-FL and < 0.01 for pRF-IRES). The effect was even stronger in phpRF constructs when the hairpin structure reduced cap-dependent translation (Figure 3B, *P* < 0.01 for phpR-FL and < 0.001 for phpR-IRES). Nevertheless, the enhanced Fluc activity was abolished when there were no *FGF9* IRES sequences present in the constructs (pR-ΔIRES and phpR-ΔIRES; Figure 3B). To calculate the IRES activity, Fluc activity was normalized to Rluc activity and expressed as a fold increase over empty vector (pRF and phpRF; Figure 3C).

To test whether the presence of firefly luciferase protein in cells was translated through IRES from bicistronic constructs, we used siRNA that targeted the *Renilla* coding region to knock-down Rluc activity (35). In theory, Fluc would be reduced in proportion if it was translated from the same transcript, but unaffected if derived from a separate transcript. In cells co-transfected with the bicistronic constructs pRF (Figure 3D) or pRF-FL (Figure 3E) and *Renilla* siRNA, we found a reduction in Fluc comparable with the reduction in *Renilla* luciferase, which indicated that both enzymes had been translated from the same bicistronic mRNA. Collectively, these data indicated that the IRES of *FGF9* 5' UTR is a functional element and promotes translational efficiency *in vitro*.

### Hypoxia induces IRES-mediated translation of *FGF9* mRNA

**Figure 4.**
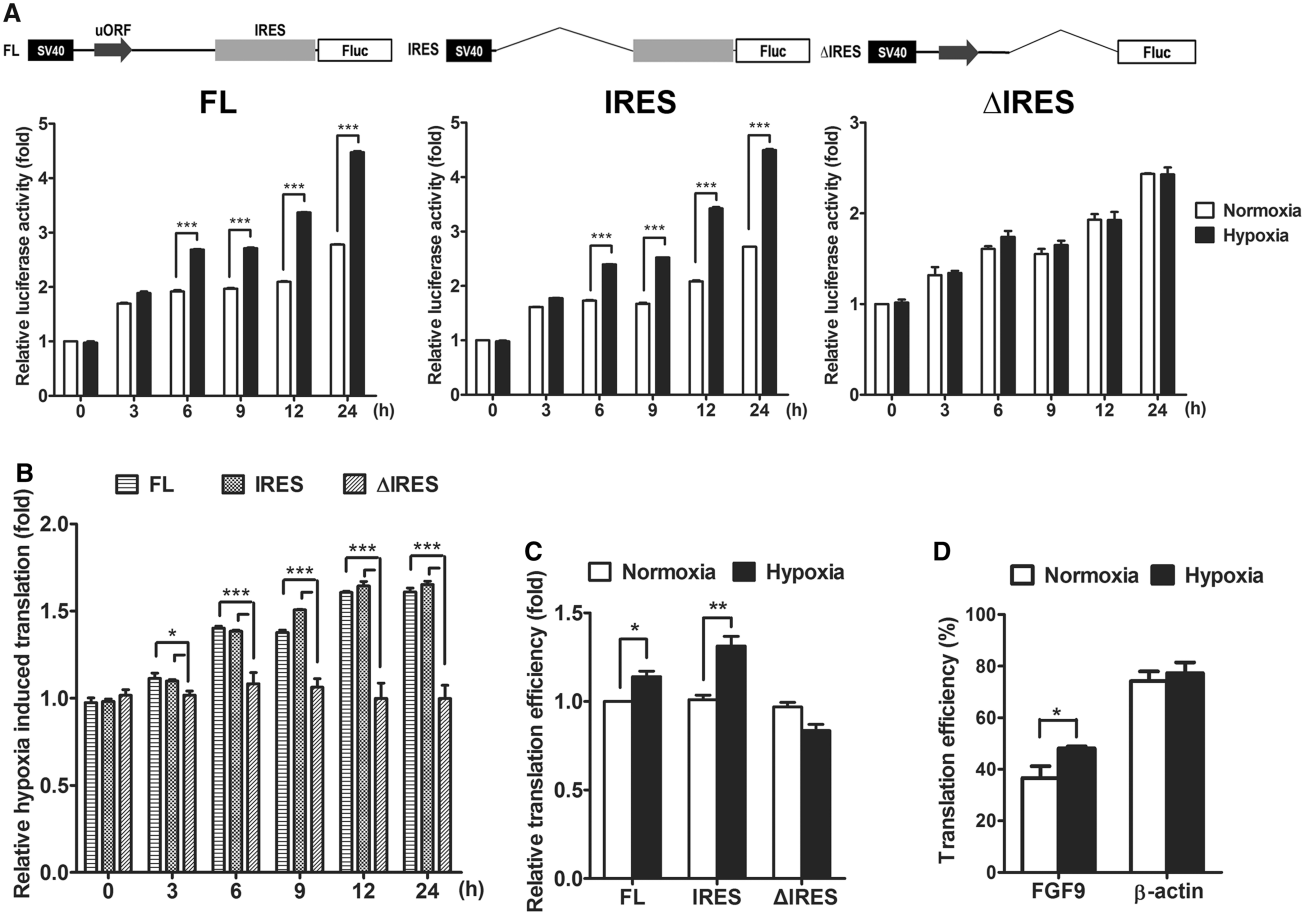
*FGF9* IRES is activated in response to hypoxia. (**A**) Hypoxia induces IRES-mediated translation of *FGF9* mRNA in HEK293 cells. Reporter constructs with full-length *FGF9* 5' UTR (FL; left), *FGF9* IRES (IRES; middle) and *FGF9* 5' UTR sequences without IRES motif (ΔIRES; right) were transfected into HEK293 cells under normoxia (white square) or hypoxia (black square), the luciferase activity was showed as relative fold increase by normalized to normoxia at 0 h. The bars represent the mean ± SEM (*n* = 3). ***P < 0.001. (**B**) The constructs shown in Figure 4A were expressed in the HEK293 cell line. The Fluc activity was measured 24 h post-transfection, and at different time points of hypoxic and normoxic exposure. The bars represent the means ± SEM (*n* = 3) as fold-increase over FL at 0 h. *P < 0.05; ***P < 0.001. (**C**) S6-IP was performed to pull down the ribosomal-mRNA complex in Fluc-constructs transfected cells. The levels of mRNA enriched by S6-IP were quantified using RT-qPCR and presented as translational efficiency. The bars represent the means ± SEM (*n* = 3) as fold-increase over FL. **P < 0.01; ***P < 0.001. (**D**) Polysome-associated transcripts of *FGF9* or β-actin are quantified using RT-qPCR. The translational efficiency of *FGF9* and β-actin exposed in hypoxia were normalized with its translational efficiency in normoxia (*n* = 3). *P < 0.05.

To determine whether elevated IRES activity increases FGF9 protein synthesis during hypoxia, we first measured the reporter activity during hypoxia using constructs with or without FGF9-IRES. After 6 h of hypoxia, reporter assays showed an increase of Fluc activity in IRES-containing constructs, but no change in IRES-deleted constructs (Figure 4A; *P* < 0.001). The luciferase activity in hypoxia was normalized to that in normoxia to represent the relative hypoxia-induced translation (Figure 4B). Interestingly, cells transfected with ΔIRES constructs, which contained the FGF9-uORF motif, showed the expression of this mRNA is not induced under hypoxia. This implied that FGF9-uORF is unaffected by hypoxia. Luciferase activity in both the full-length (Wt) and double-upstream AUG mutant (dmATG) constructs showed no detectable differences in hypoxia and normoxia (Supplementary Figure S1), which supported these results. Taken together, these data showed that hypoxia induces *FGF9* IRES activity *in vitro.*

To rule out the possibility that increased FGF9 expression during hypoxia was due to protein stability, we tested translational activity using ribosome complex pull-down with S6 antibody, followed by RT-qPCR assay (25). These procedures showed that hypoxia increased the ribosome-bound *FGF9* mRNA by 1.1-fold in FL-containing (*P* < 0.05) and 1.3-fold in IRES-containing cells (*P* < 0.01), while there was no difference of pulled-down *FGF9* mRNA between normoxia and hypoxia in cells transfected with plasmids lacking IRES (ΔIRES; Figure 4C). Next, we performed a continuous 15–40% sucrose gradient fractionation assay to measure the distribution of specific mRNAs within the polysomal fractions in normoxic and hypoxic conditions. The translational efficiency was calculated as the percentage of polysome-associated mRNA, and then the levels of specific mRNAs were detected by RT-qPCR (Supplementary Figure S2). In agreement with the results from previous sections, the polysome profiling revealed that the translational efficiency of *FGF9* mRNA increased from 36.6 to 48.1% during hypoxia (*P* = 0.03; Figure 4D, left), while the translational efficiency of β-actin mRNA did not change or even slightly decrease (Figure 4D, right). Taken together, these data confirmed that hypoxia promotes FGF9 protein expression through an increase in IRES-activated translational efficiency.

### Hypoxia-induced translational switch promotes FGF9 protein expression

**Figure 5.**
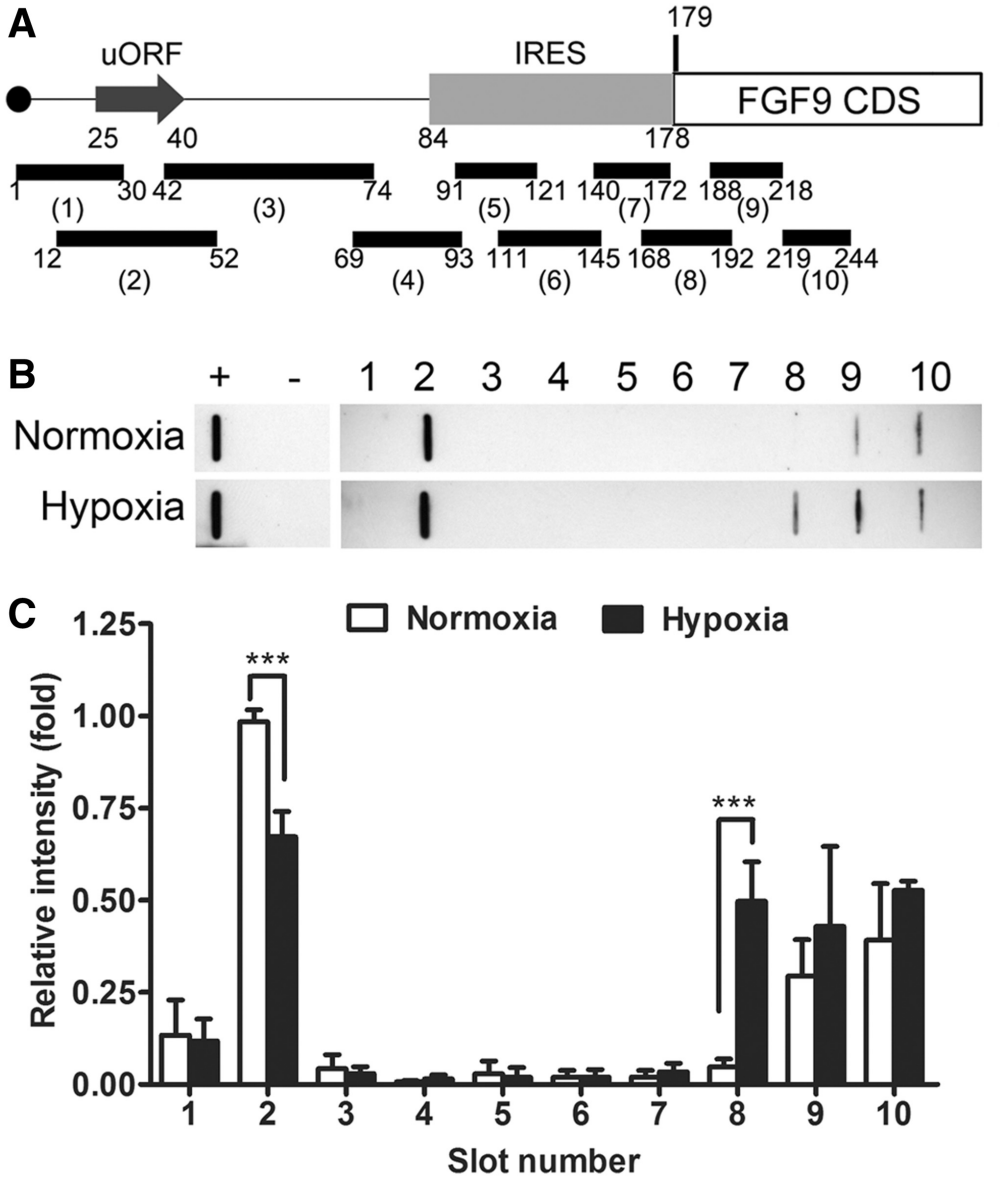
Hypoxia induces FGF9 translation. (**A**) Schematic representation of the location of probes complementary to *FGF9* 5'UTR and partial CDS, which was used in slot-plot hybridization. (**B**) The protected RNA fragments from cells under hypoxia or normoxia were purified and detected by slot-blot hybridization. The ‘+’ and ‘−’ are signals from the probes of β-actin 5' UTR and FGF9 3' UTR, respectively, which were used as the positive and negative controls in this study. (**C**) The bars represent the mean ± SEM (*n* = 4) of the spot-density quantification from Figure 5B. *P < 0.05. This suggested that the translational efficiency of FGF9 was increased in hypoxia.

To determine the mechanism underlying the translational regulation of FGF9 expression, and to study the biological significance of the two functional elements with apparently opposite effects on FGF9 translation, we modified the conventional ribosomal profiling procedure by immunoprecipitating the ribosome–mRNA complex and then digesting the unprotected region with RNase. The protected RNA fragments were then purified and detected using slot-blot hybridization. The 10 complementary probes used in hybridization were designed to cover the *FGF9* 5' UTR and a partial coding sequence (Figure 5A). The slot-blot showed that ribosomes had bound to the start codon of uORF and *FGF9* coding sequences (CDS) in both normoxic and hypoxic conditions (Figure 5B and Supplementary Figure S3). While ribosome-bound RNA significantly decreased at the position of the uORF start codon, there was a dramatic increase of pulled-down RNA at the position of the CDS start codon (slots 2 and 8, Figure 5B and C; *P* < 0.001). The weak ribosome-bound RNA signal was detected in *FGF9* CDS under normoxia, perhaps through the re-initiation mechanism (slots 9 and 10; Figure 5B, top). Notably, although it did not reach statistical significance due to the large inter-assay variation, ribosome-bound RNA levels were higher in the CDS regions after hypoxia (slots 9 and 10; Figure 5B and C). These data strongly suggest a mechanism that switches the ribosome-initiation complex from uORF to IRES during hypoxia, thus promoting translational activity and increasing FGF9 protein expression.

### FGF9 protein, but not mRNA, is overexpressed in colon cancer

**Figure 6.**
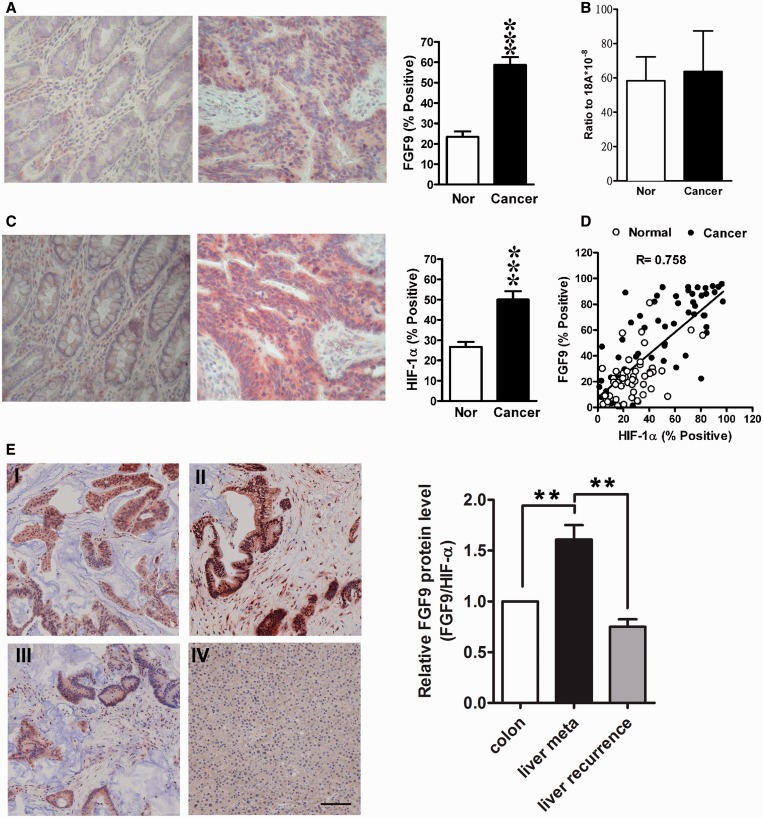
FGF9 protein is upregulated in colon cancer and is correlated with level of HIF-1α. (**A**) Left: a representative image shows the immunoreactive FGF9 protein in paired human normal colon tissue (Nor) and colon cancer (Ca). Right: percentage of FGF9-positive cells from 54 pairs of normal (Nor) and cancer samples. ***P < 0.0001. (**B**) *FGF9* mRNA was quantified by RT-qPCR in normal colon tissue (Nor) and colon cancer (*n* = 40), normalized to 18S ribosomal RNA and was expressed as ratio to 18S. Data are expressed as mean ± SEM. (**C**) Left: a representative image shows the immunoreactive HIF-1α protein in paired human normal colon tissue (Nor) and colon cancer (Ca). Right: percentage of HIF-1α positive cells from 54 pairs of normal (Nor) and cancer samples. ***P < 0.0001. (**D**) Shows the correlation between percentage of FGF9-positive and HIF-1α-positive cells in normal and cancer samples (*n* = 54). *R* = 0.758, *P* < 0.0001. (**E**) Left: a representative image shows the immunoreactive FGF9 protein in human colon cancer and liver metastasized colon cancer cell. I: primary colon cancer, II: primary colon cancer liver metastasis; III: recurrent colon cancer liver metastasis; IV: normal liver as negative control, scale bar = 100 μm. Right: the relative FGF9 protein expression (normalized by HIF-1α expression level) in cancer cells. Data were from 3 stage D CRC patients where tumor tissues from both primary liver metastasis and recurrent liver metastasis are available. Colon: primary colon cancer, liver meta: primary colon cancer liver metastasis, liver recurrence: recurrent colon cancer liver metastasis **P < 0.01.

To explore the biological significance of hypoxia-regulated FGF9 translational switch, we assessed *FGF9* mRNA and protein expression in 40 pairs of normal and cancerous colon tissues (Supplementary Table S2) by RT-qPCR and immunohistochemical staining, respectively. The levels of FGF9 protein were significantly increased in cancer cells compared with normal counterparts (Figure 6A). In contrast, the levels of *FGF9* mRNA did not differ between normal and cancerous cells (Figure 6B). To demonstrate that elevation of FGF9 protein in colon cancer cells was due to hypoxia-mediated translational upregulation, we stained HIF-1α, a marker of hypoxic cells, in the same 40 pairs of samples and found that HIF-1α was upregulated in the colon cancer cells (Figure 6C). Correlation analysis demonstrated that the levels of FGF9 were positively correlated with those of HIF-1α (Figure 6D, *R* = 0.758, *P* < 0.0001), suggesting that aberrant expression of FGF9 in colon cancer cells is induced by hypoxia. Although clinicopathological analysis revealed that levels of FGF9 protein were not associated with disease stage (data not shown), we did find FGF9 expression was significantly higher in primary liver metastasized CRC than recurrent liver metastasized CRC from the same patient (Figure 6E, *P* < 0.01). Collectively, these results demonstrated FGF9 commonly overexpressed in colon cancer cells and suggests FGF9-mediated signaling is an important mechanism in CRC tumorigenesis.

## DISCUSSION

FGF9 is a potent mitogen involved in many physiological processes. Despite previous studies by us and others showing rigid regulations at pre- (22,23) and post-transcriptional levels (24,25), overexpressed FGF9 protein is frequently observed in various cancer cells (17–20). Although aberrant expression of FGF9 is known to promote cancer progression in many cancers including colon cancer (36–38), the mechanism of how FGF9 protein is elevated in cancer cells remains largely unknown. As translational control provides cells with the plasticity and flexibility to respond to rapid changes in the environment, we hypothesized the increase of FGF9 protein resulted from the response to the microenvironmental change (i.e.hypoxia) in the cancer cells. In the present study, we found that FGF9 expression is normally controlled by uORF-mediated translational repression to keep a low level of protein synthesis; but is upregulated in response to hypoxic stress through a switch to IRES-dependent translational control. Our findings demonstrate a molecular mechanism that explains the aberrant expression of FGF9 protein, but not that of mRNA, in colon cancer cells. Nevertheless, it should be noted that the use of non-cancer cell line for *in vitro* assays in this study cannot totally exclude the involvement of other mechanisms.

Approximately 50% of all the mRNAs have been predicted to be uORF-regulated (39). In fact, 93 205' UTRs containing 20 383 uORF motifs are currently in the UTRdb (http://utrdb.ba.itb.cnr.it/). Although the uORFs are extremely diverse in both structural features and regulatory functions (40), the function of uORFs in mediating the translational repression of main protein-coding sequences by establishing barrier to scanning pre-initiation ribosomes is well known and has been validated experimentally for ∼100 eukaryotic transcripts (41). On the other hand, IRESs are known to interact with specific transactional factors to carry out the cap-independent translational initiation of protein synthesis under specific physiological conditions and stresses (e.g.apoptosis, cell-cycle, differentiation, hypoxia, oxidative stress and ER stress) (42–44). Mitchell *et al.* (45) estimated that ∼10% of mRNAs contain IRESs in their 5' UTR. Furthermore, IRESs are common in oncogenes and other genes that are involved in the control of cellular growth and differentiation (26,34,46). Taken together, these studies suggest that IRES-dependent translation is vital for regulating protein expression when cells are under stress.

A plethora of evidence shows that many uORF-containing genes with an IRES element are involved in cell growth and differentiation such as platelet-derived growth factor (47), vascular endothelial growth factor A (*VEGFA*) (48), *GATA-6* (49) and arginine/lysine transporter (*Cat-1*) (50). Furthermore, of 8231 IRES-containing 5' UTRs, more than half (4423, 53.7%) are associated with uORF motifs (Supplementary Table S3; data from the UTRdb). Although the underlying mechanism is unclear, the large number of associations points out the importance of combining two different regulatory mechanisms to control protein synthesis. Fernandez *et al.* (50) indicated that the induction of Cat-1 expression requires the translation of a small uORF within the *Cat-1* IRES to open an inhibitory ‘zipper’ structure and induce IRES activity. In addition, the uORF within an IRES in both *VEGFA* (48) and *GATA-6* (49) controls the expression of specific protein isoforms from common mRNA generated from the given gene. We suspect the distance between these two elements resided within 5' UTR may contribute to the diverse outcomes. For example, the uORF embedded in the IRES region (e.g. *Cat-1* and *VEGFA*) and the uORF located upstream of the IRES (e.g. *FGF9*) exhibit different behaviors. The regulatory mechanism of former has been illustrated where the expression of uORF is required to induce IRES-mediated translation for specific mRNA. Our study provides another example of this association. Results from our study clearly demonstrated that *FGF9* mRNA is generally translated through uORF to keep a low level of protein synthesis. Under specific environmental conditions such as hypoxia, the high level of FGF9 expression is achieved by activating FGF9-IRES, and ribosomes were switched from the ATG of uORF to ATG of the main CDS of FGF9. Thus, the data suggested a novel model that these two elements play opposite roles for FGF9 translational control to fine-tune the level of FGF9 expression in normoxia or hypoxia.

**Figure 7.**
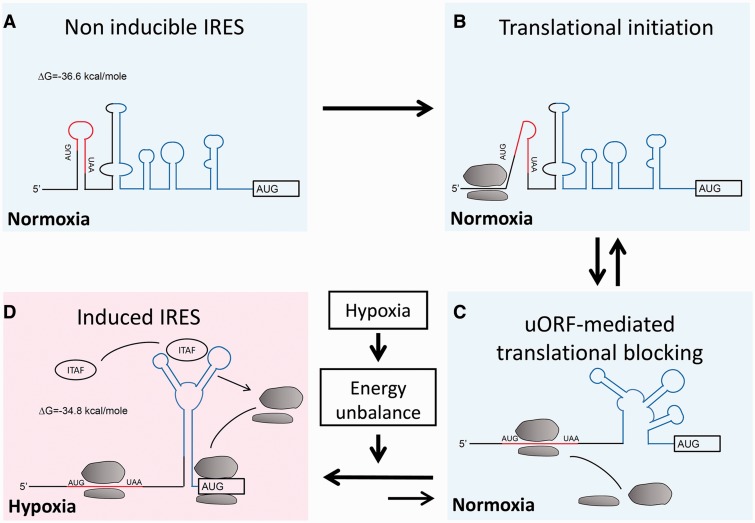
The model of IRES-mediated translational switch under hypoxia. (**A–C**)The translation-silencing conformer in normoxia allows basal FGF9 protein expression through uORF-mediated leaking translation. (**C** and **D**) Under hypoxia, energy depletion causes a conformational change of *FGF9* mRNA. A translation-permissive conformation is formed in the *FGF9* IRES structure and promotes FGF9 protein expression (D).

It is known that hypoxia-induced energy depletion affects RNA–protein interaction through both ATP-dependent (51) and ATP-independent (52) processes. A computational prediction showed that the structure of FGF9 5' UTR is dynamic (Supplementary Figure S4) and suggested a conformational change of *FGF9* IRES may be triggered by hypoxia. We propose a model of hypoxia-mediated dynamic induction to explain the mechanism that switches FGF9-IRES ‘on’ and ‘off’ for FGF9 protein translation (Figure 7). Our model suggests that FGF9-IRES functions as a cellular RNA switch that senses the low-energy signal triggered by hypoxia. The conformational change of FGF9-IRES attracts the binding of cellular IRES transacting factors (ITAF) on it, further stabilizing IRES structure, and supports efficient ribosomal recruitment to initiate IRES-mediated translation (53). A quick examination of known FGF2 ITAF, hnRNPA1 (54), showed no binding activity on FGF9-IRES (Supplementary Figure S5), the data suggested an unidentified ITAF interacts with FGF9-IRES and controls its expression under hypoxic stress. As 4000 mRNAs are predicted to have both uORF and IRES in their 5' UTR, the RNA switch may represent common machinery for controlling protein synthesis in a changing environment. Although the model sounds promising, additional studies to determine the structure of IRES-bound initiation complexes and searching for the specific ITAFs are required to establish their conformation and interactions.

Hypoxia represses classical cap-dependent translation; thus, transcripts with important physiological functions need to be translated through the initiation of IRES-dependent (44) or eukaryotic translation initiation factor 4E2 (eIF4E2)-dependent manners (55). To prevent hypoxia and apoptosis, the switch to an angiogenic phenotype is a fundamental determinant of neoplastic growth and tumor progression. Therefore, it appears that a hypoxia-mediated translation switch is an important mechanism for cancer cell survival in unfavorable conditions. Because FGF9 is a potent mitogen and survival factor, induction of its expression provides a key factor for cancer cells to survive and proliferate under unfavorable conditions like hypoxia. A similar mechanism has also been reported for the upregulation of *VEGFA* (56), *HIF1-α* (57) and *FGF2* (58). Interestingly, the overexpression of these hypoxia-induced IRES-dependent translation proteins has been linked to cancer malignancy. Taken together, these data indicate that the energy depletion caused by hypoxia selectively promotes the translational switch through IRES formation. Recently, artificial riboswitches that control translation by the presence of a specific ligand (ON) or in the absence of the ligand (OFF) in an IRES-mediated design have been reported (59). Similar types of biosensors can be used to control aberrant hypoxia-induced IRES-mediated translation during tumorigenesis. We believe it will lead to new anti-tumor treatments and strategies for future cancer intervention.

## SUPPLEMENTARY DATA

Supplementary Data are available at NAR Online.

## FUNDING

Funding for open access charge: National Science Council, Taiwan [NSC 101-2320-B-006-017 -MY2 to H.S.S.] and [NSC 101-2325-B-006-017 to S.J.T.].

*Conflict of interest statement*. None declared.

## Supplementary Material

Supplementary Data

## References

[1] Ornitz DM, Itoh N (2001). Fibroblast growth factors. Genome Biol..

[2] Goldfarb M (1996). Functions of fibroblast growth factors in vertebrate development. Cytokine Growth Factor Rev..

[3] Gerwins P, Skoldenberg E, Claesson-Welsh L (2000). Function of fibroblast growth factors and vascular endothelial growth factors and their receptors in angiogenesis. Crit. Rev. Oncol. Hematol..

[4] Mattei MG, Penault-Llorca F, Coulier F, Birnbaum D (1995). The human FGF9 gene maps to chromosomal region 13q11-q12. Genomics.

[5] Miyamoto M, Naruo K, Seko C, Matsumoto S, Kondo T, Kurokawa T (1993). Molecular cloning of a novel cytokine cDNA encoding the ninth member of the fibroblast growth factor family, which has a unique secretion property. Mol. Cell. Biol..

[6] Song J, Slack JM (1996). XFGF-9: a new fibroblast growth factor from *Xenopus* embryos. Dev. Dyn..

[7] Kanda T, Iwasaki T, Nakamura S, Kurokawa T, Ikeda K, Mizusawa H (2000). Self-secretion of fibroblast growth factor-9 supports basal forebrain cholinergic neurons in an autocrine/paracrine manner. Brain Res..

[8] Tsai SJ, Wu MH, Chen HM, Chuang PC, Wing LY (2002). Fibroblast growth factor-9 is an endometrial stromal growth factor. Endocrinology.

[9] Wing LY, Chen HM, Chuang PC, Wu MH, Tsai SJ (2005). The mammalian target of rapamycin-p70 ribosomal S6 kinase but not phosphatidylinositol 3-kinase-Akt signaling is responsible for fibroblast growth factor-9-induced cell proliferation. J. Biol. Chem..

[10] Naruo K, Seko C, Kuroshima K, Matsutani E, Sasada R, Kondo T, Kurokawa T (1993). Novel secretory heparin-binding factors from human glioma cells (glia-activating factors) involved in glial cell growth. Purification and biological properties. J. Biol. Chem..

[11] Colvin JS, Green RP, Schmahl J, Capel B, Ornitz DM (2001). Male-to-female sex reversal in mice lacking fibroblast growth factor 9. Cell.

[12] Robinson D, Hasharoni A, Evron Z, Segal M, Nevo Z (2000). Synovial chondromatosis: the possible role of FGF 9 and FGF receptor 3 in its pathology. Int. J. Exp. Pathol..

[13] Lin YM, Tsai CC, Chung CL, Chen PR, Sun HS, Tsai SJ, Huang BM (2010). Fibroblast growth factor 9 stimulates steroidogenesis in postnatal Leydig cells. Int. J. Androl..

[14] Ornitz DM, Xu J, Colvin JS, McEwen DG, MacArthur CA, Coulier F, Gao G, Goldfarb M (1996). Receptor specificity of the fibroblast growth factor family. J. Biol. Chem..

[15] Colvin JS, Feldman B, Nadeau JH, Goldfarb M, Ornitz DM (1999). Genomic organization and embryonic expression of the mouse fibroblast growth factor 9 gene. Dev. Dyn..

[16] Miyagi N, Kato S, Terasaki M, Aoki T, Sugita Y, Yamaguchi M, Shigemori M, Morimatsu M (1999). Fibroblast growth factor-9 (glia-activating factor) stimulates proliferation and production of glial fibrillary acidic protein in human gliomas either in the presence or in the absence of the endogenous growth factor expression. Oncol. Rep..

[17] Hendrix ND, Wu R, Kuick R, Schwartz DR, Fearon ER, Cho KR (2006). Fibroblast growth factor 9 has oncogenic activity and is a downstream target of Wnt signaling in ovarian endometrioid adenocarcinomas. Cancer Res..

[18] Todo T, Kondo T, Kirino T, Asai A, Adams EF, Nakamura S, Ikeda K, Kurokawa T (1998). Expression and growth stimulatory effect of fibroblast growth factor 9 in human brain tumors. Neurosurgery.

[19] Wu X, Jin C, Wang F, Yu C, McKeehan WL (2003). Stromal cell heterogeneity in fibroblast growth factor-mediated stromal-epithelial cell cross-talk in premalignant prostate tumors. Cancer Res..

[20] Schmid S, Bieber M, Zhang F, Zhang M, He B, Jablons D, Teng NN (2011). Wnt and hedgehog gene pathway expression in serous ovarian cancer. Int. J. Gynecol. Cancer.

[21] Wing LY, Chuang PC, Wu MH, Chen HM, Tsai SJ (2003). Expression and mitogenic effect of fibroblast growth factor-9 in human endometriotic implant is regulated by aberrant production of estrogen. J. Clin. Endocrinol. Metab..

[22] Chen TM, Kuo PL, Hsu CH, Tsai SJ, Chen MJ, Lin CW, Sun HS (2007). Microsatellite in the 3' untranslated region of human fibroblast growth factor 9 (FGF9) gene exhibits pleiotropic effect on modulating FGF9 protein expression. Hum. Mutat..

[23] Chuang PC, Sun HS, Chen TM, Tsai SJ (2006). Prostaglandin E2 induces fibroblast growth factor 9 via EP3-dependent protein kinase Cdelta and Elk-1 signaling. Mol. Cell. Biol..

[24] Chen TM, Hsu CH, Tsai SJ, Sun HS (2010). AUF1 p42 isoform selectively controls both steady-state and PGE2-induced FGF9 mRNA decay. Nucleic Acids Res..

[25] Gau B-HG, Chen T-M, Shih Y-HJ, Sun HS (2011). FUBP3 interacts with FGF9 3' microsatellite and positively regulates FGF9 translation. Nucleic Acids Res..

[26] Gebauer F, Hentze MW (2004). Molecular mechanisms of translational control. Nat. Rev. Mol. Cell Biol..

[27] Le Quesne JP, Spriggs KA, Bushell M, Willis AE (2010). Dysregulation of protein synthesis and disease. J. Pathol..

[28] Mignone F, Grillo G, Licciulli F, Iacono M, Liuni S, Kersey PJ, Duarte J, Saccone C, Pesole G (2005). UTRdb and UTRsite: a collection of sequences and regulatory motifs of the untranslated regions of eukaryotic mRNAs. Nucleic Acids Res..

[29] Mignone F, Gissi C, Liuni S, Pesole G (2002). Untranslated regions of mRNAs. Genome Biol..

[30] Huang HY, Chien CH, Jen KH, Huang HD (2006). RegRNA: an integrated web server for identifying regulatory RNA motifs and elements. Nucleic Acids Res..

[31] Stoneley M, Paulin FE, Le Quesne JP, Chappell SA, Willis AE (1998). C-Myc 5' untranslated region contains an internal ribosome entry segment. Oncogene.

[32] Ito W, Ishiguro H, Kurosawa Y (1991). A general method for introducing a series of mutations into cloned DNA using the polymerase chain reaction. Gene.

[33] Yeh CH, Hung LY, Hsu C, Le SY, Lee PT, Liao WL, Lin YT, Chang WC, Tseng JT (2008). RNA-binding protein HuR interacts with thrombomodulin 5'untranslated region and represses internal ribosome entry site-mediated translation under IL-1 beta treatment. Mol. Biol. Cell.

[34] Holcik M (2004). Targeting translation for treatment of cancer–a novel role for IRES?. Curr. Cancer Drug Targets.

[35] Van Eden ME, Byrd MP, Sherrill KW, Lloyd RE (2004). Demonstrating internal ribosome entry sites in eukaryotic mRNAs using stringent RNA test procedures. RNA.

[36] Teishima J, Shoji K, Hayashi T, Miyamoto K, Ohara S, Matsubara A (2012). Relationship between the localization of fibroblast growth factor 9 in prostate cancer cells and postoperative recurrence. Prostate Cancer Prostatic Dis..

[37] Yang H, Fang F, Chang R, Yang L (2013). MicroRNA-140-5p suppresses tumor growth and metastasis by targeting transforming growth factor beta receptor 1 and fibroblast growth factor 9 in hepatocellular carcinoma. Hepatology..

[38] Leushacke M, Sporle R, Bernemann C, Brouwer-Lehmitz A, Fritzmann J, Theis M, Buchholz F, Herrmann BG, Morkel M (2011). An RNA interference phenotypic screen identifies a role for FGF signals in colon cancer progression. PLoS One.

[39] Matsui M, Yachie N, Okada Y, Saito R, Tomita M (2007). Bioinformatic analysis of post-transcriptional regulation by uORF in human and mouse. FEBS Lett..

[40] Wethmar K, Smink JJ, Leutz A (2010). Upstream open reading frames: molecular switches in (patho)physiology. Bioessays.

[41] Calvo SE, Pagliarini DJ, Mootha VK (2009). Upstream open reading frames cause widespread reduction of protein expression and are polymorphic among humans. Proc. Natl Acad. Sci. USA.

[42] Spriggs KA, Stoneley M, Bushell M, Willis AE (2008). Re-programming of translation following cell stress allows IRES-mediated translation to predominate. Biol. Cell.

[43] Pearce AK, Humphrey TC (2001). Integrating stress-response and cell-cycle checkpoint pathways. Trends Cell Biol..

[44] Holcik M, Sonenberg N (2005). Translational control in stress and apoptosis. Nat. Rev. Mol. Cell Biol..

[45] Mitchell SA, Spriggs KA, Bushell M, Evans JR, Stoneley M, Le Quesne JP, Spriggs RV, Willis AE (2005). Identification of a motif that mediates polypyrimidine tract-binding protein-dependent internal ribosome entry. Genes Dev..

[46] Sonenberg N, Hinnebusch AG (2009). Regulation of translation initiation in eukaryotes: mechanisms and biological targets. Cell.

[47] Gerlitz G, Jagus R, Elroy-Stein O (2002). Phosphorylation of initiation factor-2 alpha is required for activation of internal translation initiation during cell differentiation. Eur. J. Biochem..

[48] Bastide A, Karaa Z, Bornes S, Hieblot C, Lacazette E, Prats H, Touriol C (2008). An upstream open reading frame within an IRES controls expression of a specific VEGF-A isoform. Nucleic Acids Res..

[49] Takeda M, Obayashi K, Kobayashi A, Maeda M (2004). A unique role of an amino terminal 16-residue region of long-type GATA-6. J. Biochem..

[50] Fernandez J, Yaman I, Huang C, Liu H, Lopez AB, Komar AA, Caprara MG, Merrick WC, Snider MD, Kaufman RJ (2005). Ribosome stalling regulates IRES-mediated translation in eukaryotes, a parallel to prokaryotic attenuation. Mol. Cell.

[51] Lorsch JR, Herschlag D (1998). The DEAD box protein eIF4A. 2. A cycle of nucleotide and RNA-dependent conformational changes. Biochemistry.

[52] Ray PS, Jia J, Yao P, Majumder M, Hatzoglou M, Fox PL (2009). A stress-responsive RNA switch regulates VEGFA expression. Nature.

[53] Yu Y, Abaeva IS, Marintchev A, Pestova TV, Hellen CU (2011). Common conformational changes induced in type 2 picornavirus IRESs by cognate trans-acting factors. Nucleic Acids Res..

[54] Bonnal S, Pileur F, Orsini C, Parker F, Pujol F, Prats AC, Vagner S (2005). Heterogeneous nuclear ribonucleoprotein A1 is a novel internal ribosome entry site trans-acting factor that modulates alternative initiation of translation of the fibroblast growth factor 2 mRNA. J. Biol. Chem..

[55] Uniacke J, Holterman CE, Lachance G, Franovic A, Jacob MD, Fabian MR, Payette J, Holcik M, Pause A, Lee S (2012). An oxygen-regulated switch in the protein synthesis machinery. Nature.

[56] Stein I, Itin A, Einat P, Skaliter R, Grossman Z, Keshet E (1998). Translation of vascular endothelial growth factor mRNA by internal ribosome entry: implications for translation under hypoxia. Mol. Cell. Biol..

[57] Lang KJ, Kappel A, Goodall GJ (2002). Hypoxia-inducible factor-1alpha mRNA contains an internal ribosome entry site that allows efficient translation during normoxia and hypoxia. Mol. Biol. Cell.

[58] Conte C, Riant E, Toutain C, Pujol F, Arnal JF, Lenfant F, Prats AC (2008). FGF2 translationally induced by hypoxia is involved in negative and positive feedback loops with HIF-1alpha. PLoS One.

[59] Ogawa A (2012). Rational construction of eukaryotic OFF-riboswitches that downregulate internal ribosome entry site-mediated translation in response to their ligands. Bioorg. Med. Chem. Lett..

