# Supplementary material for: Overexpression of FGF9 in colon cancer cells is mediated by hypoxia-induced translational activation
Source: Nucleic Acids Res. 2013 Dec 10;42(5):2932–44. doi: 10.1093/nar/gkt1286 (PMC3950685; doi:10.1093/nar/gkt1286)
Supplement: Supplementary Data [file supp_42_5_2932__index.html]

Overexpression of FGF9 in colon cancer cells is mediated by hypoxia-induced translational activation — Overexpression of FGF9 in colon cancer cells is mediated by hypoxia-induced translational activation — Supplementary Data 

# Overexpression of FGF9 in colon cancer cells is mediated by hypoxia-induced translational activation

## Supplementary Data

files

**Files in this Data Supplement:**

- Supplementary Data - pdf file
- Supplementary Data - doc file
