# Supplementary material for: Overexpression of FGF9 in colon cancer cells is mediated by hypoxia-induced translational activation
Source: Nucleic Acids Res. 2013 Dec 10;42(5):2932–44. doi: 10.1093/nar/gkt1286 (PMC3950685; doi:10.1093/nar/gkt1286)
Supplement: Supplementary Data [file supp_gkt1286_nar-02603-x-2013-File010.pdf]

Supplementary Table 1. Transcipts have both uORF and IRES in the 5'UTR region*

Gene Symbol	IRES Location	ID	uORF Location	IRES Size
LPXN	chr11:58099822-58099912:-	5HSAR024964	chr11:58099848-58099919:-	145
MAZ	[chr16:29725427-29725523:+	5HSAR025588	chr16:29725408-29725494:+	168
AGL	chr1:100088758-100089186:+	5HSAR029033	chr1:100088718-100088843:+<br>chr1:100088865-100088963:+	478
RTCD1	chr1:100504427-100504519:+	5HSAR015260	chr1:100504306-100504434:+	218
EXTL2	chr1:101126902-101126987:-	5HSAR029087	chr1:101132834-101132914:-	337
C1orf159	chr1:1016787-1017303:-	5HSAR012927	chr1:1041342-1041587:-	432
NTNG1	chr1:107492663-107492738:+	5HSAR026848	chr1:107492237-107492326:+<br>chr1:107492361-107492510:+	602
C1orf59	chr1:109004030-109005129:-	5HSAR013060	chr1:109004104-109005169:-	238
AMPD2	chr1:109964325-109964413:+	5HSAR018537	chr1:109964080-109964199:+	432
KCNA2	chr1:110948928-110949013:-	5HSAR011487	chr1:110949705-110949779:-<br>chr1:110949575-110949664:-	496
DENND2D	chr1:111544605-111544683:-	5HSAR017754	chr1:111544642-111544758:-	200
CTTNBP2NL	chr1:112756376-112760310:+	5HSAR019672	chr1:112740324-112756364:+	228
WNT2B	chr1:112853315-112853407:+	5HSAR013035	chr1:112853081-112853278:+	515
SLC16A1	chr1:113273454-113299469:-	5HSAR056898	chr1:113299559-113299939:-	832
DCLRE1B	chr1:114249651-114249731:+	5HSAR038161	chr1:114249641-114249715:+	294
DENND2C	chr1:114970129-114970208:-	5HSAR015942	chr1:114970176-114970247:-	333
FBXO44	chr1:11637158-11638479:+	5HSAR009730	chr1:11637982-11638224:+	237
MAD2L2	chr1:11663246-11663770:-	5HSAR011032	chr1:11663765-11663848:-	188
TRIM45	chr1:117465347-117465441:-	5HSAR022664	chr1:117465370-117465564:-	588
MAN1A2	chr1:117712248-117712328:+	5HSAR016268	chr1:117711933-117712040:+<br>chr1:117712054-117712176:+	721

UBE2J2	chr1:1188605-1193042:-	5HSAR015155	chr1:1193101-1193235:-	474
NBPF7	chr1:120188682-120188773:-	5HSAR010372	chr1:120189133-120189213:- c	621
AADACL3	chr1:12698839-12698930:+	5HSAR012340	chr1:12698705-12698785:+	226
AURKAIP1	chr1:1300000-1300280:-	5HSAR025246	chr1:1300408-1300656:-	238
PDE4DIP	chr1:143643066-143643156:-	5HSAR026341	chr1:143643114-143643182:-	324
NOTCH2NL	chr1:143920737-143960213:+	5HSAR002329	chr1:143920666-143960194:+	370
PEX11B	chr1:144227647-144227757:+	5HSAR039830	chr1:144227586-144227654:+	236
GPR89A	chr1:144538287-144538385:-	5HSAR016889	chr1:144538362-144538460:-	174
FMO5	chr1:145163246-145163553:-	5HSAR018664	chr1:145163760-145163831:-<br>chr1:145163576-145163680:-	399
BCL9	chr1:145550171-145550259:+	5HSAR018998	chr1:145479811-145479921:+<br>chr1:145479945-145480040:+	740
ACP6	chr1:145608795-145608885:-	5HSAR014911	chr1:145609023-145609106:-	464
LOC728905	chr1:145952474-145952565:-	5HSAR004081	chr1:145954004-145954114:-<br>chr1:145953667-145954002:-	1654
PLEKHO1	chr1:148388973-148389071:+	5HSAR013848	chr1:148388821-148388898:+	278
C1orf51	chr1:148522208-148522301:+	5HSAR000188	chr1:148521896-148522003:+	449
LYSMD1	chr1:149404883-149404982:-	5HSAR016042	chr1:149404899-149405009:-	166
S100A5	chr1:151780525-151780542:-	5HSAR002983	chr1:151780551-151780729:-	282
SLC39A1	chr1:152201816-152202749:-	5HSAR022098	chr1:152202753-152206797:-	466
JTB	chr1:152216353-152216444:-	5HSAR020324	chr1:152216919-152217044:-<br>chr1:152216755-152216823:-	723
ADAR	chr1:152847107-152847163:-	5HSAR030959	chr1:152841590-152841664:-<br>chr1:152841089-152841247:-	242
PMVK	chr1:153175786-153175887:-	5HSAR021915	chr1:153175870-153175959:-	323
FLAD1	chr1:153227040-153227129:+	5HSAR059857	chr1:153222398-153223096:+<br>chr1:153223153-153226623:+	893
GBA	chr1:153477528-153477615:-	5HSAR050118	chr1:153477596-153477688:-	163

RUSC1	chr1:153557404-153558188:+	5HSAR010521	chr1:153557270-153558127:+	229
C1orf104	chr1:153557904-153557997:-	5HSAR000219	chr1:153557951-153560503:-	277
SEMA4A	chr1:154390973-154390993:+	5HSAR001427	chr1:154390081-154390179:+<br>chr1:154390965-154390993:+	263
SLC25A44	chr1:154430589-154436262:+	5HSAR014358	chr1:154430363-154430503:+<br>chr1:154430539-154430607:+	322
SMG5	chr1:154519096-154519186:-	5HSAR014127	chr1:154519124-154519216:-	149
VHLL	chr1:154535605-154535705:-	5HSAR033408	chr1:154535945-154536043:-<br>chr1:154535782-154535892:-	448
FCRL5	chr1:155788777-155788879:-	5HSAR031227	chr1:155788821-155788925:-	158
FCRL1	chr1:156056452-156056549:-	5HSAR030936	chr1:156056462-156056533:-	113
AIM2	chr1:157309914-157313121:-	5HSAR001247	chr1:157313066-157313194:-	245
VANGL2	chr1:158652153-158652251:+	5HSAR009601	chr1:158637038-158652133:+	486
USP21	chr1:159396006-159397054:+	5HSAR014715	chr1:159395938-159396042:+	236
ADAMTS4	chr1:159435042-159435124:-	5HSAR030205	chr1:159435209-159435436:-	428
TOMM40L	chr1:159462817-159462903:+	5HSAR013876	chr1:159462484-159462902:+	229
FCGR3A	chr1:159786151-159786970:-	5HSAR037568	chr1:159786264-159786422:-	155
SLC35E2B	chr1:1598002-1598095:-	5HSAR021864	chr1:1598052-1613974:-	503
NOS1AP	chr1:160601677-160601763:+	5HSAR030442	chr1:160599399-160599485:+<br>chr1:160599737-160599886:+	2367
RGS4	chr1:161308480-161308818:+	5HSAR025252	chr1:161308366-161308446:+<br>chr1:161308467-161308556:+	249
RGS5	chr1:161439250-161439325:-	5HSAR038865	chr1:161439504-161439578:-<br>chr1:161439407-161439496:-	338
NUF2	chr1:161558558-161562465:+	5HSAR019188	chr1:161558352-161558480:+	300
FAM78B	chr1:164402110-164402203:-	5HSAR041262	chr1:164402364-164402507:-	473
SLC35E2	chr1:1660857-1660950:-	5HSAR000921	chr1:1660907-1667249:-	416
ADCY10	chr1:166135468-166149875:-	5HSAR040004	chr1:166149893-166150057:-	302

NME7	chr1:167603573-167603663:-	5HSAR026986	chr1:167603647-167603802:-<br>chr1:167603577-167603642:-	238
BLZF1	chr1:167604171-167605384:+	5HSAR019213	chr1:167603866-167604126:+	437
VAMP4	chr1:169974178-169977722:-	5HSAR053172	chr1:169977824-169977961:-	379
C1orf105	chr1:170656555-170656648:+	5HSAR040604	chr1:170656451-170656597:+	198
C1orf9	chr1:170769097-170769198:+	5HSAR009834	chr1:170768913-170768993:+	316
RABGAP1L	chr1:173113156-173113255:+	5HSAR020502	chr1:173111341-173111421:+	271
KIAA0040	chr1:173396773-173396862:-	5HSAR031664	chr1:173402393-173402473:-<br>chr1:173396811-173396891:-	483
RALGPS2	chr1:176961154-177012522:+	5HSAR015817	chr1:176961058-177012445:+	338
GLUL	chr1:180624496-180627005:-	5HSAR052175	chr1:180626935-180627078:-	243
RNASEL	chr1:180822565-180822648:-	5HSAR025515	chr1:180822634-180822723:-	167
C1orf14	chr1:181188892-181188990:-	5HSAR000782	chr1:181188974-181189108:-	285
C1orf26	chr1:183392987-183396596:+	5HSAR027049	chr1:183392937-183393068:+	165
CALML6	chr1:1836484-1836579:+	5HSAR002492	chr1:1836207-1836497:+	454
C1orf27	chr1:184611658-184615540:+	5HSAR032856	chr1:184611594-184615538:+	236
OCLM	chr1:184636705-184636800:+	5HSAR002520	chr1:184636363-184636437:+<br>chr1:184636443-184636538:+	474
PDC	chr1:184682238-184682325:-	5HSAR023316	chr1:184682311-184684477:-	192
FAM5C	chr1:188690644-188713243:-	5HSAR011157	chr1:188713252-188713344:-	232
ALDH4A1	chr1:19101605-19101709:-	5HSAR041273	chr1:19101784-19101852:-	276
KIAA1751	chr1:1912256-1925069:-	5HSAR010647	chr1:1925059-1925130:-	156
CAMSAP1L1	chr1:198975486-198975578:+	5HSAR000369	chr1:198975362-198975433:+<br>chr1:198975478-198975546:+	270
PPP1R12B	chr1:200584517-200584602:+	5HSAR041873	chr1:200584475-200584564:+	150
PPFIA4	chr1:201287446-201287526:+	5HSAR014690	chr1:201286997-201287110:+<br>chr1:201287169-201287390:+	593

MYOG	chr1:201321713-201321808:-	5HSAR020416	chr1:201321765-201321923:-	288
ZC3H11A	chr1:202052732-202052821:+	5HSAR002679	chr1:202032218-202037367:+ chr1:202038740-202052768:+	616
CDK18	chr1:203740478-203758918:+	5HSAR054018	chr1:203740316-203758899:+	262
SLC45A3	chr1:203900408-203900492:-	5HSAR029574	chr1:203900517-203900627:-	339
CD55	chr1:205561645-205561733:+	5HSAR030708	chr1:205561498-205561578:+	294
CD46	chr1:205992091-205992180:+	5HSAR040496	chr1:205992059-205992160:+	175
LAMB3	chr1:207890913-207891009:-	5HSAR025406	chr1:207891069-207891176:-	390
SH2D5	chr1:20927125-20931325:-	5HSAR001529	chr1:20931508-20931600:- chr1:20931305-20931496:-	502
DTL	chr1:210275770-210275855:+	5HSAR031397	chr1:210275649-210275759:+	314
TMEM206	chr1:210654723-210654831:-	5HSAR001059	chr1:210654806-210654877:-	168
FAM108A4	chr1:212849027-212849109:-	5HSAR047175	chr1:212849028-212849147:-	180
USH2A	chr1:214662302-214662396:-	5HSAR023111	chr1:214662351-214663194:-	387
ESRRG	chr1:214917444-215105100:-	5HSAR023866	chr1:215329482-215329592:- chr1:215105056-215179614:-	351
EPRS	chr1:218286354-218286438:-	5HSAR015095	chr1:218286379-218286525:-	270
BPNT1	chr1:218319812-218329729:-	5HSAR014993	chr1:218329678-218329776:-	172
DUSP10	chr1:219979710-219981994:-	5HSAR014822	chr1:219979732-219982050:-	182
FAM177B	chr1:220977908-220986510:+	5HSAR011970	chr1:220977270-220977890:+ chr1:220977904-220977984:+	266
CAPN2	chr1:221966874-221966965:+	5HSAR024200	chr1:221966753-221966836:+	224
CNIH3	chr1:222871397-222871499:+	5HSAR003812	chr1:222871115-222871387:+	698
GUK1	chr1:226395526-226399836:+	5HSAR029460	chr1:226394627-226395557:+	303
COG2	chr1:228844880-228844975:+	5HSAR024343	chr1:228844850-228844957:+	151
C1orf124	chr1:229540661-229540752:+	5HSAR013744	chr1:229540314-229540445:+	448

SIPA1L2	chr1:230717709-230717795:-	5HSAR010625	chr1:230717711-230717785:-	158
LUZP1	chr1:23293342-23293429:-	5HSAR024924	chr1:23293344-23308104:-	318
GGPS1	chr1:233558649-233565202:+	5HSAR015635	chr1:233558520-233558657:+	248
HTR1D	chr1:23393300-23393391:-	5HSAR020847	chr1:23393670-23393747:-<br>chr1:23393523-23393648:-	510
ERO1LB	chr1:234511707-234511808:-	5HSAR055498	chr1:234511715-234511912:-	256
ZNF436	chr1:23567086-23568482:-	5HSAR010079	chr1:23568755-23568868:-<br>chr1:23568582-23568707:-	559
FMN2	chr1:238321941-238322032:+	5HSAR030492	chr1:238321852-238321935:+	225
EXO1	chr1:240078875-240080350:+	5HSAR042738	chr1:240078680-240078778:+<br>chr1:240078782-240078949:+	314
PLD5	chr1:240578074-240578161:-	5HSAR001702	chr1:240754141-240754209:-	510
LOC729199	chr1:241713737-241713828:-	5HSAR003979	chr1:241714705-241714965:-<br>chr1:241714545-241714637:-	1232
PPPDE1	chr1:242883151-242883238:+	5HSAR020739	chr1:242883094-242883210:+	264
KIF26B	chr1:243385245-243385349:+	5HSAR002053	chr1:243385050-243385250:+	440
ZNF670	chr1:245308522-245308567:-	5HSAR020327	chr1:245308522-245308618:-	217
ZNF496	chr1:245559504-245559946:-	5HSAR001141	chr1:245559946-245561532:-	465
LOC100130262	chr1:247109180-247109276:-	5HSAR044454	chr1:247110239-247110307:-<br>chr1:247110083-247110223:-	1160
TNFRSF14	chr1:2486315-2486407:-	5HSAR024799	chr1:2486318-2486386:-	299
RUNX3	chr1:25128947-25129039:-	5HSAR028762	chr1:25129075-25129179:-	411
FAM54B	chr1:26020077-26022182:+	5HSAR013741	chr1:26020003-26022110:+	262
DHDDS	chr1:26631404-26632023:+	5HSAR001337	chr1:26631399-26632002:+	121
PIGV	chr1:26987307-26989893:+	5HSAR010893	chr1:26987163-26989844:+	331
WDTC1	chr1:27460053-27460132:+	5HSAR026362	chr1:27433886-27434026:+	535
PPP1R8	chr1:28040172-28040266:+	5HSAR021108	chr1:28029914-28030015:+<br>chr1:28030103-28030186:+	835

EYA3	chr1:28257125-28287647:-	5HSAR015594	chr1:28257150-28287653:-	165
SESN2	chr1:28458854-28458945:+	5HSAR001119	chr1:28458637-28458840:+	353
PHACTR4	chr1:28637386-28637482:+	5HSAR016052	chr1:28637311-28637379:+<br>chr1:28637391-28637480:+	235
MECR	chr1:29415733-29415828:-	5HSAR015628	chr1:29415866-29430005:-<br>chr1:29415765-29415851:-	391
C1orf212	chr1:35094166-35094836:-	5HSAR031840	chr1:35097121-35097204:-<br>chr1:35097042-35097107:-	395
NCDN	chr1:35796284-35796378:+	5HSAR000421	chr1:35796140-35796322:+	399
THRAP3	chr1:36497603-36520751:+	5HSAR002663	chr1:36497576-36497671:+	224
FAM176B	chr1:36561226-36562153:-	5HSAR011785	chr1:36562218-36562286:-	292
ZC3H12A	chr1:37712776-37713684:+	5HSAR012085	chr1:37712781-37713681:+	150
SF3A3	chr1:38228225-38228325:-	5HSAR029655	chr1:38228245-38228316:-	124
GJA9	chr1:39114358-39119691:-	5HSAR009863	chr1:39119736-39119846:-<br>chr1:39114393-39119726:-	281
KIAA0754	chr1:39648453-39648524:+	5HSAR000612	chr1:39647873-39647938:+<br>chr1:39647974-39648087:+	762
MYCL1	chr1:40139694-40139778:-	5HSAR013357	chr1:40140175-40140273:-	581
PPT1	chr1:40335498-40335598:-	5HSAR026369	chr1:40335508-40335594:-	232
ZMPSTE24	chr1:40496446-40496530:+	5HSAR028958	chr1:40496399-40496470:+	211
ZNF684	chr1:40769974-40771425:+	5HSAR003237	chr1:40769820-40769993:+	251
FOXJ3	chr1:42549352-42573385:-	5HSAR002369	chr1:42573403-42573468:-	191
ZMYND12	chr1:42688270-42694188:-	5HSAR026557	chr1:42694279-42694395:-	428
ERMAP	chr1:43063910-43068706:+	5HSAR014649	chr1:43055402-43063890:+<br>chr1:43063920-43063985:+	270
ATP6V0B	chr1:44213202-44213299:+	5HSAR002958	chr1:44213300-44214356:+	111
B4GALT2	chr1:44217721-44219419:+	5HSAR014844	chr1:44217589-44217654:+<br>chr1:44217685-44217756:+	370
EIF2B3	chr1:45219428-45224838:-	5HSAR054288	chr1:45224798-45224923:-	206

UROD	chr1:45250453-45250524:+	5HSAR017244	chr1:45250428-45250524:+	133
RAD54L	chr1:46486585-46486664:+	5HSAR021051	chr1:46486505-46486657:+	114
EPS15	chr1:51660217-51660318:-	5HSAR058720	chr1:51660234-51660302:-	165
NRD1	chr1:52116876-52116957:-	5HSAR016663	chr1:52117078-52117182:-	322
BTF3L4	chr1:52303112-52321605:+	5HSAR015872	chr1:52294471-52294584:+<br>chr1:52303134-52321603:+	315
LOC729225	chr1:52607221-52607294:-	5HSAR004071	chr1:52608381-52608488:-<br>chr1:52608211-52608336:-	1307
PRPF38A	chr1:52642917-52643009:+	5HSAR000394	chr1:52642810-52642953:+	203
ZYG11A	chr1:53081041-53081143:+	5HSAR027695	chr1:53080917-53081024:+	373
TMEM48	chr1:54076523-54076624:-	5HSAR042400	chr1:54076537-54076668:-	291
PCSK9	chr1:55278016-55278098:+	5HSAR040970	chr1:55277827-55277892:+	362
C1orf168	chr1:57057552-57057635:-	5HSAR001065	chr1:57057835-57057900:-<br>chr1:57057655-57057753:-	406
KCNAB2	chr1:6009069-6023215:+	5HSAR002455	chr1:6009022-6023179:+	178
NFIA	chr1:61320125-61320203:+	5HSAR023384	chr1:61320762-61320914:+	82
L1TD1	chr1:62444794-62444888:+	5HSAR034459	chr1:62434771-62441805:+	435
PGM1	chr1:63868296-63869950:+	5HSAR041672	chr1:63867709-63867780:+<br>chr1:63867808-63868281:+	681
PLEKHG5	chr1:6502159-6502243:-	5HSAR011225	chr1:6502271-6502498:-	498
LEPR	chr1:65663578-65803836:+	5HSAR038535	chr1:65658996-65803835:+	272
PDE4B	chr1:66231085-66231177:+	5HSAR030840	chr1:66231017-66231082:+	200
SGIP1	chr1:66772534-66772629:+	5HSAR025589	chr1:66772471-66772557:+	217
LOC728534	chr1:669587-669671:-	5HSAR044321	chr1:669797-669913:-<br>chr1:669628-669753:-	474
MIER1	chr1:67164430-67167174:+	5HSAR024244	chr1:67163304-67164466:+	316
IL12RB2	chr1:67546182-67558639:+	5HSAR024760	chr1:67545677-67545841:+	640

LRRIQ3	chr1:74436268-74436351:-	5HSAR011450	chr1:74436291-74436407:-	192
SLC44A5	chr1:75779721-75849342:-	5HSAR011844	chr1:75779741-75849371:-	144
PRKACB	chr1:84382541-84382632:+	5HSAR015658	chr1:84382544-84382615:+	93
BCL10	chr1:85514624-85514708:-	5HSAR040811	chr1:85514959-85515102:-	552
15-Sep	chr1:87152383-87152481:-	5HSAR014253	chr1:87152461-87152538:-	313
SLC2A5	chr1:9052183-9052260:-	5HSAR029292	chr1:9052392-9052466:- chr1:9052220-9052303:-	292
TGFBR3	chr1:92099677-92099766:-	5HSAR028537	chr1:92124143-92124304:-	466
GFI1	chr1:92721633-92724873:-	5HSAR028107	chr1:92721693-92724900:-	250
MTF2	chr1:93317610-93352852:+	5HSAR033815	chr1:93317418-93317492:+ chr1:93317549-93317644:+	317
PIK3CD	chr1:9674158-9693100:+	5HSAR025819	chr1:9674135-9693091:+	208
CLSTN1	chr1:9806379-9806469:-	5HSAR009661	chr1:9806569-9807012:-	759
ERLIN1	chr10:101935374-101935450:-	5HSAR024842	chr10:101935646-101935723:- chr10:101935408-101935518:-	351
KAZALD1	chr10:102811819-102812339:+	5HSAR022265	chr10:102811181-102811360:+	925
LDB1	chr10:103869858-103869954:-	5HSAR012571	chr10:103869860-103869928:-	343
USMG5	chr10:105142205-105144027:-	5HSAR011230	chr10:105142209-105144033:-	263
GSTO2	chr10:106024572-106024663:+	5HSAR021332	chr10:106018862-106024480:+	628
DCLRE1A	chr10:115602932-115603025:-	5HSAR020605	chr10:115603756-115603821:- chr10:115603526-115603690:-	918
NHLRC2	chr10:115604561-115604621:+	5HSAR003258	chr10:115604494-115604583:+	241
ABLIM1	chr10:116434103-116434180:-	5HSAR014207	chr10:116434171-116434392:-	302
USP6NL	chr10:11679640-11693451:-	5HSAR021739	chr10:11679677-11693572:-	321
EMX2	chr10:119292669-119292768:+	5HSAR019864	chr10:119292228-119292296:+	823
C10orf84	chr10:120085918-120091304:-	5HSAR013169	chr10:120091400-120091819:- chr10:120091256-120091384:-	267

C10orf46	chr10:120504265-120504350:-	5HSAR010695	chr10:120504292-120504720:-	484
FGFR2	chr10:123343322-123343414:-	5HSAR029255	chr10:123343324-123343401:-	150
FAM24B	chr10:124600022-124605204:-	5HSAR000393	chr10:124605235-124629142:- chr10:124600038-124605227:-	334
CHST15	chr10:125795719-125795807:-	5HSAR015401	chr10:125796085-125796168:- chr10:125795907-125796029:-	513
FAM53B	chr10:126385273-126385351:-	5HSAR001697	chr10:126385314-126385388:-	523
CTBP2	chr10:126717614-126717708:-	5HSAR028356	chr10:126717641-126789636:-	434
FAM196A	chr10:128864650-128865219:-	5HSAR018264	chr10:128884261-128884359:- chr10:128865301-128865369:-	556
OPTN	chr10:13190211-13191128:+	5HSAR023681	chr10:13182263-13190205:+	384
PHYH	chr10:13376548-13377534:-	5HSAR017203	chr10:13381567-13381635:- chr10:13376568-13377565:-	493
LRRC27	chr10:133995700-133997018:+	5HSAR018254	chr10:133995658-133996994:+	195
GPR123	chr10:134752310-134752403:+	5HSAR017022	chr10:134751542-134752248:+	436
FRMD4A	chr10:14412108-14412611:-	5HSAR003555	chr10:14412791-14412859:- chr10:14412661-14412741:-	352
DCLRE1C	chr10:15036016-15036100:-	5HSAR028904	chr10:15029611-15036015:- chr10:15024132-15024305:-	85
CACNB2	chr10:18729885-18729966:+]	5HSAR021097	chr10:18729549-18729623:+ chr10:18729646-18729726:+	448
C10orf140	chr10:21846758-21846846:-	5HSAR002548	chr10:21848522-21854503:- chr10:21848297-21848521:-	2253
ARHGAP21	chr10:25050835-25050913:-	5HSAR013530	chr10:25050841-25051059:-	487
MYO3A	chr10:26264696-26281045:+	5HSAR024421	chr10:26263010-26263090:+	360
MASTL	chr10:27484269-27484361:+	5HSAR033750	chr10:27483842-27484120:+	603
ACBD5	chr10:27569624-27569716:-	5HSAR024709	chr10:27569626-27569715:-	191
BAMBI	chr10:29006733-29006832:+	5HSAR020113	chr10:29006680-29006772:+	403
SVIL	chr10:29883994-29892179:-	5HSAR028528	chr10:29963711-29963860:- chr10:29963527-29963616:-	753
ZEB1	chr10:31650362-31650460:+	5HSAR027457	chr10:31650089-31650241:+ chr10:31650345-31650431:+	391

EPC1	chr10:32675850-32675947:-	5HSAR019117	chr10:32676049-32676117:-	270
CCDC7	chr10:32775534-32780576:+	5HSAR023824	chr10:32775457-32775549:+	570
CREM	chr10:35524920-35524996:+	5HSAR029696	chr10:35524841-35524909:+	161
BMS1	chr10:42598223-42599848:+	5HSAR024656	chr10:42598173-42598259:+	358
ZNF239	chr10:43373534-43373623:-	5HSAR010221	chr10:43373545-43373616:-	171
PPYR1	chr10:46505662-46506789:+	5HSAR024975	chr10:46503701-46505622:+<br>chr10:46505624-46505692:+	419
LOC338598	chr10:48708129-48708216:+	5HSAR045031	chr10:48707588-48707668:+<br>chr10:48707672-48707740:+	655
SLC18A3	chr10:50488707-50488792:+	5HSAR026329	chr10:50488434-50488559:+	440
CHAT	chr10:50492133-50492241:+	5HSAR023721	chr10:50492091-50492192:+	153
OGDHL	chr10:50630001-50630243:-	5HSAR025019	chr10:50630711-50630779:-	393
SGMS1	chr10:51773881-51773959:-	5HSAR022965	chr10:52053633-52053731:-<br>chr10:51949684-52020001:-	954
A1CF	chr10:52289707-52315348:-	5HSAR001633	chr10:52293843-52315435:-	188
LOC100132159	chr10:5548235-5548325:-	5HSAR006680	chr10:5548403-5548480:-	319
FAM13C	chr10:60782111-60782189:-	5HSAR048518	chr10:60792192-60792297:-<br>chr10:60785668-60790824:-	468
SLC16A9	chr10:61114056-61139297:-]	5HSAR013112	chr10:61139413-61139493:-	449
RBM17	chr10:6171865-6179034:+	5HSAR023264	chr10:6171435-6171536:+<br>chr10:6171661-6171774:+	644
ANK3	chr10:61819303-61819387:-	5HSAR021048	chr10:61819324-61819392:-	192
RHOBTB1	chr10:62341307-62373765:-	5HSAR016577	chr10:62373805-62373897:-	366
TMEM26	chr10:62882846-62882939:-	5HSAR013114	chr10:62883044-62883181:-	369
C10orf107	chr10:63092925-63111006:+	5HSAR013118	chr10:63092737-63110914:+	305
CTNNA3	chr10:69077278-69095397:-	5HSAR027024	chr10:69095340-69095420:-	123
SIRT1	chr10:69321166-69321261:+	5HSAR031143	chr10:69317199-69317297:+	432

MYPN	chr10:69539394-69551201:+	5HSAR013750	chr10:69539261-69539374:+	232
ATOH7	chr10:69661441-69661528:-	5HSAR012794	chr10:69661551-69661706:-	421
HK1	chr10:70725420-70730596:+	5HSAR028438	chr10:70699816-70712310:+	499
TYSND1	chr10:71576349-71576449:-	5HSAR009618	chr10:71576411-71576491:-	154
LRRC20	chr10:71806296-71811281:-	5HSAR003463	chr10:71811333-71811410:-	222
KIAA1274	chr10:71908773-71955713:+	5HSAR027259	chr10:71908677-71955698:+	281
PCBD1	chr10:72318297-72318392:-	5HSAR014866	chr10:72318401-72318505:-	251
P4HA1	chr10:74504648-74507547:-	5HSAR020322	chr10:74504672-74507575:-	334
PLAU	chr10:75341561-75341655:+	5HSAR029763	chr10:75341320-75341397:+<br>chr10:75341507-75341611:+	482
AP3M1	chr10:75568144-75580605:-	5HSAR025926	chr10:75580724-75580831:-	317
MYST4	chr10:76272547-76272621:+	5HSAR017683	chr10:76256411-76272370:+<br>chr10:76272385-76272462:+	493
VDAC2	chr10:76641262-76641347:+	5HSAR042687	chr10:76640923-76641292:+	555
C10orf11	chr10:77212650-77212739:+	5HSAR001970	chr10:77212599-77212739:+	215
KCNMA1	chr10:79067407-79067484:-	5HSAR027911	chr10:79067409-79067489:-	177
POLR3A	chr10:79459172-79459272:-	5HSAR050094	chr10:79459216-79459284:-	133
C10orf58	chr10:82158288-82170203:+	5HSAR020036	chr10:82158229-82158330:+	160
NRG3	chr10:83627602-83627696:+	5HSAR048941	chr10:83627461-83627592:+<br>chr10:83627600-83627680:+	274
TUBB8	chr10:84616-84785:-	5HSAR032516	chr10:84840-85502:-	316
LIPJ	chr10:90340332-90340424:+	5HSAR002284	chr10:90336506-90336574:+<br>chr10:90336580-90337074:+	314
LIPM	chr10:90552536-90552633:+	5HSAR011414	chr10:90552546-90552617:+	167
FAS	chr10:90740524-90740613:+	5HSAR030213	chr10:90740300-90740437:+	346
SLC16A12	chr10:91212316-91284269:-	5HSAR052094	chr10:91212316-91284296:-	301

PANK1	chr10:91393462-91393556:-	5HSAR021092	chr10:91393504-91393587:-	166
IDE	chr10:94233066-94237008:-	5HSAR038041	chr10:94237034-94240276:- chr10:94236968-94237033:-	325
ENTPD1	chr10:97573029-97594223:+	5HSAR051228	chr10:97505713-97505805:+	469
C10orf131	chr10:97661052-97663172:+	5HSAR024058	chr10:97657750-97663155:+	196
CCNJ	chr10:97793399-97794140:+	5HSAR012947	chr10:97793149-97793331:+	359
ZNF518A	chr10:97905971-97906069:+	5HSAR013779	chr10:97879646-97882364:+ chr10:97883303-97901210:+	857
OPALIN	chr10:98103203-98108678:-	5HSAR013908	chr10:98108830-98109081:- chr10:98108708-98108803:-	508
C10orf62	chr10:99339572-99339678:+	5HSAR031946	chr10:99339533-99339664:+	205
C10orf28	chr10:99913083-99957861:+	5HSAR000338	chr10:99912645-99913108:+	295
ANGPTL5	chr11:101283851-101291967:-	5HSAR011030	chr11:101292340-101292462:- chr11:101292046-101292126:-	596
AMPD3	chr11:10433480-10439642:+	5HSAR017052	chr11:10433316-10433396:+ chr11:10433404-10433487:+	335
ATM	chr11:107599061-107603561:+	5HSAR031187	chr11:107598826-107599005:+	385
ALG9	chr11:111236532-111241167:-	5HSAR018037	chr11:111246858-111247049:- chr11:111246726-111246842:-	889
DLAT	chr11:111401303-111401406:+	5HSAR027611	chr11:111400819-111400941:+	659
C11orf57	chr11:111450760-111451508:+	5HSAR010455	chr11:111450295-111450387:+	681
TAGLN	chr11:116575674-116578939:+	5HSAR031501	chr11:116575499-116575582:+	518
FXYD6	chr11:117218702-117234142:-	5HSAR051177	chr11:117252641-117252937:-	618
SCN4B	chr11:117519891-117519975:-	5HSAR024065	chr11:117521178-117521339:- chr11:117519980-117521082:-	509
AMICA1	chr11:117590792-117600947:-	5HSAR018779	chr11:117589174-117589263:- chr11:117589057-117589122:-	174
MPZL2	chr11:117640079-117640181:-	5HSAR057742	chr11:117640206-117640325:-	383
ATP5L	chr11:117777495-117777590:+	5HSAR013758	chr11:117777333-117777416:+	277
DDX6	chr11:118162171-118162263:-	5HSAR025875	chr11:118162433-118167149:-	433

BCL9L	chr11:118285859-118285962:-	5HSAR003516	chr11:118286190-118286396:-	965
SC5DL	chr11:120668651-120679294:+	5HSAR019358	chr11:120668618-120668686:+	148
EI24	chr11:124944660-124947633:+	5HSAR003485	chr11:124944577-124944645:+	242
CHEK1	chr11:125001051-125001873:+	5HSAR029053	chr11:125000303-125000419:+	892
CDON	chr11:125398582-125438359:-	5HSAR001309	chr11:125398603-125438383:-	128
FLI1	chr11:128067893-128133300:+	5HSAR042140	chr11:128067665-128067751:+	388
KCNJ1	chr11:128215349-128242362:-	5HSAR023966	chr11:128242396-128242467:-	222
ARHGAP32	chr11:128373530-128399012:-	5HSAR016841	chr11:128399126-128399290:- chr11:128399022-128399087:-	379
PRDM10	chr11:129336070-129336163:-	5HSAR002371	chr11:129336105-129336185:-	232
NTM	chr11:131286498-131286585:+	5HSAR018675	chr11:131286124-131286237:+	664
IGSF9B	chr11:133331860-133331943:-	5HSAR000598	chr11:133331890-133332009:-	231
GLB1L2	chr11:133707070-133707165:+	5HSAR036101	chr11:133707039-133707155:+	188
B3GAT1	chr11:133762764-133762998:-	5HSAR030039	chr11:133762937-133786949:-	261
SPON1	chr11:13940925-13941027:+	5HSAR021606	chr11:13940621-13940695:+	538
PDE3B	chr11:14622110-14622197:+	5HSAR018370	chr11:14622012-14622083:+	353
KCNJ11	chr11:17366215-17366297:-	5HSAR030093	chr11:17366491-17366565:-	568
MRGPRX3	chr11:18110792-18115325:+	5HSAR011682	chr11:18099209-18110756:+	361
LDHA	chr11:18374589-18374670:+	5HSAR050978	chr11:18374485-18374634:+	282
E2F8	chr11:19218245-19218340:-	5HSAR016037	chr11:19218695-19218883:-	522
NAV2	chr11:19328979-19329075:+	5HSAR014060	chr11:19328851-19328940:+	229
SIRT3	chr11:223390-223475:-	5HSAR027117	chr11:223529-226328:-	314
GAS2	chr11:22646404-22652991:+	5HSAR018348	chr11:22646354-22646443:+	248

PSMD13	chr11:226974-227049:+	5HSAR011123	chr11:226820-227008:+	242
KCNQ1	chr11:2439418-2439515:+	5HSAR029820	chr11:2439269-2439340:+<br>chr11:2439342-2439497:+	256
BDNF	chr11:27636688-27678344:-	5HSAR030532	chr11:27678496-27678600:-<br>chr11:27678328-27678423:-	346
ZNF195	chr11:3356847-3356952:-	5HSAR012140	chr11:3356874-3357014:-	178
CD59	chr11:33700568-33714533:-	5HSAR030641	chr11:33709584-33714562:-	161
LMO2	chr11:33847839-33847929:-	5HSAR031217	chr11:33870154-33870363:-<br>chr11:33869957-33870058:-	830
ELF5	chr11:34489693-34489784:-	5HSAR009412	chr11:34489749-34489856:-	230
PDHX	chr11:34894677-34894778:+	5HSAR013452	chr11:34894406-34894666:+	526
FJX1	chr11:35596676-35596760:+	5HSAR012218	chr11:35596640-35596726:+	450
PRR5L	chr11:36379171-36379247:+	5HSAR059970	chr11:36274321-36274437:+	125
PTDSS2	chr11:440358-440455:+	5HSAR023109	chr11:440282-440401:+	176
CHST1	chr11:45629050-45630169:-	5HSAR018406	chr11:45629091-45643421:-	671
SLC35C1	chr11:45782593-45783967:+	5HSAR022094	chr11:45782404-45783947:+	477
CREB3L1	chr11:46256166-46256238:+	5HSAR013067	chr11:46255945-46256040:+	435
DGKZ	chr11:46325693-46325785:+	5HSAR016673	chr11:46325562-46325720:+	254
ZNF408	chr11:46679081-46679173:+	5HSAR042160	chr11:46678923-46679108:+	281
PACSIN3	chr11:47160855-47161184:-	5HSAR060119	chr11:47161173-47163915:-	343
LOC340970	chr11:48970010-48972687:-	5HSAR045106	chr11:48970013-48972679:-	134
FOLH1	chr11:49186538-49186628:-	5HSAR024358	chr11:49186655-49186762:-	261
SPRYD5	chr11:55407361-55409480:+	5HSAR020643	chr11:55407356-55407427:+	92
TRIM34	chr11:5612559-5612652:+	5HSAR003010	chr11:5610138-5611640:+<br>chr11:5612504-5612587:+	801
SLC43A3	chr11:56950222-56950313:-	5HSAR000418	chr11:56950243-56950380:-	283

MPEG1	chr11:58736915-58736993:-	5HSAR016578	chr11:58736945-58737019:-	156
OSBP	chr11:59139714-59139820:-	5HSAR011081	chr11:59140071-59140184:-<br>chr11:59139943-59140044:-	480
STX3	chr11:59279575-59279654:+	5HSAR041771	chr11:59279178-59279288:+	547
MS4A3	chr11:59580788-59591017:+	5HSAR010525	chr11:59580701-59588341:+	188
MS4A4A	chr11:59806721-59816289:+	5HSAR002886	chr11:59806675-59806764:+	143
MS4A13	chr11:60040052-60042132:+	5HSAR023754	chr11:60039499-60039648:+<br>chr11:60039650-60040100:+	323
CYBASC3	chr11:60880762-60880859:-	5HSAR036183	chr11:60880777-60880902:-	176
TMEM216	chr11:60917310-60917945:+	5HSAR038773	chr11:60916647-60917290:+<br>chr11:60917303-60917380:+	436
ASRGL1	chr11:61861619-61862025:+	5HSAR026127	chr11:61861445-61861522:+	284
FAM160A2	chr11:6202323-6202412:-	5HSAR014268	chr11:6212357-6212425:-<br>chr11:6202356-6202445:-	359
SLC22A24	chr11:62667828-62667923:-	5HSAR015878	chr11:62668117-62668227:-	442
LGALS12	chr11:63032561-63032648:+	5HSAR016564	chr11:63032067-63032135:+<br>chr11:63032284-63032463:+	614
SLC22A11	chr11:64079947-64080047:+	5HSAR019545	chr11:64079755-64079850:+	374
NRXN2	chr11:64166852-64166949:-	5HSAR018915	chr11:64167012-64167110:-	512
DNHD1	chr11:6475927-6476021:+	5HSAR019450	chr11:6475587-6475811:+<br>chr11:6475924-6476007:+	564
SLC25A45	chr11:64903941-64906538:-	5HSAR010514	chr11:64906590-64906682:-	275
CCDC85B	chr11:65414753-65414830:+	5HSAR001020	chr11:65414499-65414585:+	380
CNIH2	chr11:65802404-65802503:+	5HSAR001418	chr11:65802278-65802427:+	232
LRFN4	chr11:66381689-66381791:+	5HSAR011960	chr11:66381540-66381668:+	340
PITPNM1	chr11:67028228-67029300:-	5HSAR012350	chr11:67029258-67029371:-	226
MYEOV	chr11:68818567-68819397:+	5HSAR013658	chr11:68818240-68818308:+<br>chr11:68818346-68818525:+	450
ZNF215	chr11:6909992-6910079:+	5HSAR002607	chr11:6904360-6904425:+<br>chr11:6905560-6909935:+	588

FGF3	chr11:69342639-69342736:-	5HSAR020978	chr11:69342866-69343039:-	491
LOC100131539	chr11:71210171-71213944:-	5HSAR045124	chr11:71213952-71231982:-	421
RNF121	chr11:71317660-71317755:+	5HSAR013533	chr11:71317510-71317587:+	340
LRTOMT	chr11:71477046-71477777:+	5HSAR026920	chr11:71469478-71477008:+	378
C11orf51	chr11:71499971-71501347:-	5HSAR012464	chr11:71500158-71501436:-	222
SYT9	chr11:7229894-7229993:+	5HSAR003026	chr11:7229814-7229897:+	237
P2RY2	chr11:72616781-72622852:+	5HSAR017918	chr11:72616711-72616812:+	333
P2RY6	chr11:72684468-72685211:+	5HSAR022078	chr11:72658732-72658836:+<br>chr11:72658847-72661078:+	542
GDPD4	chr11:76673831-76675951:-	5HSAR002037	chr11:76673851-76676099:-	251
TUB	chr11:8016914-8016996:+	5HSAR020445	chr11:8016809-8016955:+	241
LOC100130431	chr11:85420226-85457366:+	5HSAR045130	chr11:85420180-85420245:+	187
C11orf17	chr11:8889472-8889572:+	5HSAR011086	chr11:8889380-8889532:+	195
CHID1	chr11:894817-898597:-	5HSAR016833	chr11:898568-900780:-	247
DENND5A	chr11:9243193-9243295:-	5HSAR023046	chr11:9243296-9243397:-	255
ZNF143	chr11:9439110-9449431:+	5HSAR058354	chr11:9439108-9449428:+	119
MTMR2	chr11:95238459-95255247:-	5HSAR018341	chr11:95296844-95297017:-<br>chr11:95287121-95296766:-	663
GNPTAB	chr12:100748585-100748675:-	5HSAR041558	chr12:100748592-100748717:-	192
CLEC9A	chr12:10095924-10096553:+	5HSAR018811	chr12:10074582-10074686:+<br>chr12:10074696-10074818:+	613
GLT8D2	chr12:102937557-102939312:-	5HSAR003779	chr12:102939331-102967928:-	406
CHST11	chr12:103375215-103375319:+	5HSAR043479	chr12:103374901-103375203:+	498
NUAK1	chr12:105056562-105056643:-	5HSAR015225	chr12:105057477-105057824:-<br>chr12:105057219-105057407:-	1380
MTERFD3	chr12:105896623-105903045:-	5HSAR000779	chr12:105904886-105905050:-<br>chr12:105896661-105903072:-	341

CRY1	chr12:106010870-106010965:-	5HSAR012092	chr12:106011386-106011505:- chr12:106011075-106011146:-	859
ALKBH2	chr12:108014975-108015068:-	5HSAR022246	chr12:108015495-108015599:- chr12:108015036-108015122:-	393
UBE3B	chr12:108403870-108405739:+	5HSAR012335	chr12:108399839-108403833:+	258
TCHP	chr12:108822842-108822919:+	5HSAR022901	chr12:108822780-108822860:+	214
TCTN1	chr12:109536533-109538500:+	5HSAR051176	chr12:109536294-109536374:+	412
FAM109A	chr12:110287754-110288310:-	5HSAR053092	chr12:110288417-110291163:- chr12:110287760-110288388:-	440
ALDH2	chr12:110689074-110689169:+	5HSAR031457	chr12:110688756-110688986:+	441
TRAFD1	chr12:111047732-111052709:+	5HSAR021120	chr12:111047935-111048039:+	86
TBX5	chr12:113326087-113330055:-	5HSAR031160	chr12:113330378-113330629:- chr12:113330136-113330216:-	667
CCDC60	chr12:118257276-118257364:+	5HSAR022743	chr12:118256972-118257064:+	465
RPLP0	chr12:119122970-119123062:-	5HSAR020422	chr12:119123320-119123385:- chr12:119122982-119123062:-	237
ANAPC5	chr12:120274527-120274625:-	5HSAR016218	chr12:120274539-120274607:-	122
RNF34	chr12:120322363-120324984:+	5HSAR012836	chr12:120322349-120324964:+	177
WDR66	chr12:120840933-120843594:+	5HSAR059076	chr12:120840855-120840941:+	172
BCL2L14	chr12:12115763-12123506:+	5HSAR011462	chr12:12115670-12115741:+ chr12:12115749-12115823:+	195
KNTC1	chr12:121577821-121580563:+	5HSAR013190	chr12:121577765-121577836:+	163
C12orf65	chr12:122283957-122304171:+	5HSAR018368	chr12:122283873-122283974:+	263
UBC	chr12:123964271-123965170:-	5HSAR020773	chr12:123965086-123965175:-	458
PUS1	chr12:130980125-130980220:+	5HSAR039764	chr12:130979791-130979907:+	455
GOLGA3	chr12:131908788-131908874:-	5HSAR037616	chr12:131908824-131908907:-	221
H2AFJ	chr12:14818573-14818671:+	5HSAR003397	chr12:14818544-14818669:+	135
WBP11	chr12:14845585-14847584:-	5HSAR001857	chr12:14847574-14847666:-	161

PTPRO	chr12:15366845-15366927:+	5HSAR029975	chr12:15366803-15366868:+	174
LMO3	chr12:16645062-16650337:-	5HSAR018964	chr12:16650526-16650618:-<br>chr12:16650271-16650462:-	440
SLCO1A2	chr12:21378849-21378944:-	5HSAR017818	chr12:21378940-21379059:-	251
RECQL	chr12:21543772-21545622:-	5HSAR021040	chr12:21545677-21545832:-	348
C12orf39	chr12:21570577-21570673:+	5HSAR031481	chr12:21570531-21570635:+	151
GYS2	chr12:21648794-21648879:-	5HSAR029078	chr12:21648833-21648937:-	255
SOX5	chr12:23940225-24412766:-	5HSAR029583	chr12:24321480-24451694:-	371
BCAT1	chr12:24946167-24946261:-	5HSAR042091	chr12:24946495-24946584:-<br>chr12:24946359-24946448:-	423
C12orf77	chr12:25041436-25041514:-	5HSAR000754	chr12:25041533-25041604:-	205
IFLTD1	chr12:25597161-25597342:-	5HSAR022741	chr12:25597346-25597429:-	245
C12orf70	chr12:27511106-27514831:+	5HSAR020517	chr12:27511035-27511124:+	176
PPFIBP1	chr12:27622381-27677596:+	5HSAR019904	chr12:27568378-27622406:+	377
PTHLH	chr12:28013695-28013944:-	5HSAR028890	chr12:28013885-28014049:-	303
TMTC1	chr12:29812254-29828586:-	5HSAR002257	chr12:29828720-29828896:-	474
FAM60A	chr12:31342406-31349254:-	5HSAR014612	chr12:31349221-31349310:-	322
ABCD2	chr12:38299685-38299777:-	5HSAR017857	chr12:38299897-38300082:-<br>chr12:38299763-38299870:-	426
CCNB1IP1	chr12:384977-391231:+	5HSAR014229	chr14:19863559-19864728:-	275
ZCRB1	chr12:41004172-41006090:-	5HSAR003745	chr12:41005995-41006093:-	209
PRICKLE1	chr12:41152586-41163494:-	5HSAR026153	chr12:41163612-41163683:-	287
PUS7L	chr12:42435316-42438828:-	5HSAR012370	chr12:42438722-42438823:-<br>chr12:42435320-42438412:-	75
RPAP3	chr12:46376394-46377676:-]	5HSAR024240	chr12:46381552-46381635:-<br>chr12:46377679-46381545:-	434
RAPGEF3	chr12:46438009-46438107:-	5HSAR029519	chr12:46438318-46438398:-<br>chr12:46438076-46438159:-	440

FKBP11	chr12:47604284-47604668:-	5HSAR028927	chr12:47604617-47604685:-	219
PRKAG1	chr12:47685413-47685614:-	5HSAR024402	chr12:47693144-47698857:-<br>chr12:47685837-47693126:-	370
LMBR1L	chr12:47790606-47790696:-	5HSAR021401	chr12:47790778-47790921:-	342
KCNA6	chr12:4789374-4789468:+	5HSAR012754	chr12:4788638-4788811:+	866
KCNH3	chr12:48219361-48219466:+	5HSAR002433	chr12:48219266-48219364:+	260
PRPF40B	chr12:48304122-48304220:+	5HSAR000128	chr12:48303717-48304214:+	551
NCKAP5L	chr12:48484095-48486069:-	5HSAR014321	chr12:48486062-48508422:-	202
LOC100131403	chr12:48634385-48642799:-	5HSAR007103	chr12:48642843-48642908:-	270
ATF1	chr12:49444313-49460195:+	5HSAR017209	chr12:49444173-49444262:+	308
SLC4A8	chr12:50104939-50105038:+	5HSAR013560	chr12:50104875-50104958:+	178
ANKRD33	chr12:50568151-50568237:+	5HSAR011823	chr12:50568176-50568241:+	178
C12orf44	chr12:50750519-50753701:+	5HSAR026031	chr12:50750490-50753646:+	304
KRT4	chr12:51494332-51494434:-	5HSAR027320	chr12:51494408-51494515:-	271
LOC100129362	chr12:51629609-51630433:-	5HSAR045182	chr12:51630543-51630614:-	316
TARBP2	chr12:52181360-52181459:+	5HSAR026849	chr12:52180993-52181097:+<br>chr12:52181103-52181279:+	488
HOXC6	chr12:52708721-52708818:+	5HSAR010729	chr12:52697116-52697331:+<br>chr12:52697347-52708675:+	749
SMUG1	chr12:52863992-52867939:-	5HSAR021052	chr12:52864007-52869018:-	129
NFE2	chr12:52975300-52975640:-	5HSAR026487	chr12:52975627-52975731:-	273
PDE1B	chr12:53241536-53241635:+	5HSAR034749	chr12:53241395-53241607:+	270
RNF41	chr12:54890497-54890590:-	5HSAR012299	chr12:54894088-54896625:-<br>chr12:54893153-54894067:-	725
PAN2	chr12:55013146-55013233:-	5HSAR057236	chr12:55013184-55013557:-	377
STAT2	chr12:55036623-55040197:-	5HSAR038716	chr12:55040203-55040274:-	203

STAT6	chr12:55788329-55790199:-	5HSAR052934	chr12:55790136-55790321:-	251
SHMT2	chr12:55910230-55910882:+	5HSAR036702	chr12:55910129-55910209:+	230
INHBC	chr12:56114852-56114936:+	5HSAR021658	chr12:56114819-56114896:+	127
CDK4	chr12:56431768-56432306:-	5HSAR027932	chr12:56432284-56432391:-	226
VWF	chr12:6103848-6103926:-	5HSAR028458	chr12:6103969-6104055:-	250
PLEKHG6	chr12:6292491-6292571:+	5HSAR019388	chr12:6292169-6292285:+<br>chr12:6292412-6292486:+	494
MRPL51	chr12:6472564-6472664:-	5HSAR009313	chr12:6472647-6472715:-	169
YEATS4	chr12:68039927-68040019:+	5HSAR021466	chr12:68039826-68039909:+	221
RAB3IP	chr12:68419801-68419893:+	5HSAR002494	chr12:68419504-68419602:+<br>chr12:68419774-68419848:+	447
KCNMB4	chr12:69046693-69046781:+	5HSAR018040	chr12:69046397-69046534:+	453
PTPRR	chr12:69434241-69434335:-	5HSAR014989	chr12:69434540-69434605:-<br>chr12:69434410-69434520:-	392
TSPAN8	chr12:69837726-69837818:-	5HSAR028158	chr12:69837761-69838031:-	179
TBC1D15	chr12:70519820-70541489:+	5HSAR026615	chr12:70519763-70541481:+	143
CD163	chr12:7547554-7547648:-	5HSAR029732	chr12:7547596-7547679:-	128
SLC2A3	chr12:7979898-7979990:-	5HSAR028048	chr12:7979988-7980068:-	262
CLEC4A	chr12:8167648-8167741:+	5HSAR027982	chr12:8167577-8167651:+	247
TMTC3	chr12:87060342-87066223:+	5HSAR022826	chr12:87060204-87060299:+	220
ATP2B1	chr12:88573795-88573878:-	5HSAR029328	chr12:88573845-88573946:-	181
LOC643153	chr12:88628079-88628177:-	5HSAR045200	chr12:88629608-88629820:-<br>chr12:88629531-88629599:-	1782
DCN	chr12:90096461-90100618:-	5HSAR030233	chr12:90100778-90100864:-<br>chr12:90100708-90100773:-	408
BTG1	chr12:91063443-91063531:-	5HSAR019118	chr12:91063664-91063735:-	362
PLXNC1	chr12:93066790-93066878:+	5HSAR028802	chr12:93066752-93066829:+	249

CCDC41	chr12:93330398-93330490:-	5HSAR010328	chr12:93377608-93377820:- chr12:93330493-93377595:-	533
NEDD1	chr12:95825135-95827668:+	5HSAR027217	chr12:95825521-95825715:+	86
ABHD13	chr13:107668933-107679567:+	5HSAR021046	chr13:107668788-107668916:+	265
LOC100131435	chr13:110163389-110163476:-	5HSAR007345	chr13:110163477-110163653:-	488
ARHGEF7	chr13:110565783-110565874:+	5HSAR022009	chr13:110655644-110655715:+	250
LOC100128430	chr13:113567673-113567769:-	5HSAR046583	chr13:113568382-113568645:- chr13:113568083-113568349:-	978
GAS6	chr13:113576308-113576404:+	5HSAR029646	chr13:113574943-113575416:+	639
ZNF828	chr13:114104771-114107419:+	5HSAR031702	chr13:114104755-114107408:+	293
PSPC1	chr13:19254898-19255001:-	5HSAR025739	chr13:19254983-19255075:-	186
LATS2	chr13:20518166-20518270:-	5HSAR018213	chr13:20518329-20533713:- chr13:20518217-20518306:	442
FGF9	chr13:21143957-21144051:+	5HSAR014626	chr13:21143373-21143531:+ chr13:21143539-21143607:+	837
RASL11A	chr13:26743000-26743081:+	5HSAR016180	chr13:26742693-26742833:+	618
MTUS2	chr13:28900883-28900979:+	5HSAR010552	chr13:28900861-28900944:+	203
SLC7A1	chr13:29008326-29025969:-	5HSAR024944	chr13:29025929-29067816:-	387
NBEA	chr13:34414878-34414957:+	5HSAR049445	chr13:34414441-34414809:+	534
C13orf31	chr13:43351801-43353121:+	5HSAR018919	chr13:43351626-43353091:+	485
FAM194B	chr13:45069142-45079734:-	5HSAR015898	chr13:45069187-45087845:-	165
LRRC63	chr13:45684334-45685170:+	5HSAR045228	chr13:45684294-45684368:+	345
C13orf18	chr13:45844612-45844699:-	5HSAR016310	chr13:45844733-45850129:-	605
FNDC3A	chr13:48448274-48478327:+	5HSAR020237	chr13:48448069-48448149:+	305
CAB39L	chr13:48855048-48873322:-	5HSAR019156	chr13:48873411-48873533:- chr13:48873298-48873390:-	498
SETDB2	chr13:48923943-48924030:+	5HSAR049108	chr13:48916468-48916875:+ chr13:48916932-48923740:+	906

PHF11	chr13:48969289-48978794:+	5HSAR025682	chr13:48968553-48969284:+	276
KCNRG	chr13:49487535-49487630:+	5HSAR027901	chr13:49487463-49487555:+	240
LOC100129303	chr13:50724315-50752640:-	5HSAR045230	chr13:50752652-50752744:-	300
CCDC70	chr13:51334313-51337515:+	5HSAR025994	chr13:51334302-51337475:+	296
UTP14C	chr13:51500857-51500941:+	5HSAR018672	chr13:51496855-51500727:+ ch	733
THSD1	chr13:51874737-51878179:-	5HSAR018690	chr13:51874782-51878610:-	545
DIAPH3	chr13:59635902-59635993:-	5HSAR026468	chr13:59635942-59636061:-	219
PCDH20	chr13:60887293-60887383:-	5HSAR012934	chr13:60887420-60887494:-	364
DIS3	chr13:72253972-72254057:-	5HSAR024216	chr13:72254042-72254266:-	374
TBC1D4	chr13:74953905-74953995:-	5HSAR021182	chr13:74954052-74954156:-	347
FBXL3	chr13:76493997-76499035:-	5HSAR018688	chr13:76499038-76499106:-	371
MYCBP2	chr13:76798912-76799015:-	5HSAR058258	chr13:76799041-76799124:-	267
EDNRB	chr13:77390710-77390792:-	5HSAR031054	chr13:77390829-77390912:-	258
SLITRK1	chr13:83353644-83353746:-	5HSAR026903	chr13:83354067-83354351:- chr13:83353940-83354065:-	886
GPC6	chr13:92677617-92677710:+	5HSAR029039	chr13:92677396-92677467:+	632
DZIP1	chr13:95092285-95092369:-	5HSAR001401	chr13:95093341-95093884:- chr13:95092304-95093323:-	852
RAP2A	chr13:96884626-96884725:+	5HSAR022431	chr13:96884537-96884680:+	250
DOCK9	chr13:98428165-98428254:-	5HSAR019316	chr13:98428184-98428336:-	175
LOC100130814	chr14:100610138-100610239:+	5HSAR007475	chr14:100610132-100610239:+	123
PPP2R5C	chr14:101297888-101297983:+	5HSAR037489	chr14:101345899-101345976:+]	96
HSP90AA1	chr14:101623076-101623164:-	5HSAR030403	chr14:101623081-101623263:-	190
MARK3	chr14:102922028-102922119:+	5HSAR019137	chr14:102921636-102921752:+	666

BAG5	chr14:103097255-103098786:-	5HSAR001870	chr14:103098145-103098258:-	227
XRCC3	chr14:103247178-103247262:-	5HSAR031000	chr14:103247264-103247609:-	323
AKT1	chr14:104330026-104330519:-	5HSAR030799	chr14:104330585-104332935:-	424
GPR132	chr14:104592803-104592896:-	5HSAR011169	chr14:104593353-104594613:-<br>chr14:104593112-104593312:-	899
KLHL33	chr14:19968675-19973512:-	5HSAR034652	chr14:19968688-19973569:-	223
C14orf176	chr14:20639135-20639342:+	5HSAR027743	chr14:20636956-20637111:+<br>chr14:20637143-20639311:+	406
MRPL52	chr14:22369151-22372486:+	5HSAR010265	chr14:22368962-22369054:+	316
JUB	chr14:22514142-22515713:-	5HSAR023048	chr14:22516181-22516252:-<br>chr14:22516000-22516146:-	654
SLC7A8	chr14:22682153-22682250:-	5HSAR010735	chr14:22693323-22693403:-	244
PABPN1	chr14:22860426-22860518:+	5HSAR020226	chr14:22859238-22859348:+<br>chr14:22859443-22859607:+	1282
THTPA	chr14:23095722-23095806:+	5HSAR026473	chr14:23095041-23095193:+<br>chr14:23095245-23095391:+	769
JPH4	chr14:23116503-23116594:-	5HSAR025077	chr14:23117563-23117640:-<br>chr14:23116538-23117272:-	792
DCAF11	chr14:23653934-23654643:+	5HSAR058337	chr14:23653864-23653929:+	280
CHMP4A	chr14:23752615-23752700:-	5HSAR031598	chr14:23752854-23752949:-<br>chr14:23752726-23752815:-	411
ARHGAP5	chr14:31629516-31629626:+	5HSAR021072	chr14:31616323-31629466:+<br>chr14:31629472-31629555:+	315
FAM177A1	chr14:34585018-34585489:+	5HSAR011827	chr14:34583912-34583977:+<br>chr14:34584004-34584964:+	459
PAX9	chr14:36200964-36201045:+	5HSAR039264	chr14:36196730-36200672:+<br>chr14:36200739-36200960:+	726
CTAGE5	chr14:38804256-38804349:+	5HSAR019799	chr14:38805437-38815908:+	123
LRFN5	chr14:41305921-41425578:+	5HSAR020119	chr14:41146519-41146890:+<br>chr14:41146895-41147023:+	1198
C14orf28	chr14:44436441-44439388:+	5HSAR002997	chr14:44436351-44436416:+	266
MDGA2	chr14:46670698-46670787:-	5HSAR021213	chr14:46840461-46882127:-<br>chr14:46756998-46757087:-	747
MGAT2	chr14:49157646-49157736:+	5HSAR015950	chr14:49157352-49157516:+<br>chr14:49157559-49157639:+	498

MAP4K5	chr14:50068518-50068600:-	5HSAR017923	chr14:50068524-50069115:-	320
ABHD12B	chr14:50415221-50415301:+	5HSAR013994	chr14:50408643-50415291:+	208
TMX1	chr14:50776668-50776760:+	5HSAR022284	chr14:50776636-50776743:+	125
DDHD1	chr14:52689567-52689659:-	5HSAR041856	chr14:52689674-52689766:-	230
BMP4	chr14:53488691-53489829:-	5HSAR027863	chr14:53493223-53493288:-<br>chr14:53489818-53493146:-	210
WDHD1	chr14:54545160-54546824:-	5HSAR020151	chr14:54546919-54550029:-<br>chr14:54545165-54546846:-	366
MUDENG	chr14:56805698-56805785:+	5HSAR022251	chr14:56805461-56805544:+<br>chr14:56805667-56805762:+	427
KIAA0586	chr14:57964660-57964735:+	5HSAR021477	chr14:57964545-57964670:+	273
DACT1	chr14:58174585-58174673:+	5HSAR025692	chr14:58174582-58174647:+	164
C14orf135	chr14:59628808-59651183:+	5HSAR019542	chr14:59628512-59628745:+	522
PPM1A	chr14:59786075-59819174:+	5HSAR021391	chr14:59785871-59785945:+	450
TMEM30B	chr14:60817619-60817700:-	5HSAR003095	chr14:60818032-60818217:-<br>chr14:60817786-60817965:-	665
PRKCH	chr14:60858484-60858572:+	5HSAR026333	chr14:60858425-60858505:+	305
KCNH5	chr14:62553325-62638276:-	5HSAR011587	chr14:62553378-62638330:-]	168
WDR89	chr14:63136414-63161465:-	5HSAR020294	chr14:63136415-63161476:-	207
AKAP5	chr14:64004766-64004865:+	5HSAR021716	chr14:64004729-64004863:+	378
FUT8	chr14:65097950-65098034:+	5HSAR015027	chr14:64947612-64947725:+<br>chr14:64947983-64948075:+	1197
GPHN	chr14:66044906-66044998:+	5HSAR009999	chr14:66043888-66044040:+<br>chr14:66044169-66044558:+	1121
FAM71D	chr14:66733761-66734631:+	5HSAR013826	chr14:66725948-66733741:+<br>chr14:66733749-66733826:+	254
ATP6V1D	chr14:66896169-66896264:-	5HSAR001506	chr14:66896254-66896319:-	176
EIF2S1	chr14:66896839-66901237:+	5HSAR023759	chr14:66896798-66896875:+	141
TMEM229B	chr14:67010394-67023539:-	5HSAR009287	chr14:67023568-67023636:-<br>chr14:67023494-67023565:-	410

ZFYVE26	chr14:67352434-67352516:-	5HSAR027830	chr14:67352473-67353046:-	139
C14orf181	chr14:68332765-68332860:-	5HSAR000923	chr14:68332849-68332920:-	179
TTC9	chr14:70178497-70178599:+	5HSAR024235	chr14:70178439-70178525:+	343
RGS6	chr14:71469699-71501261:+	5HSAR015745	chr14:71469546-71469611:+<br>chr14:71469647-71469766:+	254
PNMA1	chr14:73250096-73250182:-	5HSAR019546	chr14:73250157-73250330:-	786
C14orf43	chr14:73276465-73276562:-	5HSAR001587	chr14:73296652-73296753:-<br>chr14:73276549-73296574:-	783
ZNF410	chr14:73428545-73428631:+	5HSAR011891	chr14:73428547-73428627:+	182
KIAA0317	chr14:74222072-74228545:-	5HSAR015956	chr14:74228632-74228727:-<br>chr14:74228499-74228597:-	505
RPS6KL1	chr14:74457998-74458505:-	5HSAR015197	chr14:74458718-74458792:-<br>chr14:74458563-74458697:-	485
PGF	chr14:74491699-74491777:-	5HSAR030603	chr14:74491747-74491848:-	346
C14orf1	chr14:75193599-75196934:-	5HSAR019433	chr14:75197152-75197283:-<br>chr14:75193601-75196961:-	452
ESRRB	chr14:75940527-75975449:+	5HSAR048572	chr14:75907488-75907625:+	371
PTPN21	chr14:88086515-88086608:-	5HSAR016449	chr14:88090793-88090873:-<br>chr14:88086663-88090771:-	331
EML5	chr14:88328601-88328687:-	5HSAR058260	chr14:88328717-88328806:-	249
SMEK1	chr14:91045745-91046209:-	5HSAR013580	chr14:91046313-91046381:-	283
TRIP11	chr14:91575783-91575871:-	5HSAR058407	chr14:91575999-91576127:-	374
SERPINA1	chr14:93919328-93926583:-	5HSAR029599	chr14:93926669-93926752:-	277
LOC100133065	chr15:19238840-19238939:+	5HSAR045287	chr15:19238530-19238667:+<br>chr15:19238700-19238864:+	482
LOC100132293	chr15:19622207-19622315:-	5HSAR045286	chr15:19622635-19622787:-<br>chr15:19622472-19622582:-	752
TUBGCP5	chr15:20384866-20384965:+	5HSAR026213	chr15:20384873-20384941:+	130
C15orf2	chr15:22472005-22472107:+	5HSAR002485	chr15:22471690-22471794:+<br>chr15:22471809-22471934:+	474
SNURF	chr15:22751203-22751288:+	5HSAR003244	chr15:22751175-22751270:+	126

GABRA5	chr15:24665019-24665488:+	5HSAR040794	chr15:24662974-24665004:+	532
CHRFAM7A	chr15:28452528-28452622:-	5HSAR020758	chr15:28459882-28472908:- chr15:28452572-28456599:-	775
PGBD4	chr15:32181934-32182024:+	5HSAR015941	chr15:32181580-32181660:+ chr15:32181664-32181978:+	459
C15orf29	chr15:32243170-32289525:-	5HSAR014010	chr15:32289469-32289552:-	160
ZNF770	chr15:33062928-33062974:-	5HSAR001081	chr15:33067092-33067647:- chr15:33062942-33067073:-	354
GPR176	chr15:37999520-37999620:-	5HSAR012014	chr15:38000019-38000294:- chr15:37999870-37999941:-	866
ZFYVE19	chr15:38886975-38887079:+	5HSAR010235	chr15:38886700-38886765:+ chr15:38886779-38886949:+	514
NDUFAF1	chr15:39476550-39481647:-	5HSAR009713	chr15:39476562-39481636:-	382
MAPKBP1	chr15:39854672-39854765:+	5HSAR015847	chr15:39854054-39854714:+	286
CAPN3	chr15:40481966-40482048:+	5HSAR024872	chr15:40481949-40482017:+	169
TTBK2	chr15:40958108-40999936:-	5HSAR015579	chr15:41000119-41000247:- chr15:40958170-41000036:-	439
LOC729154	chr15:40999534-40999608:+	5HSAR007652	chr15:40998858-40998938:+ chr15:40998945-40999082:+	830
CCNDBP1	chr15:41264889-41264988:+	5HSAR030046	chr15:41264791-41264880:+	231
LCMT2	chr15:41409980-41410056:-	5HSAR003259	chr15:41409993-41410136:-	204
SPATA5L1	chr15:43481836-43481919:+	5HSAR034830	chr15:43481818-43481886:+	109
DTWD1	chr15:47703246-47704656:+	5HSAR019453	chr15:47703162-47703227:+ chr15:47703255-47704637:+	277
ATP8B4	chr15:48186456-48198658:-	5HSAR026646	chr15:48198613-48198696:-	142
TMOD2	chr15:49831181-49845930:+	5HSAR018082	chr15:49831186-49845911:+	221
RAB27A	chr15:53314425-53317954:-	5HSAR019075	chr15:53349679-53349747:-	233
NEDD4	chr15:53996322-53996410:-	5HSAR028769	chr15:53996407-53996514:-	300
TCF12	chr15:54998316-54999403:+	5HSAR018791	chr15:54998172-54998363:+	284
NARG2	chr15:58546202-58547641:-	5HSAR000459	chr15:58558495-58558590:- chr15:58547578-58557468:-	486

IGDCC3	chr15:63457180-63457270:-	5HSAR018366	chr15:63457234-63457368:-	252
C15orf44	chr15:63686772-63690208:-	5HSAR014670	chr15:63690305-63690421:-	336
DIS3L	chr15:64374404-64374489:+	5HSAR025537	chr15:64374380-64374475:+	183
CALML4	chr15:66284769-66284855:-	5HSAR010031	chr15:66285230-66285322:-<br>chr15:66284888-66285007:-	734
UACA	chr15:68781309-68781411:-	5HSAR026950	chr15:68781569-68781658:-<br>chr15:68781423-68781497:-	366
MYO9A	chr15:70125959-70197087:-	5HSAR003177	chr15:70197223-70197297:-	473
SENP8	chr15:70193889-70219018:+	5HSAR031880	chr15:70193678-70193779:+<br>chr15:70193802-70193933:+	333
HCN4	chr15:71447665-71447755:-	5HSAR023861	chr15:71448561-71448653:-<br>chr15:71448239-71448337:-	994
ISLR2	chr15:72210626-72212148:+	5HSAR011973	chr15:72208827-72208919:+<br>chr15:72208935-72209168:+	769
CYP11A1	chr15:72424589-72427330:-	5HSAR030919	chr15:72427405-72445235:-<br>chr15:72427307-72427402:-	703
EDC3	chr15:72754519-72756407:-	5HSAR024825	chr15:72772386-72775378:-<br>chr15:72772291-72772362:-	488
RPP25	chr15:73035978-73036064:-	5HSAR015653	chr15:73036302-73036475:-]	851
C15orf39	chr15:73281410-73285442:+	5HSAR012442	chr15:73281398-73285408:+	230
PSMA4	chr15:76619923-76623590:+	5HSAR014981	chr15:76619828-76621312:+	208
ANKRD34C	chr15:77362579-77372681:+	5HSAR027727	chr15:77362284-77362379:+<br>chr15:77362569-77372676:+	469
MESDC1	chr15:79081561-79081667:+	5HSAR012200	chr15:79080564-79080806:+<br>chr15:79081015-79081101:+	1318
CPEB1	chr15:81037303-81037489:-	5HSAR026382	chr15:81037322-81037581:-	225
SLC28A1	chr15:83230959-83231995:+	5HSAR031483	chr15:83228927-83230924:+	202
AP3S2	chr15:88238186-88238262:-	5HSAR011333	chr15:88238523-88238603:-	436
SEMA4B	chr15:88545719-88545814:+	5HSAR003807	chr15:88529287-88545758:+	283
SV2B	chr15:89444505-89596054:+	5HSAR043178	chr15:89444314-89444469:+	414
RGMA	chr15:91417231-91417882:-	5HSAR050555	chr15:91417857-91417934:-	161

MCTP2	chr15:92700446-92702780:+	5HSAR030297	chr15:92700232-92700306:+<br>chr15:92700345-92700527:+	368
FAM169B	chr15:96846650-96874516:-	5HSAR011536	chr15:96874584-96875120:-<br>chr15:96846669-96874579:-	
LASS3	chr15:98859578-98879482:-	5HSAR013821	chr15:98886747-98902444:-<br>chr15:98879474-98886652:-	424
LINS1	chr15:98938571-98938671:-	5HSAR010370	chr15:98938632-98959887:-	182
CHSY1	chr15:99609185-99609282:-	5HSAR059432	chr15:99609271-99609642:-	476
GRIN2A	chr16:10181770-10183309:-	5HSAR026961	chr16:10184035-10184109:-	209
CLEC16A	chr16:10945986-10946075:+	5HSAR030566	chr16:10945917-10946048:+	230
BFAR	chr16:14634361-14645704:+	5HSAR031514	chr16:14634243-14645632:+<br>chr16:14645636-14645701:+	281
HAGH	chr16:1816790-1816892:-	5HSAR029954	chr16:1816883-1817191:-	407
IQCK	chr16:19637041-19637129:+	5HSAR000049	chr16:19635340-19636578:+<br>chr16:19636739-19637011:+	699
ACSM2B	chr16:20483669-20495114:-	5HSAR015719	chr16:20495106-20495189:-	175
TRAF7	chr16:2145813-2153922:+	5HSAR002132	chr16:2145815-2153907:+	99
METTL9	chr16:21518453-21518555:+	5HSAR000784	chr16:21518435-21518530:	199
LOC100129478	chr16:221049-221133:+	5HSAR007952	chr16:220534-220599:+<br>chr16:220609-220683:+	604
POLR3E	chr16:22216312-22221790:+	5HSAR002077	chr16:22216262-22221762:+	156
CEMP1	chr16:2521076-2521170:-	5HSAR010787	chr16:2521256-2521399:-<br>chr16:2521118-2521183:-	356
SRRM2	chr16:2742788-2746366:+	5HSAR002796	chr16:2742334-2742507:+	549
LOC100132779	chr16:2756755-2756829:-	5HSAR045365	chr16:2758065-2758172:-<br>chr16:2757865-2758008:-	1418
SULT1A1	chr16:28527678-28527773:-	5HSAR031133	chr16:28528021-28528143:-<br>chr16:28527850-28527927:-	473
FLYWCH2	chr16:2886360-2886451:+	5HSAR016533	chr16:2873248-2885247:+	371
SPNS1	chr16:28893898-28893973:+	5HSAR020538	chr16:28893602-28893787:+	377
PRRT2	chr16:29731117-29731876:+	5HSAR001576	chr16:29731013-29731849:+	301

THOC6	chr16:3014250-3014328:+	5HSAR016375	chr16:3014157-3014240:+	296
ZNF771	chr16:30326408-30326875:+	5HSAR016358	chr16:30326358-30326429:+	260
LOC100131892	chr16:30364078-30364178:+	5HSAR045403	chr16:30364056-30364130:+	138
ZNF689	chr16:30528864-30528966:-	5HSAR003521	chr16:30529033-30529173:-	320
PRR14	chr16:30569916-30570467:+	5HSAR000071	chr16:30569847-30569933:+	258
SETD1A	chr16:30876709-30877553:+	5HSAR019304	chr16:30876323-30876550:+<br>chr16:30876633-30877543:+	686
ZNF646	chr16:30993448-30995146:+	5HSAR016529	chr16:30993291-30993386:+	289
LOC100128100	chr16:31943452-31943554:-	5HSAR045406	chr16:31943527-31943841:-	412
ZNF200	chr16:3223757-3225004:-	5HSAR022553	chr16:3225284-3225397:-	551
ZNF263	chr16:3273721-3273819:+	5HSAR016335	chr16:3273607-3273774:+	332
LOC100133017	chr16:32887012-32887114:-	5HSAR045409	chr16:32887087-32887401:-	412
LOC100132856	chr16:33595630-33595732:+	5HSAR045411	chr16:33595343-33595657:+	412
ZNF434	chr16:3380126-3380454:-	5HSAR003235	chr16:3387709-3391023:-<br>chr16:3387564-3387698:-	802
DNASE1	chr16:3644965-3645375:+	5HSAR029203	chr16:3643043-3643297:+<br>chr16:3643388-3643513:+	2107
TMEM8A	chr16:371823-371893:-	5HSAR003362	chr16:371844-371942:-	129
ADCY9	chr16:4105445-4105742:-	5HSAR022751	chr16:4105850-4105924:-	539
NMRAL1	chr16:4464135-4464614:-	5HSAR030226	chr16:4464821-4464889:-	376
PHKB	chr16:46052803-46055350:+	5HSAR010590	chr16:46052763-46055310:+	185
RAB11FIP3	chr16:464958-465031:+	5HSAR016334	chr16:464896-464961:+	181
N4BP1	chr16:47201385-47201475:-	5HSAR025177	chr16:47201467-47201544:-	237
CYLD	chr16:49341016-49341110:+	5HSAR022100	chr16:49334196-49336164:+<br>chr16:49336172-49336258:+	415
IRX3	chr16:52877464-52877560:-	5HSAR026685	chr16:52877517-52877720:-	416

MMP2	chr16:54073190-54073288:+	5HSAR031237	chr16:54072975-54073070:+ chr16:54073074-54073205:+	314
NHLRC4	chr16:557962-558048:+	5HSAR000048	chr16:557690-557821:+ chr16:557858-557977:+	596
SLC38A7	chr16:57271532-57271614:-	5HSAR001058	chr16:57275479-57276175:-	393
NAE1	chr16:65414987-65417967:-	5HSAR012862	chr16:65417963-65422303:-	240
CES8	chr16:65591996-65592075:+	5HSAR053448	chr16:65591919-65591999:+	159
NOL3	chr16:65765364-65765573:+	5HSAR049740	chr16:65762652-65762762:+ ch	193
KIAA0895L	chr16:65772015-65774690:-	5HSAR016461	chr16:65774870-65774965:- chr16:65774699-65774764:-	775
EXOC3L	chr16:65781081-65781458:-	5HSAR000428	chr16:65781470-65781598:-	241
LRRC29	chr16:65801593-65801683:-	5HSAR002516	chr16:65801657-65815243:-	260
ZDHHC1	chr16:65999180-66007630:-	5HSAR016356	chr16:66007674-66007739:-	304
PSKH1	chr16:66484760-66500153:+	5HSAR015099	chr16:66484680-66500089:+	170
DDX28	chr16:66614607-66614688:-	5HSAR035902	chr16:66615105-66615218:- chr16:66614909-66615094:-	665
STUB1	chr16:670422-670526:+	5HSAR028286	chr16:670174-670257:+	411
IL34	chr16:69238259-69238351:+	5HSAR035360	chr16:69215416-69238250:+	538
VAC14	chr16:69392305-69392386:-	5HSAR017089	chr16:69392422-69392550:- chr16:69392334-69392408:-	258
FTSJD1	chr16:69877325-69880497:-]	5HSAR012250	chr16:69877332-69880606:-	406
ZNF23	chr16:70045480-70045570:-	5HSAR013329	chr16:70053167-70053325:- chr16:70045616-70048200:-	814
CHST4	chr16:70117641-70128081:+	5HSAR058306	chr16:70117560-70117643:+	211
ZFHX3	chr16:71551546-71639191:-	5HSAR021434	chr16:71639519-71639686:- chr16:71551567-71639366:-	673
BCAR1	chr16:73829493-73829590:-	5HSAR052069	chr16:73830311-73830382:- chr16:73830023-73830136:-	989
CFDP1	chr16:74024752-74024854:-	5HSAR011059	chr16:74024779-74024886:-	137
CHST5	chr16:74121784-74121872:-	5HSAR009604	chr16:74122971-74123039:- chr16:74122760-74122843:-	1395

CENPN	chr16:79598321-79603045:+	5HSAR009592	chr16:79598024-79598107:+<br>chr16:79598148-79598327:+	790
BCMO1	chr16:79829917-79830014:+	5HSAR031590	chr16:79829799-79829897:+	218
HSD17B2	chr16:80626441-80626530:+	5HSAR023324	chr16:80626372-80626509:+	172
MBTPS1	chr16:82692890-82692975:-	5HSAR020008	chr16:82693191-82707916:-<br>chr16:82693005-82693157:-	502
KLHL36	chr16:83239691-83241974:+	5HSAR011858	chr16:83239645-83239743:+	141
FOXL1	chr16:85169728-85169830:+	5HSAR013066	chr16:85169671-85169778:+	215
C16orf68	chr16:8626935-8627023:+	5HSAR010760	chr16:8623068-8626883:+<br>chr16:8626901-8626966:+	276
RNF166	chr16:87300235-87300327:-	5HSAR060056	chr16:87296826-87297170:-<br>chr16:87293638-87296729:-	96
RPL13	chr16:88154769-88154868:+	5HSAR017103	chr16:88154698-88154862:+	237
CDK10	chr16:88283194-88284514:+	5HSAR043684	chr16:88280620-88283192:+	218
CENPBD1	chr16:88565832-88565929:-	5HSAR016002	chr16:88566019-88566258:-<br>chr16:88565867-88565980:-	612
CARHSP1	chr16:8860687-8869484:-	5HSAR010447	chr16:8869545-8869664:-	350
ZNF18	chr17:11836872-11839607:-	5HSAR019666	chr17:11841005-11841346:-<br>chr17:11840915-11841004:-	605
MYOCD	chr17:12510146-12510230:+	5HSAR028288	chr17:12509947-12510036:+<br>chr17:12510124-12510222:+	299
HS3ST3A1	chr17:13445172-13445268:-	5HSAR020893	chr17:13445433-13445591:-<br>chr17:13445210-13445428:-	798
INPP5K	chr17:1363530-1363924:-	5HSAR020319	chr17:1366641-1366838:-<br>chr17:1366138-1366543:-	848
HS3ST3B1	chr17:14145459-14145560:+	5HSAR020898	chr17:14145360-14145449:+	330
CDRT1	chr17:15463552-15463643:-	5HSAR009707	chr17:15463575-15463700:-	192
TRIM16	chr17:15495649-15495740:-	5HSAR028754	chr17:15495886-15526913:-<br>chr17:15495714-15495800:-	557
ZNF287	chr17:16411771-16411863:-	5HSAR012004	chr17:16411773-16411844:-	453
ALKBH5	chr17:18028196-18028282:+	5HSAR010349	chr17:18027598-18027918:+<br>chr17:18028012-18028095:+	691
FBXW10	chr17:18588197-18588282:+	5HSAR010458	chr17:18588062-18588259:+	232

SLC47A2	chr17:19560461-19560558:-	5HSAR002094	chr17:19560479-19560598:-	175
ALDH3A1	chr17:19589035-19589446:-	5HSAR028347	chr17:19589424-19589504:-	175
ULK2	chr17:19711323-19711415:-	5HSAR019477	chr17:19711617-19711685:- ch	509
AKAP10	chr17:19821585-19821668:-	5HSAR025467	chr17:19821628-19821705:-	137
LOC100128559	chr17:21736983-21737085:+	5HSAR008176	chr17:21736352-21736426:+ chr17:21736447-21736527:+	771
TSR1	chr17:2186472-2186563:-	5HSAR000662	chr17:2187185-2187409:- chr17:2186779-2186883:-	957
RAB34	chr17:24068412-24068503:-	5HSAR029058	chr17:24068745-24068828:-	624
EVI2A	chr17:26670158-26672717:-	5HSAR048895	chr17:26672710-26672844:-	265
TMEM98	chr17:28279333-28282659:+	5HSAR013951	chr17:28279284-28279367:+	382
ACCN1	chr17:28643247-28643332:-	5HSAR012238	chr17:28643664-28643729:-	873
NLE1	chr17:30487582-30488178:-	5HSAR002011	chr17:30493321-30493407:- chr17:30491096-30493235:-	1039
SLFN11	chr17:30714940-30718137:-	5HSAR025716	chr17:30718095-30724813:-	364
PEX12	chr17:30929154-30929239:-	5HSAR013152	chr17:30929176-30929268:-	616
CCL15	chr17:31352645-31352749:-	5HSAR024840	chr17:31353022-31353114:- chr17:31352730-31352813:-	569
TBC1D3G	chr17:31880632-31882137:-	5HSAR000804	chr17:31882131-31882214:-	157
ACACA	chr17:32761453-32776337:-	5HSAR025209	chr17:32840839-32840916:- chr17:32800504-32840640:-	1298
TADA2A	chr17:32845480-32845565:+	5HSAR011470	chr17:32841463-32841552:+ chr17:32841571-32845516:+	369
SPATA22	chr17:3319370-3321505:-	5HSAR055992	chr17:3319416-3321531:-	416
TBC1D3	chr17:33358421-33359926:+	5HSAR031641	chr17:33358344-33358427:+	157
TBC1D3H	chr17:33550663-33552168:-	5HSAR031584	chr17:33552162-33552245:-	157
TBC1D3F	chr17:33600889-33602376:-	5HSAR042850	chr17:33602370-33602453:-	157
TRPV1	chr17:3442394-3442487:-	5HSAR022414	chr17:3442607-3442720:-	527

CTNS	chr17:3487283-3490249:+	5HSAR017079	chr17:3486523-3486783:+<br>chr17:3486832-3487286:+	593
ERBB2	chr17:35104902-35116785:+	5HSAR030135	chr17:35098012-35103053:+	520
THRA	chr17:35484183-35484267:+	5HSAR056232	chr17:35471990-35472106:+<br>chr17:35472133-35484160:+	556
CASC3	chr17:35550241-35550327:+	5HSAR018741	chr17:35550215-35550295:+	295
RARA	chr17:35752390-35752482:+	5HSAR043728	chr17:35751841-35752095:+	686
TMEM99	chr17:36244200-36244294:+	5HSAR010313	chr17:36242893-36242970:+<br>chr17:36244181-36244246:+	307
KRT23	chr17:36346382-36346461:-	5HSAR013729	chr17:36347187-36347357:-<br>chr17:36346607-36347148:-	589
KRT40	chr17:36394052-36396854:-	5HSAR048471	chr17:36396149-36396901:-	161
NAGLU	chr17:37941736-37941816:+	5HSAR022752	chr17:37941492-37941566:+	340
COASY	chr17:37967682-37968166:+	5HSAR014545	chr17:37967956-37968042:+<br>chr17:37968531-37968641:+	157
TUBG1	chr17:38015189-38015281:+	5HSAR019464	chr17:38014935-38015108:+	398
TUBG2	chr17:38064971-38065047:+	5HSAR013440	chr17:38064856-38065005:+	256
BECN1	chr17:38229422-38229765:-	5HSAR027393	chr17:38229718-38229792:-	162
BRCA1	chr17:38512070-38529637:-	5HSAR053392	chr17:38512076-38529639:-	281
ETV4	chr17:38978512-38978781:-	5HSAR028759	chr17:38979155-38979259:-	128
LSM12	chr17:39499482-39499567:-	5HSAR015813	chr17:39499586-39500426:-	323
GRN	chr17:39778144-39782058:+	5HSAR025110	chr17:39778106-39778177:+	219
DBF4B	chr17:40141620-40141714:+	5HSAR018738	chr17:40141594-40141662:+	213
HIGD1B	chr17:40280939-40281046:+	5HSAR023649	chr17:40280809-40280928:+	242
HEXIM1	chr17:40582236-40582340:+	5HSAR023382	chr17:40580497-40580586:+<br>chr17:40580980-40581060:+	1874
HEXIM2	chr17:40595733-40595943:+	5HSAR003721	chr17:40594072-40595738:+	239
TBKBP1	chr17:43128401-43128477:+	5HSAR022854	chr17:43128103-43128318:+	849

SNX11	chr17:43540171-43544404:+	5HSAR001238	chr17:43540151-43543109:+	354
GNGT2	chr17:44639784-44641259:-	5HSAR022190	chr17:44641284-44641694:-	307
PHOSPHO1	chr17:44657486-44659093:-	5HSAR026524	chr17:44662992-44663063:-	418
ZNF652	chr17:44750087-44750182:-	5HSAR021852	chr17:44750275-44794322:-<br>chr17:44750190-44750255:-	464
SPOP	chr17:45055172-45100419:-	5HSAR023263	chr17:45100388-45110460:-	349
DLX4	chr17:45405271-45405368:+	5HSAR023204	chr17:45401597-45401674:+	240
CACNA1G	chr17:45993730-45993819:+	5HSAR023202	chr17:45993645-45993752:+	372
GPR172B	chr17:4878626-4878715:-	5HSAR014574	chr17:4879027-4879293:-	679
ZFP3	chr17:4922517-4935523:+	5HSAR003201	chr17:4922487-4922567:+	125
ZNF232	chr17:4955842-4955938:-	5HSAR002275	chr17:4956312-4967110:-<br>chr17:4956155-4956238:-	655
ZNF594	chr17:5028276-5035831:-	5HSAR010524	chr17:5028289-5035849:-	156
HLF	chr17:50697750-50697844:+	5HSAR023774	chr17:50697342-50697539:+<br>chr17:50697675-50697785:+	525
CUEDC1	chr17:53317925-53318008:-	5HSAR000007	chr17:53335571-53335687:-<br>chr17:53318090-53335450:-	719
VEZF1	chr17:53420472-53420564:-	5HSAR013280	chr17:53420525-53420593:-	143
MPO	chr17:53713119-53713211:-	5HSAR030620	chr17:53713156-53713251:-	177
RNF43	chr17:53847938-53848034:-	5HSAR016890	chr17:53849720-53849824:-<br>chr17:53849633-53849710:-	939
TRIM37	chr17:54538605-54538708:-	5HSAR011395	chr17:54538718-54539032:-	444
SKA2	chr17:54587307-54587379:-	5HSAR000764	chr17:54587408-54587545:-	276
C17orf82	chr17:56844016-56844118:+	5HSAR003761	chr17:56843944-56844093:+	225
CCDC47	chr17:59197268-59204572:-	5HSAR009843	chr17:59204686-59204817:-	336
WSCD1	chr17:5924622-5924702:+	5HSAR000514	chr17:5924431-5924535:+	327
FTSJ3	chr17:59257995-59258199:-	5HSAR013171	chr17:59258637-59258729:-<br>chr17:59258391-59258477:-	646

ICAM2	chr17:59437784-59438866:-	5HSAR021945	chr17:59438904-59451585:-	355
CCDC46	chr17:61252880-61252970:-	5HSAR011938	chr17:61252903-61253082:-	244
HELZ	chr17:62645383-62670387:-	5HSAR025654	chr17:62670402-62671781:-<br>chr17:62645386-62670391:-	187
PITPNC1	chr17:62804628-62804732:+	5HSAR001240	chr17:62804391-62804474:+	347
ABCA9	chr17:64558863-64568677:-	5HSAR018073	chr17:64568626-64568724:-	143
ABCA6	chr17:64648440-64649526:-	5HSAR018074	chr17:64648440-64649579:-	175
KIAA0753	chr17:6479148-6484706:-	5HSAR010571	chr17:6484886-6484960:-<br>chr17:6479184-6484864:-	359
SSTR2	chr17:68673021-68677053:+	5HSAR027966	chr17:68672766-68672867:+<br>chr17:68676962-68677033:+	360
CD300C	chr17:70053517-70053608:-	5HSAR013534	chr17:70053737-70053874:-	361
SLC16A5	chr17:70596941-70601326:+	5HSAR003134	chr17:70596927-70601300:+	287
GGA3	chr17:70751181-70754210:-	5HSAR035444	chr17:70751200-70754209:-	309
DULLARD	chr17:7095340-7095450:-	5HSAR018243	chr17:7095484-7095567:-	380
CASKIN2	chr17:71014804-71015912:-	5HSAR016680	chr17:71015929-71017478:-<br>chr17:71015296-71015919:-	305
PRPSAP1	chr17:71861380-71861466:-	5HSAR011798	chr17:71861631-71861711:-<br>chr17:71861539-71861616:-	446
SOX15	chr17:7433719-7433806:-	5HSAR009828	chr17:7434123-7434194:-<br>chr17:7433971-7434051:-	494
CANT1	chr17:74505300-74505584:-	5HSAR050385	chr17:74505497-74505616:-	300
TP53	chr17:7520638-7531479:-	5HSAR031029	chr17:7531497-7531568:-	197
SLC38A10	chr17:76883317-76883406:-	5HSAR024114	chr17:76883402-76883581:-	375
LSMD1	chr17:7701467-7701547:-	5HSAR002802	chr17:7701748-7701852:-	431
CHD3	chr17:7732961-7733043:+	5HSAR021919	chr17:7732896-7732991:+	150
GNAL	chr18:11679466-11679562:+	5HSAR027866	chr18:11679339-11679470:+	427
C18orf1	chr18:13377646-13377721:+	5HSAR016338	chr18:13377410-13377637:+	668

RNMT	chr18:13720707-13721516:+	5HSAR024566	chr18:13720627-13720710:+	196
ROCK1	chr18:16944870-16944961:-	5HSAR028156	chr18:16945437-16945778:-<br>chr18:16945018-16945200:-	941
METTL4	chr18:2557216-2557309:-	5HSAR002067	chr18:2561212-2561295:-<br>chr18:2557625-2561211:-	780
KIAA1012	chr18:27776726-27776829:-	5HSAR001130	chr18:27776939-27777052:-	364
KLHL14	chr18:28604553-28606680:-	5HSAR002855	chr18:28606757-28606945:-	388
DTNA	chr18:30544323-30589938:+	5HSAR013257	chr18:30652326-30652505:+	215
GALNT1	chr18:31488531-31488624:+	5HSAR016350	chr18:31488541-31488618:+	94
MYL12A	chr18:3237829-3243245:+	5HSAR022769	chr18:3237677-3237898:+	395
LOC727918	chr18:3238056-3238146:-	5HSAR008368	chr18:3239694-3239768:-<br>chr18:3239453-3239518:-	1868
SLC14A2	chr18:41449496-41458627:+	5HSAR025887	chr18:41449113-41449181:+<br>chr18:41449201-41449338:+	816
ST8SIA5	chr18:42590470-42590566:-	5HSAR012310	chr18:42590734-42590889:-	568
DYM	chr18:45210763-45240814:-	5HSAR022597	chr18:45240771-45240896:-	365
RPL17	chr18:45271940-45272031:-	5HSAR016220	chr18:45272184-45272876:-<br>chr18:45272000-45272101:-	341
CXXC1	chr18:46067958-46068059:-	5HSAR018020	chr18:46068514-46068597:-<br>chr18:46068415-46068489:-	733
SMAD4	chr18:46827323-46827414:+	5HSAR030859	chr18:46810581-46810724:+<br>chr18:46827302-46827391:+	538
MEX3C	chr18:46977689-46977783:-	5HSAR042821	chr18:46977778-46977987:-	361
DCC	chr18:48121042-48121155:+	5HSAR049760	chr18:48120747-48120812:+	616
C18orf54	chr18:50139194-50140940:+	5HSAR011558	chr18:50139191-50140933:+	116
TCF4	chr18:51405346-51406334:-	5HSAR024125	chr18:51406722-51406850:-<br>chr18:51406410-51406703:-	612
ZFP161	chr18:5283246-5283987:-	5HSAR018277	chr18:5283261-5283997:-	339
NARS	chr18:53439948-53440041:-	5HSAR015033	chr18:53440050-53440145:-	228
ZNF532	chr18:54683713-54736499:+	5HSAR003411	chr18:54681044-54683546:+<br>chr18:54683569-54683724:+	547

CCDC102B	chr18:64534767-64654980:+	5HSAR010501	chr18:64533565-64552965:+	223
CD226	chr18:65765623-65775029:-	5HSAR023395	chr18:65774995-65775171:-	269
NETO1	chr18:68685507-68685586:-	5HSAR003589	chr18:68685601-68685783:-	284
CNDP1	chr18:70352805-70352882:+	5HSAR014558	chr18:70352685-70352861:+	211
KIAA0802	chr18:8707411-8708448:+	5HSAR014107	chr18:8707915-8708447:+	142
LOC100129773	chr18:9526714-9526806:-	5HSAR008371	chr18:9527986-9528096:-<br>chr18:9527740-9527970:-	1399
TXNDC2	chr18:9876075-9876171:+	5HSAR022893	chr18:9875732-9875845:+<br>chr18:9875904-9876161:+	449
TYK2	chr19:10350083-10351356:-	5HSAR027532	chr19:10351324-10352094:-	378
TMEM205	chr19:11317296-11317778:-	5HSAR022172	chr19:11317690-11317782:-	307
LPPR2	chr19:11328567-11329349:+	5HSAR032702	chr19:11327062-11327211:+<br>chr19:11328556-11328639:+	376
CCDC151	chr19:11406838-11406927:-	5HSAR010596	chr19:11406880-11406975:-	143
PRKCSH	chr19:11407511-11407938:+	5HSAR028289	chr19:11407335-11407866:+	336
ZNF627	chr19:11569349-11569442:+	5HSAR018612	chr19:11569249-11569386:+	208
ZNF442	chr19:12335446-12336664:-	5HSAR002467	chr19:12337043-12337403:-<br>chr19:12336833-12336940:-	611
ZNF564	chr19:12523147-12523235:-	5HSAR023252	chr19:12523179-12523316:-	210
IER2	chr19:13124901-13125000:+	5HSAR000081	chr19:13122365-13124824:+	328
CCDC130	chr19:13723530-13723622:+	5HSAR002309	chr19:13723438-13723581:+	217
LOC100131831	chr19:13811922-13812021:-	5HSAR008534	chr19:13812222-13812317:-<br>chr19:13812003-13812107:-	422
PODNL1	chr19:13910016-13910104:-	5HSAR026621	chr19:13910043-13910114:-	274
LPHN1	chr19:14155415-14155508:-	5HSAR015581	chr19:14155444-14177874:-	296
LOC100130932	chr19:14461106-14461199:+	5HSAR045570	chr19:14460707-14460802:+<br>chr19:14460893-14461012:+	536
RAB8A	chr19:16083629-16083711:+	5HSAR018143	chr19:16083500-16083628:+	222

C19orf62	chr19:17239295-17240615:+	5HSAR031496	chr19:17239273-17240602:+	156
FAM129C	chr19:17495145-17495247:+	5HSAR001008	chr19:17495122-17495232:+	138
PDE4C	chr19:18194058-18196064:-	5HSAR014864	chr19:18196171-18196257:-	335
FAM108A1	chr19:1832566-1832652:-]	5HSAR011971	chr19:1832713-1836391:-	406
SSBP4	chr19:18391365-18391458:+	5HSAR033097	chr19:18391330-18391422:+	238
LOC729991	chr19:19163927-19164027:-	5HSAR023341	chr19:19164037-19164255:-	474
CSNK1G2	chr19:1920667-1920771:+	5HSAR025493	chr19:1892251-1892363:+<br>chr19:1920536-1920673:+	523
ZNF682	chr19:19996093-19996182:-	5HSAR010382	chr19:19996100-20010988:-	176
ZNF714	chr19:21072847-21072914:+	5HSAR003651	chr19:21056851-21056967:+<br>chr19:21058639-21058713:+	378
ZNF493	chr19:21397598-21397685:+	5HSAR021121	chr19:21371762-21371854:+<br>chr19:21371880-21397623:+	280
ZNF100	chr19:21742072-21742169:-	5HSAR011808	chr19:21742146-21742262:-	199
ZNF675	chr19:23661676-23661779:-	5HSAR003358	chr19:23661758-23661847:-	182
TIMM13	chr19:2378532-2378625:-	5HSAR011892	chr19:2378649-2378714:-	344
ZNF254	chr19:24061872-24061949:+	5HSAR016017	chr19:24061821-24061895:+	134
TLE6	chr19:2937880-2938064:+	5HSAR018433	chr19:2928538-2928612:+<br>chr19:2933165-2938026:+	400
TBXA2R	chr19:3551633-3551705:-	5HSAR037940	chr19:3551653-3557542:-	387
ZNF507	chr19:37528525-37535576:+	5HSAR015565	chr19:37528395-37528523:+	272
DPY19L3	chr19:37588995-37590999:+	5HSAR037035	chr19:37589013-37590995:+	224
KCTD15	chr19:38981454-38983199:+	5HSAR024638	chr19:38981449-38983199:+	265
ZNF599	chr19:39955587-39955673:-	5HSAR021841	chr19:39955684-39955818:-	388
COX6B1	chr19:40831054-40833985:+	5HSAR034205	chr19:40830979-40831104:+	192
LRFN3	chr19:41120232-41122167:+	5HSAR002435	chr19:41119970-41120083:+<br>chr19:41120215-41120292:+	452

TBCB	chr19:41298208-41298302:+	5HSAR022397	chr19:41297765-41298214:+	575
ZNF529	chr19:41738900-41772402:-	5HSAR023372	chr19:41772362-41787872:-	278
SHD	chr19:4230965-4231060:+	5HSAR020516	chr19:4229609-4229815:+<br>chr19:4230495-4230578:+	1463
ZNF793	chr19:42705344-42706181:+]	5HSAR001790	chr19:42689688-42691087:+<br>chr19:42691104-42702988:+	442
ZNF540	chr19:42734385-42781188:+	5HSAR020318	chr19:42734205-42734297:+<br>chr19:42734306-42781149:+	332
ZNF573	chr19:42941527-42941617:-	5HSAR032064	chr19:42941582-42962061:-	502
PLEKHG2	chr19:44595812-44596566:+	5HSAR009819	chr19:44595650-44595721:+	325
ZNF546	chr19:45195648-45196073:+	5HSAR011781	chr19:45195998-45196063:+	256
ZNF780A	chr19:45287010-45287100:-	5HSAR016625	chr19:45287110-45287947:-<br>chr19:45287016-45287105:-	255
SERTAD3	chr19:45639828-45640170:-	5HSAR011667	chr19:45640164-45640301:-	278
TNFAIP8L1	chr19:4591087-4602881:+	5HSAR058897	chr19:4591099-4591170:+	146
B3GNT8	chr19:46624524-46625081:-	5HSAR019829	chr19:46625082-46626370:-	454
PAFAH1B3	chr19:47498302-47498396:-	5HSAR029859	chr19:47498506-47498628:-	335
MEGF8	chr19:47522139-47522235:+	5HSAR010992	chr19:47521818-47521910:+<br>chr19:47521926-47522000:+	635
KCNN4	chr19:48976854-48976936:-	5HSAR018925	chr19:48976874-48977143:-	396
ZNF283	chr19:49024243-49027373:+	5HSAR001945	chr19:49023434-49024259:+	268
ZNF45	chr19:49118178-49120287:-	5HSAR034234	chr19:49131083-49131181:-<br>chr19:49130938-49131012:-	1091
ZNF223	chr19:49248166-49251169:+	5HSAR002972	chr19:49248049-49248135:+<br>chr19:49248139-49251156:+	253
ZNF180	chr19:49696133-49696223:-	5HSAR020810	chr19:49696251-49696328:-	282
CEACAM19	chr19:49866937-49867043:+	5HSAR011186	chr19:49866633-49866761:+<br>chr19:49866885-49867001:+	480
DMPK	chr19:50977451-50977544:-	5HSAR023879	chr19:50975429-50975557:-<br>chr19:50975269-50975373:-	205
PTPRS	chr19:5237152-5237234:-	5HSAR016430	chr19:5237230-5291736:-	234

DHX34	chr19:52548035-52548127:+	5HSAR036199	chr19:52544383-52547864:+<br>chr19:52547874-52548011:+	349
NAPA	chr19:52710010-52710105:-	5HSAR001984	chr19:52710159-52710290:-	300
SULT2A1	chr19:53081327-53081428:-	5HSAR022858	chr19:53081392-53081457:-	140
KCNJ14	chr19:53650901-53656793:+	5HSAR011023	chr19:53650671-53650883:+	405
DBP	chr19:53832064-53832150:-	5HSAR026805	chr19:53832216-53832284:-	388
PIH1D1	chr19:54646644-54646743:-	5HSAR018457	chr19:54646750-54646815:-	284
FCGRT	chr19:54708380-54708469:+	5HSAR029086	chr19:54707388-54707486:+<br>chr19:54707696-54707812:+	222
IRF3	chr19:54859908-54860788:-	5HSAR024977	chr19:54860798-54860866:-	253
AKT1S1	chr19:55068365-55071762:-	5HSAR012061	chr19:55072273-55072380:-<br>chr19:55072165-55072254:-	726
IZUMO2	chr19:55358264-55358350:-	5HSAR010050	chr19:55358282-55358347:-	87
HSD11B1L	chr19:5637914-5637996:+	5HSAR003810	chr19:5635844-5637934:+	512
FPR3	chr19:56990329-57018813:+	5HSAR030537	chr19:56990247-56990312:+	184
ZNF610	chr19:57542820-57544237:+	5HSAR031691	chr19:57542788-57542871:+	406
ZNF578	chr19:57686417-57696910:+	5HSAR002346	chr19:57652013-57696904:+	244
ZNF808	chr19:57728001-57738349:+	5HSAR001369	chr19:57722774-57738336:+	177
NRTN	chr19:5775089-5775176:+	5HSAR020815	chr19:5774871-5774981:+<br>chr19:5775016-5775174:+	359
FUT6	chr19:5783579-5789757:-	5HSAR014859	chr19:5790413-5790580:-<br>chr19:5790275-5790373:-	1067
ZNF611	chr19:57910960-57923868:-	5HSAR031669	chr19:57910966-57923883:-	175
FUT5	chr19:5818737-5821535:-	5HSAR015142	chr19:5821476-5821541:-	88
BIRC8	chr19:58485440-58485533:-	5HSAR002126	chr19:58486339-58486623:-<br>chr19:58486193-58486318:-	1248
NDUFA11	chr19:5854721-5854812:-	5HSAR022159	chr19:5854830-5854937:-	305
ZNF331	chr19:58758838-58764442:+	5HSAR010081	chr19:58750379-58750462:+<br>chr19:58750643-58758840:+	442

RANBP3	chr19:5929094-5929175:-	5HSAR027500	chr19:5929103-5929318:-	227
MBOAT7	chr19:59383997-59385080:-	5HSAR024264	chr19:59385134-59385511:-	561
TSEN34	chr19:59386024-59387027:+	5HSAR034521	chr19:59385956-59386033:+	187
LILRB3	chr19:59418661-59418737:-	5HSAR018016	chr19:59418706-59418771:-	111
LILRA6	chr19:59438413-59438489:-	5HSAR010535	chr19:59438458-59438523:-	124
ZNF580	chr19:60845589-60845686:+	5HSAR003729	chr19:60845317-60845415:+<br>chr19:60845506-60845592:+	457
CCDC106	chr19:60851401-60852224:+	5HSAR000277	chr19:60850837-60850947:+<br>chr19:60851115-60851261:+	735
NLRP11	chr19:61021353-61035164:-	5HSAR024942	chr19:61039777-61039878:-<br>chr19:61036072-61039716:-	711
NLRP4	chr19:61040086-61055258:+	5HSAR024276	chr19:61039779-61039913:+<br>chr19:61039922-61040101:+	422
ZNF444	chr19:61349531-61350092:+	5HSAR001127	chr19:61344377-61344505:+	367
ZNF543	chr19:62523957-62524033:+	5HSAR050701	chr19:62523751-62523846:+	357
ZNF772	chr19:62680490-62680573:-	5HSAR018397	chr19:62680574-62680669:-	261
ZNF419	chr19:62691038-62691130:+	5HSAR020178	chr19:62690903-62690986:+	240
ZNF606	chr19:63204500-63206005:-	5HSAR002667	chr19:63206400-63206495:-<br>chr19:63205961-63206341:-	618
ZSCAN18	chr19:63292015-63301384:-	5HSAR026661	chr19:63301451-63301528:-	251
ZNF544	chr19:63433626-63447201:+	5HSAR003209	chr19:63431940-63432008:+<br>chr19:63432011-63433585:+	474
TNFSF14	chr19:6621081-6621316:-	5HSAR021260	chr19:6621127-6621431:-	382
ARHGEF18	chr19:7410728-7410826:+	5HSAR018524	chr19:7410581-7410688:+	253
C19orf45	chr19:7468496-7471708:+	5HSAR014418	chr19:7468447-7468521:+	141
2-Sep	chr19:8384252-8392724:+	chr2:241908308-	chr2:241908308-241912304:+	134
MUC16	chr19:8952815-8952907:-	5HSAR028002	chr19:8952862-8952948:-	204
ZNF559	chr19:9309484-9310225:+	5HSAR001132	chr19:9295993-9296067:+<br>chr19:9296317-9309511:+	647

ZNF266	chr19:9390215-9390307:-	5HSAR002962	chr19:9406836-9407162:-<br>chr19:9391752-9391868:-	991
C19orf6	chr19:971996-972088:-	5HSAR016256	chr19:972014-972109:-	146
ZNF846	chr19:9736627-9739972:-	5HSAR011854	chr19:9740290-9740355:-	531
SLC9A4	chr2:102456566-102456650:+	5HSAR013962	chr2:102456245-102456322:+<br>chr2:102456362-102456475:+	457
FHL2	chr2:105369406-105381886:-	5HSAR023450	chr2:105379543-105381985:-	224
KCNF1	chr2:10969907-10970003:+	5HSAR001782	chr2:10969788-10969862:+	490
RGPD5	chr2:109909238-109909331:+	5HSAR029934	chr2:109907819-109909290:+	533
MALL	chr2:110230659-110230751:-	5HSAR016033	chr2:110231269-110231343:-<br>chr2:110230820-110230951:-	774
RGPD6	chr2:111051071-111051164:-	5HSAR037802	chr2:111051112-111052583:-	533
ANAPC1	chr2:112354874-112358066:-	5HSAR019195	chr2:112358009-112358116:-	247
CBWD2	chr2:113911814-113911915:+	5HSAR060058	chr2:113911805-113911891:+	178
ROCK2	chr2:11401714-11401808:-	5HSAR028666	chr2:11401999-11402142:-	449
SLC35F5	chr2:114230457-114230556:-	5HSAR003489	chr2:114230664-114230780:-	418
DPP10	chr2:114916735-114916825:+	5HSAR041728	chr2:114916435-114916539:+<br>chr2:114916592-114916672:+	457
GREB1	chr2:11614103-11614191:+	5HSAR026732	chr2:11591709-11591813:+	251
INSIG2	chr2:118570518-118570602:+	5HSAR028352	chr2:118562573-118570524:+	206
C1QL2	chr2:119632316-119632413:-	5HSAR010139	chr2:119632524-119632937:-	626
STEAP3	chr2:119697855-119719542:+	5HSAR014191	chr2:119704666-119704740:+	89
C2orf76	chr2:119814006-119814291:-	5HSAR011977	chr2:119840634-119840702:-<br>chr2:119840489-119840617:-	541
SCTR	chr2:119998273-119998365:-	5HSAR022079	chr2:119998340-119998480:-	226
TMEM177	chr2:120153260-120154899:+	5HSAR000984	chr2:120153245-120153313:+	132
PTPN4	chr2:120234367-120283899:+	5HSAR017490	chr2:120233821-120234078:+<br>chr2:120234259-120234327:+	771

CLASP1	chr2:122079942-122080038:-	5HSAR017142	chr2:122080139-122080225:- chr2:122079972-122080097:-	390
IWS1	chr2:128000255-128000332:-	5HSAR020943	chr2:128000376-128000483:-	303
GPR17	chr2:128122251-128124056:+	5HSAR040075	chr2:128120000-128120122:+ chr2:128122212-128122283:+	611
POTEF	chr2:130594559-130601766:-	5HSAR057601	chr2:130601790-130603218:- chr2:130594606-130601786:-	400
PLEKHB2	chr2:131579361-131594984:+	5HSAR001177	chr2:131578910-131578975:+ chr2:131579326-131594980:+	560
LYPD1	chr2:133143976-133144069:-	5HSAR010232	chr2:133144874-133144939:- chr2:133144314-133144580:-	976
ZRANB3	chr2:135978530-136005251:-	5HSAR002747	chr2:136005184-136005273:-	117
CXCR4	chr2:136589980-136590076:-	5HSAR030981	chr2:136590101-136590238:-	304
ARHGAP15	chr2:143603424-143629529:+	5HSAR024509	chr2:143603414-143603485:+	151
GTDC1	chr2:144682819-144685622:-	5HSAR040214	chr2:144685594-144709746:-	246
ORC4L	chr2:148432879-148494734:-	5HSAR049447	chr2:148494710-148494784:-	289
MBD5	chr2:148932719-148932797:+	5HSAR057062	chr2:148495079-148652778:+ chr2:148652793-148707366:+	997
RIF1	chr2:151975090-151975183:+	5HSAR058954	chr2:151974872-151975069:+	334
CACNB4	chr2:152538474-152538558:-	5HSAR026119	chr2:152538537-152538629:-	258
STAM2	chr2:152740403-152740496:-	5HSAR031611	chr2:152740456-152740632:-	350
FMNL2	chr2:152900256-152900363:+	5HSAR017186	chr2:152900088-152900168:+	367
ARL6IP6	chr2:153283286-153283384:+	5HSAR024354	chr2:153282843-153283052:+	716
TBR1	chr2:161981068-161981167:+	5HSAR009500	chr2:161980885-161980992:+	302
DPP4	chr2:162638738-162638831:-	5HSAR027285	chr2:162638842-162638976:-	561
KCNH7	chr2:163403275-163403366:-	5HSAR021000	chr2:163403341-163403415:-	212
CSRNP3	chr2:166082661-166159821:+	5HSAR055733	chr2:166034422-166059541:+ chr2:166059611-166059676:+	376
DHRS9	chr2:169634455-169646337:+	5HSAR016631	chr2:169631822-169634225:+ chr2:169634264-169634428:+	557

LRP2	chr2:169927156-169927244:-	5HSAR017363	chr2:169927235-169927309:-	213
FASTKD1	chr2:170136786-170136878:-	5HSAR011083	chr2:170138475-170138600:-<br>chr2:170136827-170136892:-	388
MYO3B	chr2:170742960-170743043:+	5HSAR015511	chr2:170742923-170743000:	143
OLA1	chr2:174714874-174714962:-	5HSAR030042	chr2:174796115-174802408:-<br>chr2:174796010-174796114:-	559
LOC100133109	chr2:175293224-175293300:-	5HSAR044507	chr2:175293240-175293320:-	132
CHN1	chr2:175577887-175577985:-	5HSAR022121	chr2:175577953-175578335:-	405
ATF2	chr2:175709418-175724087:-	5HSAR029184	chr2:175724085-175741044:-	262
VSNL1	chr2:17585580-17636822:+	5HSAR023473	chr2:17585433-17585519:+	384
HOXD8	chr2:176703249-176703340:+	5HSAR003719	chr2:176702738-176702821:+<br>chr2:176703131-176703214:+	618
PDE11A	chr2:178677437-178681309:-	5HSAR024537	chr2:178461639-178461704:-	99
FKBP7	chr2:179051473-179051559:-	5HSAR015293	chr2:179051508-179051594:-	129
ZNF385B	chr2:180318710-180318815:-	5HSAR027368	chr2:180318891-180318992:-	305
CWC22	chr2:180566314-180566409:-	5HSAR016704	chr2:180566340-180566423:-	300
ITGA4	chr2:182030550-182030626:+	5HSAR024803	chr2:182029884-182029949:+	763
SSFA2	chr2:182464937-182465038:+	5HSAR012110	chr2:182464831-182465019:+	322
ZNF804A	chr2:185171835-185171931:+	5HSAR013278	chr2:185171387-185171611:+	594
CALCRL	chr2:187958568-187960347:-	5HSAR027999	chr2:187960431-187960692:-	548
PMS1	chr2:190357483-190364780:+	5HSAR026414	chr2:190357133-190357507:+	529
STAT1	chr2:191582975-191586581:-	5HSAR030331	chr2:191586546-191586632:-	388
STAT4	chr2:191721175-191724004:-	5HSAR028119	chr2:191723964-191724029:-	254
TMEFF2	chr2:192767496-192767588:-	5HSAR027563	chr2:192767691-192767756:-<br>chr2:192767498-192767677:-	394
PLCL1	chr2:198377967-198378068:+	5HSAR058733	chr2:198377891-198377956:+	398

TTC32	chr2:19965097-19965183:-	5HSAR001722	chr2:19965148-19965228:-	132
SATB2	chr2:200029006-200030329:-	5HSAR032721	chr2:200030853-200030969:-	817
SPATS2L	chr2:200879566-200962212:+	5HSAR025736	chr2:200879271-200879513:+	412
SGOL2	chr2:201099127-201105943:+	5HSAR031916	chr2:201099118-201099213:+	113
LAPTM4A	chr2:20114763-20114861:-	5HSAR024863	chr2:20114939-20115181:-	508
CLK1	chr2:201437195-201437301:-	5HSAR049333	chr2:201437319-201437465:-	335
NDUFB3	chr2:201644941-201651850:+	5HSAR001390	chr2:201644710-201644826:+<br>chr2:201644829-201644921:+	323
CASP10	chr2:201756200-201758745:+	5HSAR024913	chr2:201755872-201755982:+<br>chr2:201756061-201756207:+	418
STRADB	chr2:202024901-202027808:+	5HSAR013643	chr2:202024704-202027768:+	365
ALS2	chr2:202341854-202353876:-	5HSAR027921	chr2:202341882-202353926:-	356
WDR12	chr2:203484445-203484540:-	5HSAR024813	chr2:203484884-203485060:-<br>chr2:203484682-203484756:-	750
ALS2CR8	chr2:203497334-203514870:+	5HSAR029508	chr2:203485585-203485662:+<br>chr2:203485743-203485826:+	1038
INO80D	chr2:206635986-206636179:-]	5HSAR012691	chr2:206636011-206659038:-	405
FASTKD2	chr2:207338578-207339662:+	5HSAR017926	chr2:207338373-207338534:+	317
PLEKHM3	chr2:208574609-208574687:-	5HSAR016450	chr2:208598372-208598476:-<br>chr2:208574704-208574814:-	490
C2orf80	chr2:208759956-208762933:-	5HSAR012124	chr2:208759965-208763014:-	172
IKZF2	chr2:213721599-213723187:-	5HSAR010004	chr2:213724436-213724558:-<br>chr2:213723146-213724412:-	316
MREG	chr2:216586296-216586381:-	5HSAR000343	chr2:216586438-216586572:-	296
TMEM169	chr2:216668843-216668931:+	5HSAR016361	chr2:216655186-216668862:+	326
BCS1L	chr2:219233174-219233954:+	5HSAR017325	chr2:219233020-219233118:+	346
STK36	chr2:219245178-219245796:+	5HSAR016659	chr2:219245064-219245153:+	266
TTLL4	chr2:219310555-219310643:+	5HSAR016655	chr2:219310563-219310631:+	370

CRYBA2	chr2:219566143-219566252:-	5HSAR021415	chr2:219566184-219566336:-	229
GMPPA	chr2:220072171-220072941:+	5HSAR015471	chr2:220072049-220072153:+	375
MFF	chr2:227902616-227902705:+	5HSAR017211	chr2:227901636-227901716:+	266
SP110	chr2:230794681-230798512:-	5HSAR055763	chr2:230794738-230798625:-	258
ATG16L1	chr2:233825122-233825212:+	5HSAR030184	chr2:233825048-233825191:+	257
SAG	chr2:233881360-233882574:+	5HSAR029950	chr2:233881086-233881202:+<br>chr2:233881210-233881353:+	388
ARL4C	chr2:235069970-235070079:-	5HSAR010532	chr2:235070031-235070183:-	463
SH3BP4	chr2:235608278-235608385:+	5HSAR002160	chr2:235525543-235608357:+	393
AGAP1	chr2:236067967-236068069:+	5HSAR013665	chr2:236067695-236067889:+	595
COPS8	chr2:237659256-237659346:+	5HSAR012163	chr2:237658907-237659002:+<br>chr2:237659035-237659112:+	524
HDAC4	chr2:239939332-239939422:-	5HSAR025357	chr2:239939464-239987376:-<br>chr2:239939352-239939426:-	792
ANKMY1	chr2:241143025-241143117:-	5HSAR002493	chr2:241143078-241143143:-	229
C2orf44	chr2:24115869-24123762:-	5HSAR016369	chr2:24115883-24123755:-	131
RNPEPL1	chr2:241160593-241161223:+	5HSAR003321	chr2:241160541-241160627:+	593
HDLBP	chr2:241854958-241856628:-	5HSAR013392	chr2:241856612-241903879:-	372
NEU4	chr2:242401117-242401196:+	5HSAR058171	chr2:242400706-242400975:+	494
EFR3B	chr2:25118601-25118685:+	5HSAR011566	chr2:25118499-25118609:+	209
FAM59B	chr2:26249502-26249593:+	5HSAR056266	chr2:26257178-26257273:+<br>chr2:26257509-26257577:+	130
C2orf53	chr2:27214702-27214794:-	5HSAR000633	chr2:27215743-27215823:-<br>chr2:27214954-27215061:-	580
DNAJC5G	chr2:27352654-27353100:+	5HSAR002058	chr2:27351887-27352576:+	418
YPEL5	chr2:30224845-30233021:+	5HSAR011070	chr2:30224662-30224727:+<br>chr2:30224844-30233019:+	500
EHD3	chr2:31310898-31310991:+	5HSAR034741	chr2:31310388-31310498:+<br>chr2:31310520-31310714:+	608

NLRC4	chr2:32335349-32335451:-	5HSAR011636	chr2:32335376-32335465:-	264
YIPF4	chr2:32356634-32356728:+	5HSAR025007	chr2:32356486-32356554:+	267
RASGRP3	chr2:33593617-33593707:+	5HSAR024255	chr2:33514945-33515010:+ chr2:33515020-33526454:+	627
TSSC1	chr2:3360468-3360557:-	5HSAR016802	chr2:3360564-3360659:- chr2	193
VIT	chr2:36777551-36797017:+	5HSAR041406	chr2:36777369-36777461:+ chr2:36777526-36777594:+	302
FAM82A1	chr2:38031140-38031225:+	5HSAR041954	chr2:38031100-38031174:+	126
GALM	chr2:38746717-38746807:+	5HSAR022655	chr2:38746622-38746732:+	252
SRSF7	chr2:38831903-38832003:-	5HSAR022322	chr2:38831929-38832066:-	238
GEMIN6	chr2:38858852-38859636:+	5HSAR010940	chr2:38858841-38858924:+	116
PPM1B	chr2:44249845-44289867:+	5HSAR024028	chr2:44249546-44249632:+ chr2:44249657-44249776:+	429
PREPL	chr2:44440359-44442213:-	5HSAR059491	chr2:44442324-44442407:-	374
SIX2	chr2:45089754-45089845:-	5HSAR018396	chr2:45089899-45090018:-	293
EPAS1	chr2:46378461-46378554:+	5HSAR048863	chr2:46378151-46378297:+	510
FOXN2	chr2:48409264-48426857:+	5HSAR019554	chr2:48395303-48395455:+ chr2:48409216-48409290:+	315
GPR75	chr2:53935398-53935491:-	5HSAR011066	chr2:53935456-53940588:-	271
RTN4	chr2:55090761-55090846:-	5HSAR026059	chr2:55090767-55090850:-	214
C2orf63	chr2:55303052-55308695:-	5HSAR014404	chr2:55308840-55312950:- chr2:55308720-55308836:-	346
EFEMP1	chr2:56003080-56003629:-	5HSAR026274	chr2:56003616-56003741:-	330
CCDC85A	chr2:56265170-56265263:+	5HSAR000284	chr2:56264996-56265094:+	502
C2orf86	chr2:63668910-63668991:-	5HSAR022604	chr2:63669243-63669332:- chr2:63669150-63669224:-	446
CEP68	chr2:65137131-65150082:+	5HSAR013808	chr2:65137116-65150069:+	214
NFU1	chr2:69512632-69517971:-	5HSAR023311	chr2:69517993-69518058:-	361

C2orf42	chr2:70262622-70263381:-	5HSAR000005	chr2:70263412-70263477:-<br>chr2:70262625-70263404:-	328
CCT7	chr2:73314915-73315014:+	5HSAR053871	chr2:73315015-73316281:+	143
NAT8	chr2:73722264-73722969:-	5HSAR022595	chr2:73722308-73723012:-	167
CCDC142	chr2:74563473-74563566:-	5HSAR016687	chr2:74563638-74563709:-<br>chr2:74563496-74563600:-	393
VPS24	chr2:86643983-86644055:-	5HSAR013352	chr2:86643996-86644109:-	149
KRCC1	chr2:88109198-88114829:-	5HSAR000129	chr2:88117578-88117673:-<br>chr2:88114834-88114926:-	394
KIDINS220	chr2:8884675-8895116:-	5HSAR017235	chr2:8895108-8895173:-	182
ZNF2	chr2:95195332-95207047:+	5HSAR012638	chr2:95194966-95195031:+<br>chr2:95195207-95195290:+	516
LOC100127913	chr2:9613856-9613953:-	5HSAR004460	chr2:9614787-9614903:-<br>chr2:9614687-9614779:-	1078
ITPRIPL1	chr2:96355974-96356072:+	5HSAR058840	chr2:96355754-96355900:+	411
INPP4A	chr2:98502851-98502943:+	5HSAR025694	chr2:98501859-98502865:+	393
MGAT4A	chr2:98709228-98709315:-	5HSAR014711	chr2:98709258-98709359:-	314
TSGA10	chr2:99092335-99093754:-	5HSAR011053	chr2:99109981-99110055:-<br>chr2:99101462-99101533:-	652
LIPT1	chr2:99141233-99144852:+	5HSAR009796	chr2:99137888-99139289:+	340
MRPL30	chr2:99164078-99169098:+	5HSAR009292	chr2:99164022-99164135:+	162
TXNDC9	chr2:99316106-99319195:-	5HSAR020485	chr2:99319176-99319244:-	191
DSTN	chr20:17520672-17529430:+	5HSAR003839	chr20:17498854-17520666:+	438
BANF2	chr20:17651420-17653670:+	5HSAR029920	chr20:17628603-17651361:+	274
ZNF133	chr20:18233728-18234330:+	5HSAR019641	chr20:18226704-18233673:+	559
SEC23B	chr20:18436538-18439479:+	5HSAR025668	chr20:18436258-18436446:+<br>chr20:18436512-18436616:+	443
C20orf26	chr20:19981207-19985335:+	5HSAR040524	chr20:19981205-19985332:+	114
LOC100131342	chr20:20293653-20293740:-	5HSAR008728	chr20:20295065-20295133:-<br>chr20:20294907-20294981:-	1491

PTPRA	chr20:2851899-2892923:+	5HSAR019162	chr20:2792876-2851856:+	675
DEFB121	chr20:29464108-29464199:-	5HSAR033032	chr20:29457630-29457722:-	195
DUSP15	chr20:29921802-29921897:-	5HSAR017085	chr20:29921860-29922051:-	339
TRIB3	chr20:309720-309813:+	5HSAR026872	chr20:309389-309682:+	506
CDK5RAP1	chr20:31448262-31448352:-	5HSAR003196	chr20:31448324-31452982:-	246
CBFA2T2	chr20:31541648-31541734:+	5HSAR027945	chr20:31613941-31614006:+	146
C20orf134	chr20:31718863-31718964:+	5HSAR001824	chr20:31718030-31718104:+<br>chr20:31718233-31718304:+	1000
TP53INP2	chr20:32756887-32760204:+	5HSAR028837	chr20:32755889-32756886:+	335
NCOA6	chr20:32833820-32843958:-	5HSAR019743	chr20:32833868-32843978:-	322
MYH7B	chr20:33009676-33026958:+	5HSAR054275	chr20:33007370-33009615:+	317
DLGAP4	chr20:34429068-34493534:+	5HSAR034742	chr20:34428994-34493504:+	307
DSN1	chr20:34832997-34835553:-	5HSAR021863	chr20:34835562-34835633:-	180
SRC	chr20:35444525-35445970:+	5HSAR029671	chr20:35406684-35427068:+<br>chr20:35444436-35444528:+	449
BLCAP	chr20:35580991-35581079:-	5HSAR040643	chr20:35581091-35586224:-	351
C20orf27	chr20:3688748-3696317:-	5HSAR000517	chr20:3688796-3696451:-	229
CDC25B	chr20:3725088-3725178:+	5HSAR028434	chr20:3724502-3724618:+<br>chr20:3724639-3724872:+	778
CHD6	chr20:39613391-39680472:-	5HSAR014294	chr20:39680473-39680547:-	178
JPH2	chr20:42248760-42248851:-	5HSAR022333	chr20:42249378-42249527:-<br>chr20:42249142-42249327:-	873
PCIF1	chr20:43999558-44001045:+	5HSAR010012	chr20:43996754-43999510:+	314
CDH22	chr20:44313341-44313428:-	5HSAR012027	chr20:44313442-44313732:-	401
PRNP	chr20:4615076-4627866:+	5HSAR029687	chr20:4614820-4614951:+	367
CSE1L	chr20:47096283-47108407:+	5HSAR013076	chr20:47096281-47108405:+	134

UBE2V1	chr20:48165520-48165693:-	5HSAR003184	chr20:48165800-48165880:-<br>chr20:48165532-48165707:-	305
ZNF217	chr20:51632773-51632852:-	5HSAR017517	chr20:51632955-51633029:-	271
PFDN4	chr20:52257951-52258046:+	5HSAR003630	chr20:52257927-52258034:+	138
CSTF1	chr20:54400938-54404015:+	5HSAR021218	chr20:54401011-54401109:+	200
TFAP2C	chr20:54637919-54638007:+	5HSAR025958	chr20:54637856-54637927:+	243
RAE1	chr20:55360306-55362501:+	5HSAR011711	chr20:55360047-55360130:+<br>chr20:55360188-55360322:+	365
PMEPA1	chr20:55718045-55718147:-	5HSAR017473	chr20:55718214-55718414:-	393
TUBB1	chr20:57027878-57027972:+	5HSAR026823	chr20:57027752-57027925:+	269
CDH26	chr20:57967082-57967176:+	5HSAR057213	chr20:57966901-57967011:+<br>chr20:57967027-57967113:+	311
CRLS1	chr20:5934798-5934892:+	5HSAR026404	chr20:5934772-5934852:+	154
HRH3	chr20:60228422-60228514:-	5HSAR029459	chr20:60228434-60228562:-	297
OSBPL2	chr20:60264542-60264635:+	5HSAR001888	chr20:60264567-60264632:+	202
FERMT1	chr20:6048202-6051501:-	5HSAR028055	chr20:6051889-6051999:-	789
DIDO1	chr20:61013410-61016043:-	5HSAR030543	chr20:61016114-61016179:-	319
KCNQ2	chr20:61574261-61574360:-	5HSAR017292	chr20:61574298-61574363:-	177
RTEL1	chr20:61761121-61761199:+	5HSAR017086	chr20:61761080-61761154:+	343
TNFRSF6B	chr20:61798479-61798564:+	5HSAR024837	chr20:61796871-61797582:+	100
TCEA2	chr20:62159439-62168281:+	5HSAR002795	chr20:62158971-62159351:+	649
OPRL1	chr20:62193844-62194517:+	5HSAR018909	chr20:62181990-62193866:+	402
ANKRD5	chr20:9963924-9966949:+	5HSAR002519	chr20:9963752-9963937:+	329
ATP5J	chr21:26029138-26029215:-	5HSAR029452	chr21:26029167-26029352:-	699
USP16	chr21:29318915-29322105:+	5HSAR015132	chr21:29318824-29322089:+	202

C21orf7	chr21:29379309-29380052:+	5HSAR014150	chr21:29374744-29374887:+	277
GRIK1	chr21:30233690-30233778:-	5HSAR028733	chr21:30233911-30234036:-<br>chr21:30233702-30233776:-	464
TIAM1	chr21:31561160-31570989:-	5HSAR029738	chr21:31633589-31758215:-<br>chr21:31571065-31633532:-	472
IFNGR2	chr21:33697624-33697719:+	5HSAR024768	chr21:33697184-33697654:+	648
DNAJC28	chr21:33783571-33785302:-	5HSAR011634	chr21:33785499-33785654:-<br>chr21:33785315-33785389:-	449
GART	chr21:33833492-33836887:-	5HSAR030418	chr21:33836963-33837037:-	274
SLC5A3	chr21:34389290-34389367:+	5HSAR026410	chr21:34367871-34389055:+<br>chr21:34389098-34389265:+	512
RCAN1	chr21:34820918-34821002:-	5HSAR028788	chr21:34821015-34821116:-	214
RUNX1	chr21:35181280-35181357:-	5HSAR028083	chr21:35182481-35182627:-<br>chr21:35182153-35182296:-	1578
CLDN14	chr21:36755864-36760536:-	5HSAR024220	chr21:36755879-36870647:-<br>chr21:36774087-36774194:-	142
HLCS	chr21:37233054-37233146:-	5HSAR059774	chr21:37240210-37240302:-	471
B3GALT5	chr21:39951604-39951690:+	5HSAR003742	chr21:39951154-39951348:+	382
TMPRSS3	chr21:42688596-42689163:-	5HSAR022935	chr21:42688645-42689225:-	201
RSPH1	chr21:42789366-42789461:-	5HSAR013075	chr21:42789385-42789450:-	105
SLC37A1	chr21:42811554-42811633:+	5HSAR001517	chr21:42795510-42811587:+	412
AIRE	chr21:44535028-44535117:+	5HSAR027889	chr21:44534712-44534837:+	416
UBE2G2	chr21:45032304-45045674:-	5HSAR015060	chr21:45045701-45046091:-<br>chr21:45032406-45045668:-	274
PTTG1IP	chr21:45117960-45118045:-	5HSAR001928	chr21:45117993-45118118:-	210
DIP2A	chr21:46703378-46703472:+	5HSAR025224	chr21:46703369-46703461:+	183
BID	chr22:16602190-16606595:-	5HSAR024781	chr22:16606575-16606754:-	388
PEX26	chr22:16940800-16941142:+	5HSAR024806	chr22:16940699-16941114:+	209
USP18	chr22:17020340-17020430:+	5HSAR014255	chr22:17012978-17020384:+	338

HIRA	chr22:17799000-17799091:-	5HSAR020864	chr22:17799126-17799218:-	220
GNB1L	chr22:18188879-18222033:-	5HSAR017559	chr22:18222372-18222461:-	237
DGCR8	chr22:18453380-18453486:+	5HSAR040526	chr22:18447826-18453287:+<br>chr22:18453331-18453420:+	429
RIMBP3	chr22:18841302-18841394:-	5HSAR024262	chr22:18841584-18841742:-<br>chr22:18841346-18841531:-	485
RIMBP3C	chr22:20235266-20235358:-	5HSAR024228	chr22:20235548-20235706:-<br>chr22:20235310-20235495:-	485
YPEL1	chr22:20395034-20395119:-	5HSAR003142	chr22:20419977-20420042:-<br>chr22:20395054-20419942:-	332
PRAMEL	chr22:20728147-20728235:-	5HSAR045685	chr22:20728163-20728243:-	110
MYO18B	chr22:24486966-24487059:+	5HSAR017668	chr22:24468128-24468220:+	250
THOC5	chr22:28275137-28279733:-	5HSAR029043	chr22:28279661-28279732:-	88
NF2	chr22:28329887-28329987:+	5HSAR027230	chr22:28329607-28329675:+<br>chr22:28329831-28329932:+	443
RNF185	chr22:29886249-29913080:+	5HSAR014627	chr22:29886168-29886260:+	200
LIMK2	chr22:29974612-29974702:+	5HSAR019895	chr22:29974395-29974466:+<br>chr22:29974492-29974572:+	355
RFPL2	chr22:30919262-30919357:-	5HSAR029778	chr22:30919377-30919463:-	205
RFPL3	chr22:31083957-31084058:+	5HSAR012871	chr22:31083897-31083971:+	205
MB	chr22:34343251-34349257:-	5HSAR019978	chr22:34349242-34349343:-	172
GGA1	chr22:36334706-36334813:+	5HSAR033121	chr22:36334455-36334583:+	387
H1F0	chr22:36531397-36531497:+	5HSAR019769	chr22:36531073-36531396:+	438
C22orf23	chr22:36679103-36679378:-	5HSAR001658	chr22:36679397-36679480:-	259
MAFF	chr22:36928112-36939806:+	5HSAR057754	chr22:36927905-36928081:+	312
MGAT3	chr22:38183406-38213298:+	5HSAR014744	chr22:38183357-38183491:+	239
SMCR7L	chr22:38230392-38237245:+	5HSAR013980	chr22:38228313-38230158:+<br>chr22:38230182-38230394:+	498
PACSIN2	chr22:41638031-41672855:-	5HSAR012182	chr22:41638064-41672940:-	222

TSPO	chr22:41877496-41885188:+	5HSAR021513	chr22:41877487-41885160:+	105
EFCAB6	chr22:42509532-42536299:-	5HSAR039219	chr22:42536283-42539468:-	314
PARVG	chr22:42909042-42910542:+	5HSAR025064	chr22:42908124-42908979:+	484
PRR5	chr22:43451702-43477045:+	5HSAR022862	chr22:43451381-43451500:+<br>chr22:43451514-43451579:+	443
NUP50	chr22:43945644-43946159:+	5HSAR017257	chr22:43942723-43945556:+<br>chr22:43945606-43945713:+	372
GTSE1	chr22:45071427-45071961:+	5HSAR058305	chr22:45071326-45071451:+	212
GTF3C4	chr22:45071427-45071961:+	5HSAR015433	chr22:45071326-45071451:+	258
ZBED4	chr22:48663233-48663314:+	5HSAR018601	chr22:48633571-48662989:+<br>chr22:48663008-48663097:+	470
FILIP1L	chr3:101132555-101315680:-	5HSAR025106	chr3:101315893-101315979:-<br>chr3:101132558-101315861:-	463
TFG	chr3:101911210-101915219:+	5HSAR003648	chr3:101910955-101911071:+	449
IFT57	chr3:109423860-109423961:-	5HSAR017634	chr3:109423991-109424107:-	248
CNTN6	chr3:1109802-1164692:+	5HSAR019325	chr3:1109738-1164618:+	267
ZBED2	chr3:112795739-112795814:-	5HSAR002372	chr3:112796426-112796578:-<br>chr3:112796137-112796205:-	885
BOC	chr3:114417809-114451339:+	5HSAR002146	chr3:114414105-114414182:+<br>chr3:114417749-114451293:+	339
SIDT1	chr3:114734452-114734558:+	5HSAR001952	chr3:114734122-114734283:+	651
DRD3	chr3:115373530-115380239:-	5HSAR028756	chr3:115380413-115380538:-<br>chr3:115380314-115380409:-	431
LSAMP	chr3:117646569-117646674:-	5HSAR055386	chr3:117646638-117646766:-	507
TMEM39A	chr3:120663612-120664895:-	5HSAR003672	chr3:120665053-120665139:-	363
CD80	chr3:120759266-120759356:-	5HSAR027128	chr3:120759284-120761115:-	395
TIMP4	chr3:12175341-12175425:-	5HSAR027429	chr3:12175495-12175605:-	307
WDR5B	chr3:123617066-123617170:-	5HSAR002577	chr3:123617273-123617356:-<br>chr3:123617173-123617265:-	507
PARP9	chr3:123761158-123761246:-	5HSAR026664	chr3:123761189-123765658:-	326

ITGB5	chr3:126088538-126088629:-	5HSAR026864	chr3:126088553-126088645:-	297
TPRA1	chr3:128781680-128781756:-	5HSAR016759	chr3:128781857-128781961:-	291
TMCC1	chr3:131029912-131030007:-	5HSAR054236	chr3:131034312-131082045:-	434
COL6A5	chr3:131547445-131575171:+	5HSAR012104	chr3:131547051-131547134:+	494
ACAD11	chr3:133861286-133861378:-	5HSAR003090	chr3:133861478-133861576:-	380
SRPRB	chr3:135007294-135007382:+	5HSAR035881	chr3:134985574-134985666:+	285
FBLN2	chr3:13585538-13586855:+	5HSAR048866	chr3:13585262-13585384:+	382
EPHB1	chr3:135997062-135997163:+	5HSAR054280	chr3:135996837-135996938:+	375
PPP2R3A	chr3:137202945-137203030:+	5HSAR054025	chr3:137202614-137202682:+<br>chr3:137202693-137202776:+	617
MSL2	chr3:137396646-137396738:-	5HSAR022388	chr3:137397184-137397255:-<br>chr3:137396966-137397118:-	733
STAG1	chr3:137832431-137953740:-	5HSAR012037	chr3:137953791-137953895:-	292
ARMC8	chr3:139389972-139390059:+	5HSAR003173	chr3:139389087-139389998:+	421
CEP70	chr3:139774460-139793464:-	5HSAR003563	chr3:139793414-139793488:-	198
TRIM42	chr3:141879691-141879761:+	5HSAR020312	chr3:141879581-141879667:+<br>chr3:141879679-141879761:+	206
SPSB4	chr3:142267538-142267636:+	5HSAR002757	chr3:142253522-142267600:+	254
PLS1	chr3:143858655-143865769:+	5HSAR050841	chr3:143858530-143858616:+	320
CHST2	chr3:144322273-144322348:+	5HSAR013125	chr3:144321595-144321732:+	889
ZIC1	chr3:148610499-148610589:+	5HSAR023968	chr3:148609953-148610117:+<br>chr3:148610196-148610294:+	719
FLJ30375	chr3:148621378-148621475:+	5HSAR044573	chr3:148620381-148620464:+<br>chr3:148620615-148620734:+	1255
AGTR1	chr3:149940386-149941512:+]	5HSAR030555	chr3:149930657-149940374:+	262
GYG1	chr3:150192025-150192117:+	5HSAR038916	chr3:150191945-150192070:+	233
WWTR1	chr3:150857784-150870346:-	5HSAR038188	chr3:150870297-150903633:-	257

RNF13	chr3:151013752-151046503:+	5HSAR031271	chr3:151013167-151013568:+<br>chr3:151013621-151013716:+	682
ZFYVE20	chr3:15112632-15112721:-	5HSAR002010	chr3:15114740-15115643:-<br>chr3:15113183-15114692:-	614
SH3BP5	chr3:15286248-15286337:-	5HSAR010357	chr3:15357755-15357889:-<br>chr3:15320802-15357748:-	573
MBNL1	chr3:153500587-153500672:+	5HSAR021531	chr3:153468545-153468661:+<br>chr3:153468667-153468885:+	1842
METTL6	chr3:15443023-15443110:-	5HSAR012838	chr3:15443098-15444025:-	258
COLQ	chr3:15515099-15515202:-	5HSAR021758	chr3:15515136-15515324:-	285
MME	chr3:156280157-156284650:+	5HSAR030487	chr3:156280159-156280230:+	120
PLCH1	chr3:156876523-156876602:-	5HSAR015182	chr3:156876693-156876797:-<br>chr3:156876572-156876682:-	277
KCNAB1	chr3:157343570-157343661:+	5HSAR021459	chr3:157343445-157343534:+	217
TIPARP	chr3:157875829-157878180:+	5HSAR042683	chr3:157875459-157878155:+	507
CCNL1	chr3:158360578-158360664:-	5HSAR011093	chr3:158360903-158360974:-<br>chr3:158360686-158360775:-	599
MLF1	chr3:159771808-159792944:+	5HSAR024453	chr3:159771784-159771873:+	256
IFT80	chr3:161585083-161599674:-	5HSAR013349	chr3:161599660-161600010:-	433
DAZL	chr3:16621014-16621116:-	5HSAR056162	chr3:16621095-16621238:-	288
ZBBX	chr3:168573384-168574230:-	5HSAR010374	chr3:168574224-168580250:-<br>chr3:168573386-168574212:-	323
MECOM	chr3:170331960-170344223:-	5HSAR037993	chr3:170344188-170344295:-	269
MYNN	chr3:170973885-170974777:+	5HSAR014101	chr3:170973636-170973839:+	429
NCEH1	chr3:173846145-173911378:-	5HSAR027674	chr3:173911561-173911632:-	404
TBC1D5	chr3:17525067-17578573:-	5HSAR021655	chr3:17525098-17578636:-	427
KCNMB3	chr3:180467193-180467273:-	5HSAR022917	chr3:180467427-180467501:-	340
ZNF639	chr3:180525788-180528775:+	5HSAR003063	chr3:180524277-180525563:+<br>chr3:180525614-180525724:+	445
SATB1	chr3:18437464-18440194:-	5HSAR023210	chr3:18441710-18441799:-<br>chr3:18441533-18441697:-	1735

ABCC5	chr3:185214875-185218347:-	5HSAR016945	chr3:185214921-185218401:-	165
HTR3D	chr3:185235647-185236881:+	5HSAR020642	chr3:185235568-185235681:+	225
HTR3E	chr3:185300762-185300854:+	5HSAR025409	chr3:185300748-185300813:+	194
EIF2B5	chr3:185335783-185335867:+	5HSAR017849	chr3:185335512-185335586:+ chr3:185335622-185335777:+	364
EIF4G1	chr3:185516263-185516278:+	5HSAR031395	chr3:185515979-185516278:+	271
EHHADH	chr3:186435835-186435921:-	5HSAR042261	chr3:186454427-186454504:-	487
BCL6	chr3:188934176-188936622:-	5HSAR030083	chr3:188936828-188936911:- chr3:188935383-188936690:-	457
UTS2D	chr3:192482673-192482752:-	5HSAR014639	chr3:192516879-192529159:- chr3:192516625-192516804:-	787
CCDC50	chr3:192530063-192530157:+	5HSAR019296	chr3:192529577-192530113:+	590
ATP13A3	chr3:195664209-195669948:-	5HSAR025309	chr3:195670149-195670223:- chr3:195669962-195670084:-	402
FAM43A	chr3:195888749-195888844:+	5HSAR000358	chr3:195888022-195888111:+ chr3:195888152-195888244:+	934
MUC4	chr3:197023086-197023176:-	5HSAR025729	chr3:197023290-197023379:-	460
TNK2	chr3:197099857-197119814:-	5HSAR015725	chr3:197119788-197119913:-	543
RNF168	chr3:197714442-197714476:-	5HSAR024073	chr3:197714760-197714825:- chr3:197714609-197714677:-	595
WDR53	chr3:197772744-197778220:-	5HSAR020444	chr3:197778384-197778461:- chr3:197778209-197778373:-	471
DLG1	chr3:198508473-198509749:-	5HSAR029398	chr3:198508485-198509801:-	190
BDH1	chr3:198757712-198765858:-	5HSAR020622	chr3:198765861-198765947:-	402
KIAA0226	chr3:198929284-198929363:-	5HSAR023610	chr3:198960769-198960936:- chr3:198929367-198960639:-	544
FYTTD1	chr3:198961148-198961239:+	5HSAR032960	chr3:198966956-198967033:+	219
THRB	chr3:24245455-24313771:-	5HSAR025226	chr3:24313773-24353814:-	349
RARB	chr3:25477783-25517706:+	5HSAR029187	chr3:25444860-25477694:+	448
CNTN4	chr3:3056697-3056789:+	5HSAR021354	chr3:3056325-3056561:+ chr3:3056613-3056690:+	482

IL5RA	chr3:3121669-3125266:-	5HSAR024522	chr3:3126971-3127045:-<br>chr3:3126762-3126938:-	644
OSBPL10	chr3:31997676-31997771:-	5HSAR048459	chr3:31997960-31998091:-	671
UBP1	chr3:33456345-33456432:-	5HSAR011234	chr3:33456597-33456680:-	530
ARPP21	chr3:35697619-35698247:+	5HSAR015981	chr3:35697612-35698232:+	272
MLH1	chr3:37009956-37010042:+	5HSAR037905	chr3:37009932-37010006:+	198
ACAA1	chr3:38153531-38153632:-	5HSAR022386	chr3:38153539-38153619:-	207
LRRN1	chr3:3861234-3861325:+	5HSAR017528	chr3:3861084-3861158:+	761
SCN11A	chr3:38966858-38966954:-	5HSAR003531	chr3:38966866-38966961:-	199
SLC25A38	chr3:39400132-39400219:+	5HSAR009598	chr3:39399945-39400043:+	401
MYRIP	chr3:39826341-39917311:+	5HSAR012521	chr3:39826371-39917309:+	135
TRAK1	chr3:42176756-42176857:+	5HSAR021666	chr3:42176670-42176825:+	193
ZBTB47	chr3:42670366-42674851:+	5HSAR058367	chr3:42670301-42674781:+	281
C3orf39	chr3:43097928-43098018:-	5HSAR001719	chr3:43097948-43098013:-	343
SNRK	chr3:43319607-43319699:+	5HSAR030072	chr3:43303082-43316279:+<br>chr3:43319600-43319665:+	332
ABHD5	chr3:43707402-43707488:+	5HSAR017584	chr3:43707382-43707462:+	110
ZNF445	chr3:44472046-44472141:-	5HSAR003214	chr3:44474752-44474826:-<br>chr3:44472092-44472190:-	348
ITPR1	chr3:4510289-4533175:+	5HSAR044080	chr3:4510041-4511180:+	350
ALS2CL	chr3:46693205-46693406:-	5HSAR016166	chr3:46693370-46693868:-	432
TMIE	chr3:46717891-46717981:+	5HSAR023598	chr3:46717853-46717927:+	155
KIF9	chr3:47293869-47299067:-	5HSAR013284	chr3:47299213-47299281:-<br>chr3:47293870-47299086:-	358
SLC26A6	chr3:48646033-48646118:-	5HSAR023428	chr3:48646157-48646228:-	251
IP6K2	chr3:48727787-48727868:-	5HSAR040351	chr3:48727814-48727936:-	300

WDR6	chr3:49019688-49019778:+	5HSAR013196	chr3:49019663-49019767:+	138
DALRD3	chr3:49030267-49030520:-	5HSAR003547	chr3:49030518-49030736:-	525
NDUFAF3	chr3:49034494-49034581:+	5HSAR024045	chr3:49034348-49034428:+	504
CCDC71	chr3:49176646-49178730:-	5HSAR000653	chr3:49176650-49178760:-	138
KLHDC8B	chr3:49185159-49185206:+	5HSAR000164	chr3:49185180-49185206:+	235
C3orf62	chr3:49289310-49289398:-	5HSAR013113	chr3:49289357-49289473:-	203
RHOA	chr3:49388027-49424344:-	5HSAR030958	chr3:49424359-49424526:-	276
DAG1	chr3:49522874-49522971:+	5HSAR038700	chr3:49481447-49522896:+	459
HYAL1	chr3:50314846-50314938:-	5HSAR022111	chr3:50314910-50314996:-	321
HYAL2	chr3:50332925-50333864:-	5HSAR053281	chr3:50335159-50335242:-<br>chr3:50332964-50334075:-	652
TMEM115	chr3:50371499-50371588:-	5HSAR022465	chr3:50371564-50371821:-	445
C3orf18	chr3:50578135-50578222:-	5HSAR053479	chr3:50579921-50580076:-	492
CISH	chr3:50622879-50622964:-	5HSAR023026	chr3:50622907-50624085:-	287
PCBP4	chr3:51970297-51971028:-	5HSAR043731	chr3:51970360-51976487:-	353
POC1A	chr3:52159033-52160145:-	5HSAR051817	chr3:52160174-52163428:-	503
ALAS1	chr3:52207846-52208297:+	5HSAR020497	chr3:52207730-52207882:+	337
NT5DC2	chr3:52543710-52543795:-	5HSAR012893	chr3:52543855-52544118:-	424
C3orf63	chr3:56670058-56671515:-	5HSAR000943	chr3:56672990-56673079:-<br>chr3:56672855-56672989:-	640
SPATA12	chr3:57082663-57082762:+	5HSAR000895	chr3:57069572-57069670:+<br>chr3:57069702-57069794:+	675
SLMAP	chr3:57718322-57718418:+	5HSAR001977	chr3:57718227-57718334:+	205
C3orf14	chr3:62280479-62281186:+	5HSAR000154	chr3:62280468-62280542:+	124
SYNPR	chr3:63404068-63404161:+	5HSAR013955	chr3:63403847-63403945:+	369

PRICKLE2	chr3:64159644-64185676:-	5HSAR014233	chr3:64185942-64186016:-<br>chr3:64185686-64185835:-	586
MAGI1	chr3:65999024-65999127:-	5HSAR017202	chr3:65999196-65999261:-<br>chr3:65999030-65999119:-	526
FAM19A1	chr3:68136445-68138459:+	5HSAR014041	chr3:68136149-68136253:+<br>chr3:68136263-68136334:+	395
C3orf64	chr3:69141688-69144370:-	5HSAR002428	chr3:69143798-69144449:-	405
FOXP1	chr3:71330223-71491017:-	5HSAR021202	chr3:71625285-71713457:-<br>chr3:71431710-71491053:-	526
EBLN2	chr3:73193843-73193922:+	5HSAR000132	chr3:73193604-73193687:+<br>chr3:73193723-73193863:+	423
C3orf32	chr3:8650625-8652446:-	5HSAR000695	5'chr3:8652465-8668683:-	246
VGLL3	chr3:87122584-87122680:-	5HSAR010338	chr3:87122626-87122877:-	364
CGGBP1	chr3:88187817-88189107:-	5HSAR014114	chr3:88189979-88190056:-<br>chr3:88189863-88189931:-	512
ARL13B	chr3:95181868-95181957:+	5HSAR059767	chr3:95181958-95205202:+<br>chr3:95205205-95205291:+	285
LHFPL4	chr3:9569364-9569439:-	5HSAR000342	chr3:9570410-9570481:-	286
CIDEC	chr3:9895171-9895696:-	5HSAR013605	chr3:9895642-9895737:-	140
ARL6	chr3:98968168-98969641:+	5HSAR029336	chr3:98966313-98966414:+	313
MINA	chr3:99169128-99173503:-	5HSAR019812	chr3:99173484-99173630:-	506
CRELD1	chr3:9951051-9951122:+	5HSAR058505	chr3:9950894-9951001:+	599
CLDND1	chr3:99722959-99724146:-	5HSAR003536	chr3:99724415-99724489:-<br>chr3:99724147-99724389:-	543
ZNF518B	chr4:10057051-10057139:-	5HSAR002134	chr4:10065600-10065671:-<br>chr4:10057180-10065582:-	487
MTTP	chr4:100715012-100715089:+	5HSAR030646	chr4:100704276-100715026:+	256
EMCN	chr4:101658095-101658182:-	5HSAR055075	chr4:101658112-101658219:-	179
PPP3CA	chr4:102486977-102487063:-	5HSAR025396	chr4:102487239-102487406:-	675
BANK1	chr4:102954039-102970007:+	5HSAR030176	chr4:102954029-102954094:+	133
AGXT2L1	chr4:109903504-109903592:-	5HSAR027705	chr4:109903607-109903675:-	181

HS3ST1	chr4:11010728-11010818:-	5HSAR019557	chr4:11010821-11039480:-	323
AP1AR	chr4:113372600-113372696:+	5HSAR013894	chr4:113372428-113372508:+	353
ALPK1	chr4:113456413-113518382:+	5HSAR016007	chr4:113437978-113438049:+<br>chr4:113456426-113518377:+	227
C4orf21	chr4:113773782-113777486:-	5HSAR054991	chr4:113773831-113777513:-	211
ARSJ	chr4:115119440-115119538:-	5HSAR010655	chr4:115119586-115119660:-<br>chr4:115119440-115119574:-	888
SPON2	chr4:1155860-1156425:-	5HSAR023405	chr4:1156878-1156955:-	252
UGT8	chr4:115763377-115763485:+	5HSAR015480	chr4:115763028-115763138:+<br>chr4:115763223-115763333:+	514
TRAM1L1	chr4:118225998-118226098:-	5HSAR001919	chr4:118226055-118226180:-	187
NDST3	chr4:119194425-119194513:+	5HSAR015934	chr4:119174973-119175092:+<br>chr4:119175112-119175189:+	403
USP53	chr4:120380273-120380362:+	5HSAR010321	chr4:120353255-120354714:+<br>chr4:120354778-120358179:+	1066
C4orf3	chr4:120441139-120441232:-	5HSAR016700	chr4:120441182-120441343:-	278
PRDM5	chr4:122063214-122063305:-	5HSAR020786	chr4:122063243-122063437:-	250
CTBP1	chr4:1232744-1232838:-	5HSAR026443	chr4:1225276-1232743:-<br>chr4:1225123-1225221:-	165
NUDT6	chr4:124038275-124058125:-	5HSAR019164	chr4:124053216-124058296:-	437
SPRY1	chr4:124537499-124542196:+	5HSAR011004	chr4:124537502-124542171:+	187
SCLT1	chr4:130233709-130233802:-	5HSAR009547	chr4:130233744-130233857:-	506
C4orf33	chr4:130234554-130243215:+	5HSAR020954	chr4:130234291-130234380:+<br>chr4:130234430-130234501:+	364
LOC100132117	chr4:132894933-132895028:+	5HSAR044634	chr4:132893632-132893847:+<br>chr4:132893942-132894208:+	1476
LOC100128747	chr4:132904539-132904629:+	5HSAR044638	chr4:132903550-132903765:+<br>chr4:132903860-132904249:+	1157
PCDH10	chr4:134290648-134290745:+	5HSAR023379	chr4:134290056-134290217:+<br>chr4:134290261-134290455:+	826
PCDH18	chr4:138672693-138672789:-	5HSAR012391	chr4:138672849-138672983:-<br>chr4:138672701-138672778:-	387
NDUFC1	chr4:140436403-140436498:-	5HSAR052486	chr4:140436539-140443099:-	294

TBC1D9	chr4:141896650-141896738:-	5HSAR021432	chr4:141896791-141896880:-	272
IL15	chr4:142859977-142860067:+	5HSAR029209	chr4:142777346-142777546:+<br>chr4:142796804-142796884:+	845
ABCE1	chr4:146238962-146245014:+	5HSAR010976	chr4:146238665-146238796:+<br>chr4:146238814-146238957:+	440
LRBA	chr4:152155245-152155336:-	5HSAR017360	chr4:152156098-152156313:-	474
SH3D19	chr4:152315966-152316054:-	5HSAR011658	chr4:152316075-152316140:-	189
FAM160A1	chr4:152706761-152706861:+	5HSAR011397	chr4:152595340-152623201:+<br>chr4:152706756-152706821:+	575
FBXW7	chr4:153522938-153523039:-	5HSAR031256	chr4:153493459-153493524:-	177
PROM1	chr4:15686628-15686717:-	5HSAR029578	chr4:15686757-15686837:-	212
ETFDH	chr4:159812986-159813058:+	5HSAR025769	chr4:159812824-159812901:+	332
NAF1	chr4:164307330-164307424:-	5HSAR011660	chr4:164307357-164307503:-	194
SC4MOL	chr4:166478442-166478528:+	5HSAR014802	chr4:166478405-166478518:+	130
TMEM129	chr4:1692363-1692457:-	5HSAR010827	chr4:1692745-1692813:-<br>chr4:1692609-1692725:-	520
PALLD	chr4:169826061-169839116:+	5HSAR043013	chr4:169825916-169826014:+	522
HMGB2	chr4:174491376-174492079:-	5HSAR021109	chr4:174492101-174492166:-	193
HAND2	chr4:174687016-174687113:-	5HSAR029331	chr4:174687747-174687947:-<br>chr4:174687215-174687385:-	938
ADAM29	chr4:176133172-176133251:+	5HSAR012291	chr4:176076107-176076175:+<br>chr4:176133085-176133210:+	483
GPM6A	chr4:176970373-177160509:-	5HSAR017753	chr4:176970953-176971051:-<br>chr4:176970667-176970888:-	211
CASP3	chr4:185796602-185806696:-	5HSAR031347	chr4:185806708-185807572:-	263
SORBS2	chr4:186842963-186933431:-	5HSAR026937	chr4:186969096-186969191:-<br>chr4:186842991-186933439:-	306
WHSC1	chr4:1870855-1872179:+	5HSAR023696	chr4:1864372-1865299:+<br>chr4:1865378-1870904:+	494
FAT1	chr4:187867976-187881889:-	5HSAR028717	chr4:187881853-187881927:-	188
WHSC2	chr4:1980518-1980600:-	5HSAR018131	chr4:1980545-1980757:-	240

LOC100131836	chr4:21467072-21467163:+	5HSAR005053	chr4:21466966-21467049:+<br>chr4:21467062-21467157:+	250
GPR125	chr4:22126506-22126597:-	5HSAR013525	chr4:22126613-22126711:-	265
ZFYVE28	chr4:2313111-2325530:-	5HSAR041702	chr4:2336211-2336279:-<br>chr4:2336085-2336159:-	563
CCDC149	chr4:24523600-24523698:-	5HSAR054503	chr4:24505808-24590865:-	99
RBPJ	chr4:25973247-25973331:+	5HSAR028413	chr4:25931749-25973277:+	389
HTT	chr4:3046255-3046350:+	5HSAR029737	chr4:3046226-3046291:+	145
C4orf44	chr4:3220659-3220747:+	5HSAR012012	chr4:3220601-3220666:+	183
RGS12	chr4:3341713-3341792:+	5HSAR001841	chr4:3341556-3341759:+	271
TBC1D1	chr4:37569358-37580111:+	5HSAR020746	chr4:37569271-37569348:+	343
TLR10	chr4:38453607-38453800:-	5HSAR025065	chr4:38454218-38454289:-<br>chr4:38453827-38453943:-	638
KLHL5	chr4:38723108-38740667:+	5HSAR032912	chr4:38722966-38723083:+	359
UGDH	chr4:39199528-39205382:-	5HSAR054232	chr4:39205330-39205515:-	323
N4BP2	chr4:39775247-39775355:+	5HSAR017258	chr4:39751479-39751544:+<br>chr4:39751553-39775302:+	338
NSUN7	chr4:40447381-40447467:+	5HSAR012936	chr4:40446708-40446773:+	495
SLC30A9	chr4:41687321-41687425:+	5HSAR009898	chr4:41687341-41687406:+	146
SHISA3	chr4:42094723-42094830:+	5HSAR011637	chr4:42094621-42094764:+	218
TMEM128	chr4:4300254-4300352:-	5HSAR000994	chr4:4300639-4300830:-<br>chr4:4300510-4300611:-	582
GABRA2	chr4:46085481-46085578:-	5HSAR026879	chr4:46085929-46086003:-<br>chr4:46085580-46085846:-	673
USP46	chr4:53217235-53217325:-	5HSAR029524	chr4:53217418-53217510:-	282
EVC2	chr4:5750264-5761542:-	5HSAR051238	chr4:5761655-5762149:-<br>chr4:5761472-5761564:-	732
JAKMIP1	chr4:6165479-6165562:-	5HSAR030025	chr4:6165525-6253040:-	486
PPP2R2C	chr4:6524858-6524954:-	5HSAR011199	chr4:6525041-6525106:-	370

C4orf40	chr4:71054594-71054681:+	5HSAR000848	chr4:71054573-71054647:+	189
ENAM	chr4:71713528-71714081:+	5HSAR026925	chr4:71713356-71713505:+	281
UTP3	chr4:71773163-71773258:+	5HSAR015814	chr4:71773082-71773168:+	199
RUFY3	chr4:71807060-71807154:+	5HSAR026439	chr4:71806601-71806684:+<br>chr4:71806916-71806984:+	595
CDKL2	chr4:76770197-76774305:-	5HSAR015058	chr4:76770198-76774527:-	525
SHROOM3	chr4:77576142-77576229:+	5HSAR013447	chr4:77575334-77575468:+<br>chr4:77575591-77575671:+	953
FRAS1	chr4:79198081-79198187:+	5HSAR013559	chr4:79197833-79197976:+<br>chr4:79198023-79198151:+	440
SLC10A6	chr4:87989293-87989393:-	5HSAR024010	chr4:87989352-87989435:-	148
DSPP	chr4:88748732-88751084:+	5HSAR027578	chr4:88748705-88748785:+	120
DRD5	chr4:9392668-9392751:+	5HSAR030735	chr4:9392552-9392623:+	396
SMARCAD1	chr4:95348207-95348568:+	5HSAR030056	chr4:95348057-95348523:+	255
ST8SIA4	chr5:100266559-100266653:-	5HSAR024195	chr5:100266698-100266844:-	311
PAM	chr5:102229707-102229798:+	5HSAR015549	chr5:102229433-102229510:+	373
FER	chr5:108131821-108161782:+	5HSAR022795	chr5:108111438-108131718:+<br>chr5:108131772-108131837:+	384
C5orf13	chr5:111119369-111119456:-	5HSAR016520	chr5:111119606-111119770:-<br>chr5:111119473-111119544:-	479
EPB41L4A	chr5:111782636-111782731:-	5HSAR011737	chr5:111782835-111782906:-	274
MCC	chr5:112852011-112852107:-	5HSAR017348	chr5:112852259-112852426:-	416
CSNK1G3	chr5:122909165-122909256:+	5HSAR015413	chr5:122875710-122875928:+<br>chr5:122876070-122876162:+	719
3-Mar	chr5:126281763-126393974:-	5HSAR012667	chr5:126394141-126394242:-<br>chr5:126281786-126394045:-	455
CDC42SE2	chr5:130722991-130723085:+	5HSAR024753	chr5:130679618-130679695:+<br>chr5:130679711-130722933:+	612
P4HA2	chr5:131582219-131590356:-	5HSAR021181	chr5:131590434-131590655:-	564
CDKL3	chr5:133730114-133730620:-	5HSAR019832	chr5:133730132-133730629:-	119

SAR1B	chr5:133987609-133996381:-	5HSAR049169	chr5:133996342-133996413:-	134
SMAD5	chr5:135517254-135517348:+	5HSAR024621	chr5:135511475-135517186:+<br>chr5:135517191-135517310:+	444
FAM13B	chr5:137382700-137384685:-	5HSAR018442	chr5:137384629-137384745:-	534
SRA1	chr5:139917258-139917353:-	5HSAR025191	chr5:139917770-139917838:-<br>chr5:139917693-139917761:-	605
NDUFA2	chr5:140007353-140007451:-	5HSAR053390	chr5:140007358-140007447:-	202
PCDHA9	chr5:140208185-140208264:+	5HSAR017367	chr5:140207642-140207725:+<br>chr5:140207744-140207818:+	724
PCDHAC1	chr5:140286560-140286661:+	5HSAR003341	chr5:140286488-140286628:+	176
PCDHAC2	chr5:140326439-140326535:+	5HSAR057385	chr5:140326034-140326411:+	605
PCDHGA12	chr5:140790424-140790510:	5HSAR019993	chr5:140790374-140790508:+	169
RNF14	chr5:141333482-141338123:+	5HSAR013575	chr5:141330453-141330545:+<br>chr5:141330546-141330623:+	517
FGF1	chr5:141973877-142045757:-	5HSAR019425	chr5:142045996-142046136:-<br>chr5:141973880-142045902:-	565
YIPF5	chr5:143529713-143530107:-	5HSAR025603	chr5:143530055-143530237:-	441
LARS	chr5:145542250-145542341:-	5HSAR015057	chr5:145542358-145542423:-	238
STK32A	chr5:146594952-146599390:+	5HSAR015509	chr5:146594902-146599306:+	280
SPINK6	chr5:147562806-147562852:+	5HSAR001933	chr5:147562629-147562727:+<br>chr5:147562733-147562852:+	303
PDE6A	chr5:149304430-149304524:-	5HSAR016570	chr5:149304448-149304546:-	120
ARSI	chr5:149662130-149662234:-	5HSAR030277	chr5:149662558-149662677:-<br>chr5:149662175-149662462:-	589
NDST1	chr5:149880911-149881009:+	5HSAR030406	chr5:149867874-149880628:+<br>chr5:149880656-149880721:+	502
MYOZ3	chr5:150021100-150022696:+	5HSAR025069	chr5:150020854-150020946:+<br>chr5:150020993-150021082:+	587
DCTN4	chr5:150116207-150117911:-	5HSAR014341	chr5:150118096-150118248:-<br>chr5:150117874-150118092:-	441
ZNF300	chr5:150263105-150263661:-	5HSAR058390	chr5:150264551-150264712:-<br>chr5:150264462-150264548:-	428
SLC36A1	chr5:150807489-150818546:+	5HSAR013822	chr5:150807392-150807481:+	217

FBXL7	chr5:15553693-15553785:+	5HSAR014215	chr5:15553455-15553553:+	481
SGCD	chr5:155686728-155686820:+	5HSAR023914	chr5:155686398-155686505:+<br>chr5:155686509-155686685:+	476
HAVCR1	chr5:156417533-156417628:-	5HSAR029610	chr5:156417651-156417956:-	533
MED7	chr5:156499021-156502059:-	5HSAR010478	chr5:156502048-156502311:-	392
SLU7	chr5:159774880-159778679:-	5HSAR002453	chr5:159774882-159778731:-	155
GABRA1	chr5:161210200-161210394:+	5HSAR026927	chr5:161209985-161210134:+<br>chr5:161210191-161210268:+	315
HMMR	chr5:162820220-162820310:+	5HSAR028370	chr5:162820177-162820290:+	216
MYO10	chr5:16988918-16989015:-	5HSAR025207	chr5:16989106-16989204:-	468
NKX2-5	chr5:172594693-172594773:-	5HSAR049309	chr5:172594709-172594894:-	229
HRH2	chr5:175042750-175042842:+	5HSAR029728	chr5:175041246-175041326:+<br>chr5:175041343-175041408:+	1773
TSPAN17	chr5:176007115-176007222:+	5HSAR002177	chr5:176007048-176007113:+	229
RAB24	chr5:176662896-176662993:-	5HSAR016235	chr5:176662986-176663210:-	455
MXD3	chr5:176671438-176671534:-	5HSAR018106	chr5:176671753-176671827:-	461
LOC729595	chr5:179038740-179038836:+	5HSAR044723	chr5:179038668-179038763:+	198
MAML1	chr5:179092624-179092719:+	5HSAR014319	chr5:179092483-179092626:+	263
BTNL8	chr5:180258910-180261980:+	5HSAR035604	chr5:180258692-180258823:+	312
TRIM7	chr5:180559682-180563242:-	5HSAR001975	chr5:180563716-180563919:-	421
CDH12	chr5:22114543-22114625:-	5HSAR012595	chr5:22889327-22889455:-<br>chr5:22889151-22889285:-	1087
CDH10	chr5:24535307-24535385:-	5HSAR057360	chr5:24680688-24680786:-<br>chr5:24680579-24680674:-]	81
UGT3A1	chr5:36024343-36033114:-	5HSAR055350	chr5:36033112-36036872:-	220
NUP155	chr5:37400224-37406595:-	5HSAR018214	chr5:37400240-37406586:-	140
GDNF	chr5:37870656-37875429:-	5HSAR030141	chr5:37870657-37875439:-	200

C6	chr5:41239090-41297122:-	5HSAR030551	chr5:41297184-41297267:- chr5:41239109-41297129:-	265
PELO	chr5:52131897-52131985:+	5HSAR002296	chr5:52119563-52119685:+ chr5:52119720-52131507:+	985
HSPB3	chr5:53787288-53787376:+	5HSAR003220	chr5:53787214-53787282:+	175
GPBP1	chr5:56507940-56545714:+	5HSAR059496	chr5:56507051-56507176:+ chr5:56507392-56507592:+	1010
PDE4D	chr5:59517183-59517267:-	5HSAR048783	chr5:59517253-59819608:-	246
FAM159B	chr5:64022112-64022206:+	5HSAR034650	chr5:64021919-64022071:+	316
C5orf44	chr5:64956555-64956643:+	5HSAR013700	chr5:64956398-64956469:+	330
MAST4	chr5:65928148-65928239:+	5HSAR014936	chr5:65928010-65928102:+	308
PIK3R1	chr5:67620231-67620319:+	5HSAR025953	chr5:67620031-67620318:+	312
SERF1B	chr5:69356946-69357034:+	5HSAR041491	chr5:69356849-69356962:+	201
SERF1A	chr5:70232364-70232452:+	5HSAR021330	chr5:70232267-70232380:+	207
UTP15	chr5:72897480-72898925:+	5HSAR014219	chr5:72897382-72898874:+	223
ENC1	chr5:73968067-73971956:-	5HSAR020998	chr5:73972867-73972947:- chr5:73972405-73972794:-	1131
COL4A3BP	chr5:74842789-74842878:-	5HSAR025760	chr5:74843021-74843188:-	422
AGGF1	chr5:76362250-76362347:+	5HSAR017810	chr5:76362206-76362304:+	360
ARSB	chr5:78316828-78316922:-	5HSAR021319	chr5:78317937-78318101:- chr5:78317699-78317830:-	1286
HOMER1	chr5:78844354-78844440:-	5HSAR020092	chr5:78844771-78845214:- chr5:78844583-78844708:-	1103
FASTKD3	chr5:7921197-7921263:-	5HSAR002473	chr5:7921197-7921246:-	172
MTX3	chr5:79321784-79322795:-	5HSAR033245	chr5:79321798-79322817:-	140
CKMT2	chr5:80564907-80582707:+	5HSAR015133	chr5:80575627-80575701:+	82
ZCCHC9	chr5:80633207-80636332:+	5HSAR012654	chr5:80633165-80633272:+	133
CCNH	chr5:86744368-86744468:-	5HSAR026323	chr5:86744471-86744542:-	225

CETN3	chr5:89741235-89741334:-	5HSAR001115	chr5:89741273-89741359:-	125
LYSMD3	chr5:89856863-89861084:-	5HSAR010191	chr5:89861030-89861098:-	148
ZDHHC11	chr5:903718-903802:-	5HSAR012606	chr5:903750-904022:-	384
ARRDC3	chr5:90714666-90714764:-	5HSAR016662	chr5:90714680-90714811:-	240
LNPEP	chr5:96297529-96297615:+	5HSAR021458	chr5:96297153-96297275:+	514
CCNC	chr6:100123125-100123230:-	5HSAR001437	chr6:100116260-100117539:-	287
LIN28B	chr6:105511723-105511818:+	5HSAR026959	chr6:105511734-105511802:+	203
RTN4IP1	chr6:107183590-107183680:-	5HSAR001829	chr6:107183816-107183887:-<br>chr6:107183661-107183789:-	477
C6orf203	chr6:107456612-107467657:+	5HSAR016782	chr6:107456390-107456548:+<br>chr6:107456615-107467652:+	607
SCML4	chr6:108200225-108252064:-	5HSAR018826	chr6:108252144-108252212:-<br>chr6:108252023-108252106:-	251
TMEM14C	chr6:10831407-10832832:+	5HSAR031861	chr6:10831208-10831297:+	371
NR2E1	chr6:108594573-108594662:+	5HSAR019252	chr6:108594008-108594103:+<br>chr6:108594223-108594462:+	755
WASF1	chr6:110555498-110588599:-	5HSAR020927	chr6:110607725-110607805:-<br>chr6:110607460-110607672:-	692
KIAA1919	chr6:111687489-111687596:+	5HSAR001013	chr6:111687220-111687444:+	422
FYN	chr6:112147948-112274496:-	5HSAR028680	chr6:112208509-112300898:-	579
TSPYL1	chr6:116707687-116707773:-	5HSAR009383	chr6:116707774-116707920:-	287
DSE	chr6:116708077-116827106:+	5HSAR014704	chr6:116707995-116827099:+	194
RWDD1	chr6:117012647-117016715:+	5HSAR016298	chr6:116999417-116999500:+<br>chr6:117000718-117000792:+	693
MAN1A1	chr6:119711930-119712010:-	5HSAR014877	chr6:119711965-119712464:-	442
NKAIN2	chr6:124166951-124167044:+	5HSAR000864	chr6:124166886-124166975:+	277
RNF146	chr6:127648938-127649454:+	5HSAR014029	chr6:127643077-127645136:+<br>chr6:127645207-127648932:+	399
PHACTR1	chr6:12825916-12826962:+	5HSAR024935	chr6:12825821-12825946:+	189

C6orf192	chr6:133161289-133161385:-	5HSAR00 1972	chr6:133161326-133161406:-	152
TBC1D7	chr6:13435110-13436732:-	5HSAR02 4101	chr6:13435110-13436737:-	108
SLC2A12	chr6:134415312-134415415:-	5HSAR01 6312	chr6:134415325-134415393:-	171
HBS1L	chr6:135417523-135417616:-	5HSAR02 9298	chr6:135417553-135417627:-	207
AHI1	chr6:135855069-135860454:-	5HSAR01 8908	chr6:135858666-135860032:-	237
GFOD1	chr6:13595102-13595190:-	5HSAR01 4509	chr6:13595612-13595758:- chr6:13595408-13595479:-	665
BCLAF1	chr6:136642698-136645491:-	5HSAR02 0557	chr6:136645440-136652632:-	253
NHSL1	chr6:138862121-138862217:-	5HSAR01 8613	chr6:138862169-138862252:-	152
REPS1	chr6:139350513-139350601:-	5HSAR01 1568	chr6:139350642-139351037:-	579
C6orf94	chr6:144227270-144227369:+	5HSAR01 1676	chr6:144227292-144227357:+	104
LATS1	chr6:150064956-150065050:-	5HSAR01 7371	chr6:150065090-150080953:-	548
PPP1R14C	chr6:150505925-150506021:+	5HSAR02 1301	chr6:150505931-150506002:+	141
AKAP12	chr6:151688610-151688688:+	5HSAR02 1656	chr6:151688489-151688584:+	330
SYNE1	chr6:152991160-152991250:-	5HSAR02 1249	chr6:152991296-152991370:-	437
FBXO5	chr6:153338415-153346128:-	5HSAR01 6544	chr6:153338443-153346297:-	407
TIAM2	chr6:155579894-155603412:+	5HSAR02 4712	chr6:155579803-155579892:+	185
ZDHHC14	chr6:157722939-157723041:+	5HSAR00 1505	chr6:157722848-157722922:+	497
SYNJ2	chr6:158374601-158374700:+	5HSAR04 9699	chr6:158358164-158358298:+ chr6:158369839-158369916:+	740
TAGAP	chr6:159381796-159382417:-	5HSAR02 7481	chr6:159386065-159386151:- chr6:159382510-159386035:-	699
TCP1	chr6:160126431-160126516:-	5HSAR02 1544	chr6:160128877-160130490:- chr6:160126995-160128874:-	614
PNLDC1	chr6:160141377-160141750:+	5HSAR00 3786	chr6:160141330-160141738:	171
AGPAT4	chr6:161573236-161573316:-	5HSAR01 7898	chr6:161614990-161615097:- chr6:161573279-161614985:-	227

UNC93A	chr6:167624873-167624967:+	5HSAR018530	chr6:167624813-167624950:+	175
TCP10	chr6:167716352-167716431:-	5HSAR010331	chr6:167716403-167717896:-	212
KIF25	chr6:168173027-168173114:+	5HSAR015911	chr6:168161474-168161548:+	262
SOX4	chr6:21702651-21702744:+	5HSAR024456	chr6:21702121-21702267:+<br>chr6:21702273-21702476:+	794
KAAG1	chr6:24465764-24465846:+	5HSAR013215	chr6:24465114-24465188:+<br>chr6:24465219-24465299:+	737
KIAA0319	chr6:24708854-24709283:-	5HSAR053643	chr6:24709409-24754037:-<br>chr6:24708873-24709310:-	643
C6orf62	chr6:24826876-24826965:-	5HSAR016479	chr6:24827051-24827293:-	507
TRIM38	chr6:26074632-26074729:+	5HSAR020762	chr6:26071291-26071404:+<br>chr6:26074564-26074635:+	435
BTN3A2	chr6:26473534-26476389:+	5HSAR027086	chr6:26473422-26473508:+<br>chr6:26476197-26476389:+	249
BTN3A1	chr6:26513666-26513770:+	5HSAR020463	chr6:26510492-26510572:+<br>chr6:26510610-26513667:+	368
WRNIP1	chr6:2710763-2710855:+	5HSAR022083	chr6:2710705-2710779:+	191
ZNF184	chr6:27548053-27548134:-	5HSAR020531	chr6:27548146-27548789:-	285
ZKSCAN3	chr6:28425788-28435342:+	5HSAR021910	chr6:28425727-28435312:+	216
TRIM15	chr6:30239356-30239440:+	5HSAR026040	chr6:30238968-30239096:+<br>chr6:30239101-30239190:+	479
GNL1	chr6:30631960-30632047:-	5HSAR042145	chr6:30633127-30633234:-<br>chr6:30632998-30633087:-	1391
VARS2	chr6:30990091-30990167:+	5HSAR056082	chr6:30989975-30990085:+<br>chr6:30989975-30990085:+	81
PSORS1C1	chr6:31201565-31205388:+	5HSAR038884	chr6:31190618-31201494:+	289
CCHCR1	chr6:31233357-31233447:-	5HSAR024335	chr6:31233465-31233536:-<br>chr6:31233363-31233434:-	189
SKIV2L	chr6:32034867-32034948:+	5HSAR016292	chr6:32034607-32034726:+	389
DOM3Z	chr6:32047432-32047765:-	5HSAR025216	chr6:32047725-32047790:-	333
BRD2	chr6:33048570-33048653:+	5HSAR026267	chr6:33044441-33044626:+<br>chr6:33044645-33047731:+	1701
SLC39A7	chr6:33276912-33277000:+	5HSAR025969	chr6:33276748-33276852:+<br>chr6:33276864-33276932:+	420

B3GALT4	chr6:33353085-33353174:+	5HSAR030033	chr6:33352935-33353096:+	280
WDR46	chr6:33364926-33365009:-	5HSAR033215	chr6:33365203-33365271:-	357
DAXX	chr6:33397681-33398665:-	5HSAR022527	chr6:33398656-33398763:-	204
CUTA	chr6:33493756-33493846:-	5HSAR002287	chr6:33493800-33494006:-	288
LEMD2	chr6:33856856-33860135:-	5HSAR018522	chr6:33862595-33862660:- chr6:33856876-33862493:-	338
GRM4	chr6:34209252-34209351:-	5HSAR028529	chr6:34209263-34209388:-	170
TCP11	chr6:35215825-35215917:-	5HSAR021394	chr6:35215979-35216984:-	500
PPARD	chr6:35418430-35422892:	5HSAR050245	chr6:35418355-35418423:+	195
FKBP5	chr6:35718580-35764568:-	5HSAR027100	chr6:35796143-35804293:-	203
FGD2	chr6:37081471-37081571:+	5HSAR021722	chr6:37081464-37081544:+	171
BTBD9	chr6:38670063-38671609:-	5HSAR024460	chr6:38671643-38671741:-	317
LRFN2	chr6:40508831-40662730:-	5HSAR001278	chr6:40662949-40663014:- chr6:40508842-40662863:-	465
PECI	chr6:4078905-4080796:-	5HSAR015709	chr6:4078916-4080793:-	117
TREML2	chr6:41276725-41276827:-	5HSAR013302	chr6:41276747-41276845:-	179
FOXP4	chr6:41622512-41641476:+	5HSAR011494	chr6:41622390-41641470:+	458
CCND3	chr6:42016257-42124253:-	5HSAR027990	chr6:42124403-42124561:-	417
CUL7	chr6:43128505-43129415:-	5HSAR053223	chr6:43129377-43129574:-	332
SRF	chr6:43247277-43247372:+	5HSAR026721	chr6:43246899-43247111:+	475
CLIC5	chr6:46091239-46091341:-	5HSAR012703	chr6:46091256-46091429:-	318
TFAP2D	chr6:50789647-50789727:+	5HSAR003677	chr6:50789224-50789307:+ chr6:50789310-50789423:+	512
FARS2	chr6:5206836-5313802:+	5HSAR015080	chr6:5206656-5206796:+	331
BEND6	chr6:56954480-56954567:+	5HSAR014507	chr6:56927834-56927950:+ chr6:56928208-56954559:+	586

LOC100132588	chr6:58378559-58378652:-	5HSAR005539	chr6:58379297-58379503:- chr6:58379004-58379270:-	952
FAM135A	chr6:71192910-71194766:+	5HSAR039157	chr6:71179926-71180018:+ chr6:71180035-71192947:+	133
RIMS1	chr6:72979461-72979546:+	5HSAR043048	chr6:72979240-72979314:+ chr6:72979347-72979469:+	312
CAGE1	chr6:7324128-7324225:-	5HSAR015859	chr6:7334735-7334938:- chr6:7334438-7334527:-	721
KHDC1	chr6:74008962-74029298:-	5HSAR010432	chr6:74029257-74029325:-	424
DDX43	chr6:74161257-74161349:+	5HSAR024206	chr6:74161169-74161246:+	344
LOC644058	chr6:7483995-7484089:+	5HSAR044741	chr6:7483996-7484088:+	104
FILIP1	chr6:76181409-76259942:-	5HSAR013926	chr6:76259952-76260035:-	373
LCA5	chr6:80285331-80285431:-	5HSAR043262	chr6:80285422-80285499:-	457
SLC35B3	chr6:8379620-8380507:-	5HSAR016567	chr6:8380590-8380667:-	377
DOPEY1	chr6:83863232-83863415:+	5HSAR002437	chr6:83863217-83863398:+	260
CYB5R4	chr6:84626118-84626220:+	5HSAR024086	chr6:84626090-84626218:+	132
C6orf163	chr6:88111442-88111536:+	5HSAR031712	chr6:88111326-88111397:+	247
CNR1	chr6:88911713-88914138:-	5HSAR048402	chr6:88914107-88932249:-	452
RNGTT	chr6:89729848-89729943:-	5HSAR025693	chr6:89729877-89730017:-	220
GABRR1	chr6:89983761-89983850:-	5HSAR019080	chr6:89984070-89984210:- chr6:89983954-89984037:-	455
ANKRD6	chr6:90333331-90333414:+	5HSAR009634	chr6:90333281-90333364:+	341
FUT9	chr6:96668644-96757752:+	5HSAR014580	chr6:96570602-96570718:+ chr6:96570787-96757746:+	341
ZNHIT1	chr7:100648110-100648196:+	5HSAR011342	chr7:100648036-100648125:+	492
RABL5	chr7:100746519-100748183:-	5HSAR012568	chr7:100751727-100751795:- chr7:100748158-100751710:-	257
LOC100132214	chr7:101937651-101937732:-	5HSAR044172	chr7:101945094-101945162:-	387
FAM185A	chr7:102176792-102176890:+	5HSAR021807	chr7:102176725-102176868:+	256

LRRC17	chr7:102361500-102361596:+	5HSAR033678	chr7:102340637-102340759:+ chr7:102340797-102361574:+	389
FBXL13	chr7:102483327-102483425:-	5HSAR026018	chr7:102502084-102502248:- chr7:102483396-102501998:-	250
ARMC10	chr7:102502865-102502955:+	5HSAR011269	chr7:102502602-102502787:+	392
MLL5	chr7:104468545-104468635:+	5HSAR014598	chr7:104465880-104468608:+	534
COG5	chr7:106991671-106991763:-	5HSAR052451	chr7:106992034-106992183:-	525
CBLL1	chr7:107171734-107171844:+	5HSAR026245	chr7:107171593-107171694:+	330
PNPLA8	chr7:107943172-107943253:-	5HSAR019123	chr7:107945894-107953790:- chr7:107943193-107945882:-	407
MDFIC	chr7:114350229-114350324:+	5HSAR056989	chr7:114349571-114349684:+ chr7:114349833-114350287:+	590
THSD7A	chr7:11838098-11838200:-	5HSAR017855	chr7:11838245-11838337:-	252
KCND2	chr7:119701841-119701922:+	5HSAR029615	chr7:119701023-119701166:+	965
TSPAN12	chr7:120284054-120285130:-	5HSAR011369	chr7:120284086-120285176:-	374
RNF148	chr7:122130041-122130137:-	5HSAR003079	chr7:122130056-122130223:-	217
SPAM1	chr7:123380764-123380860:+	5HSAR043969	chr7:123352547-123352711:+ chr7:123352738-123352809:+	618
POT1	chr7:124324464-124325598:-	5HSAR026015	chr7:124357103-124357189:- chr7:124325645-124356227:-	601
LRRC4	chr7:127457930-127458028:-	5HSAR018955	chr7:127457949-127458029:-	137
AHCYL2	chr7:128802866-128802946:+	5HSAR053458	chr7:128802721-128802873:+	227
CPA5	chr7:129773475-129773562:+	5HSAR015699	chr7:129771866-129771976:+ chr7:129772001-129772075:+	567
FAM180A	chr7:135083869-135083947:-	5HSAR031740	chr7:135084004-135084075:-	266
CHRM2	chr7:136327499-136350152:+	5HSAR027865	chr7:136327486-136350112:+	297
ATP6V0A4	chr7:138106533-138133399:-	5HSAR017275	chr7:138121523-138133391:-	180
ETV1	chr7:13992327-13992424:-	5HSAR058625	chr7:13992533-13992661:-	338
EPHB6	chr7:142271026-142271107:+	5HSAR030181	chr7:142269902-142270688:+	787

FAM131B	chr7:142767309-142769256:-	5HSAR001006	chr7:142769846-142769959:-	121
CNTNAP2	chr7:145444821-145444901:+	5HSAR029598	chr7:145444397-145444810:+	516
C7orf33	chr7:147918865-147918950:+	5HSAR000030	chr7:147918618-147918845:+	361
ZNF398	chr7:148494183-148494275:+	5HSAR012006	chr7:148454527-148454592:+<br>chr7:148454595-148454666:+	851
KCNH2	chr7:150283525-150283604:-	5HSAR029834	chr7:150283583-150283663:-	324
SNX13	chr7:17946444-17946544:-	5HSAR012875	chr7:17946473-17946631:-	213
ABCB5	chr7:20621908-20629433:+	5HSAR034727	chr7:20621798-20621872:+	237
RAPGEF5	chr7:22316165-22321534:-	5HSAR026428	chr7:22321548-22321616:-	313
KLHL7	chr7:23112878-23129944:+	5HSAR054374	chr7:23112501-23112632:+<br>chr7:23112833-23129934:+	521
STK31	chr7:23716819-23718261:+	5HSAR015271	chr7:23716632-23716697:+	380
DFNA5	chr7:24755919-24763606:-	5HSAR043525	chr7:24750875-24764138:-	88
C7orf31	chr7:25185453-25185865:-	5HSAR000301	chr7:25186214-25186324:-<br>chr7:25185920-25186123:-	561
HOXA3	chr7:27116785-27116868:-	5HSAR011327	chr7:27133075-27133152:-<br>chr7:27116865-27133052:-	339
EVX1	chr7:27249078-27249174:+	5HSAR011341	chr7:27248886-27248987:+	486
FKBP14	chr7:30032650-30032748:-	5HSAR015100	chr7:30032725-30032790:-	144
GARS	chr7:30600965-30601062:+	5HSAR028861	chr7:30600725-30600946:+	357
CCDC129	chr7:31524408-31559163:+	5HSAR000701	chr7:31523643-31523957:+<br>chr7:31524047-31524313:+	993
KBTBD2	chr7:32885742-32885826:-	5HSAR020645	chr7:32885959-32886051:-<br>chr7:32885834-32885929:-	659
BBS9	chr7:33136094-33152389:+	5HSAR018319	chr7:33135699-33135905:+	513
TBX20	chr7:35259757-35259845:-	5HSAR048394	chr7:35259864-35260106:-	480
HERPUD2	chr7:35700466-35700556:-	5HSAR026790	chr7:35700479-35700622:-	659
EEPD1	chr7:36160367-36160458:+	5HSAR009277	chr7:36159613-36159684:+<br>chr7:36160303-36160446:+	640

ELMO1	chr7:36901145-36992127:-	5HSAR023955	chr7:36992122-36992214:-	197
C7orf25	chr7:42917025-42918116:-	5HSAR000657	chr7:42917035-42918191:-	192
HECW1	chr7:43120461-43250029:+	5HSAR019598	chr7:43118727-43119035:+ chr7:43120287-43120373:+	605
GCK	chr7:44195078-44195167:-	5HSAR052976	chr7:44195470-44195538:- chr7:44195256-44195336:-	470
DDX56	chr7:44580020-44580114:-	5HSAR017738	chr7:44580407-44580472:-	643
TBRG4	chr7:45115362-45117812:-	5HSAR011259	chr7:45115392-45117821:-	126
PAPOLB	chr7:4867968-4868061:-	5HSAR014916	chr7:4868065-4868151:- chr7:4	184
VWC2	chr7:49785478-49785577:+	5HSAR000597	chr7:49783989-49784084:+ chr7:49784168-49785518:+	556
WIPI2	chr7:5220560-5220657:+	5HSAR015848	chr7:5220464-5220622:+	232
TNRC18	chr7:5427404-5427496:-	5HSAR022539	chr7:5427449-5427526:-	349
LANCL2	chr7:55401140-55401212:+	5HSAR020227	chr7:55400661-55400726:+ chr7:55401030-55401110:+	578
PSPH	chr7:56056400-56067176:-	5HSAR022792	chr7:56086446-56086544:- chr7:56086326-56086412:-	729
LOC730275	chr7:56845460-56845560:+	5HSAR044139	chr7:56844332-56844877:+ chr7:56844988-56845512:+	1250
OCM	chr7:5886955-5887046:+	5HSAR014473	chr7:5886971-5887039:+	92
ZNF727	chr7:63143340-63143434:+	5HSAR029395	chr7:63143259-63143339:+	179
ZNF736	chr7:63411868-63411963:+	5HSAR056499	chr7:63411788-63411907:+	322
ZNF138	chr7:63892289-63892381:+	5HSAR039283	chr7:63892210-63892326:+	176
LOC441239	chr7:63987553-63987638:-	5HSAR044143	chr7:63987737-63987868:-	361
ZNF117	chr7:64079234-64079816:-	5HSAR012683	chr7:64088761-64088844:- chr7:64088664-64088729:-	1285
TPST1	chr7:65342755-65342847:+	5HSAR020321	chr7:65307696-65307767:+ chr7:65307905-65342826:+	352
C7orf42	chr7:66023812-66044287:+	5HSAR000354	chr7:66023762-66044279:+	264
POM121	chr7:72034799-72034886:+	5HSAR011303	chr7:71987933-71988031:+ chr7:71999121-71999195:+	977

GTF2IRD1	chr7:73506364-73560346:+	5HSAR029151	chr7:73506059-73506382:+	393
RHBDD2	chr7:75349227-75349327:+	5HSAR011374	chr7:75346337-75348680:+	629
DTX2	chr7:75947687-75947762:+	5HSAR025648	chr7:75928929-75937592:+	556
RPA3	chr7:7646575-7646673:-	5HSAR024401	chr7:7721362-7724724:-<br>chr7:7693394-7721356:-	1172
KIAA1324L	chr7:86432161-86432253:-	5HSAR031881	chr7:86433070-86433222:-<br>chr7:86432179-86432250:-	283
DMTF1	chr7:86630797-86632193:+	5HSAR016523	chr7:86619817-86619894:+<br>chr7:86621522-86621704:+	563
C7orf23	chr7:86686756-86686858:-	5HSAR011275	chr7:86686845-86686961:-	212
DBF4	chr7:87343876-87343983:+	5HSAR021562	chr7:87343551-87343694:+	504
FZD1	chr7:90732049-90732131:+	5HSAR031386	chr7:90731738-90731839:+	413
SAMD9L	chr7:92603221-92612052:-	5HSAR011282	chr7:92615475-92615576:-<br>chr7:92614293-92615465:-	1216
PPP1R9A	chr7:94377273-94377361:+	5HSAR054203	chr7:94377146-94377211:+	216
ADAP1	chr7:960640-960722:-	5HSAR013664	chr7:960688-960780:-	176
ZNF655	chr7:98994292-98996118:+	5HSAR002045	chr7:98993993-98994310:+	393
MCM7	chr7:99534636-99534726:-	5HSAR023199	chr7:99535897-99536178:-<br>chr7:99535804-99535869:-	1117
TAF6	chr7:99549769-99554456:-	5HSAR053531	chr7:99554614-99554904:-	552
MBLAC1	chr7:99562567-99562954:+	5HSAR029099	chr7:99562285-99562599:+	399
POLR2K	chr8:101232025-101232759:+	5HSAR024357	chr8:101232027-101232113:+	108
SPAG1	chr8:101239936-101243684:+	5HSAR002557	chr8:101239897-101239995:+	191
GRHL2	chr8:102574092-102574173:+	5HSAR031544	chr8:102573953-102574027:+	330
NCALD	chr8:102801034-102872341:-	5HSAR014193	chr8:102872492-102872572:-<br>chr8:102872386-102872457:-	369
KLF10	chr8:103737006-103737082:-	5HSAR027176	chr8:103737045-103737140:-	154
FZD6	chr8:104381407-104381607:+	5HSAR052268	chr8:104381397-104381471:+	298

NUDCD1	chr8:110411241-110411323:-	5HSAR017725	chr8:110415442-110415513:-	98
EBAG9	chr8:110621241-110632229:+	5HSAR018885	chr8:110621117-110621185:+	235
SYBU	chr8:110726065-110726731:-	5HSAR009622	chr8:110726717-110726788:-	362
BLK	chr8:11389429-11438142:+	5HSAR027444	chr8:11388937-11389014:+<br>chr8:11389022-11389111:+	581
NEIL2	chr8:11665171-11666365:+	5HSAR029797	chr8:11664610-11665135:+	293
CTSB	chr8:11748373-11759299:-	5HSAR027371	chr8:11756331-11759331:-	324
EXT1	chr8:119192467-119192562:-	5HSAR026105	chr8:119192948-119193019:-<br>chr8:119192649-119192909:-	773
ZNF705D	chr8:11999335-12003111:+	5HSAR012264	chr8:11984388-11999327:+	422
MRPL13	chr8:121526516-121526606:-	5HSAR018211	chr8:121526719-121526787:-<br>chr8:121526620-121526709:-	313
FAM84B	chr8:127638817-127638905:-	5HSAR003562	chr8:127639521-127639622:-	284
ST3GAL1	chr8:134557450-134557743:-	5HSAR019254	chr8:134653219-134653317:-<br>chr8:134627293-134653157:-	829
C8orf48	chr8:13468782-13468871:+	5HSAR023128	chr8:13468770-13468862:+	149
COL22A1	chr8:139964598-139995056:-	5HSAR009709	chr8:139995201-139995296:-	447
SHARPIN	chr8:145230645-145230741:-	5HSAR016208	chr8:145230944-145231021:-	484
DGAT1	chr8:145521108-145521200:-	5HSAR022326	chr8:145521257-145521349:-	268
SGCZ	chr8:15139504-15139601:-	5HSAR015839	chr8:15139990-15140061:-<br>chr8:15139809-15139901:-	660
ZNF596	chr8:172601-180895:+	5HSAR010527	chr8:172507-172623:+	313
MTUS1	chr8:17617575-17617643:-	5HSAR042081	chr8:17624220-17624363:-<br>chr8:17617607-17617684:-	271
FGL1	chr8:17787344-17797079:-	5HSAR009432	chr8:17797115-17797192:-	197
PCM1	chr8:17826510-17837399:+	5HSAR019138	chr8:17824723-17824791:+<br>chr8:17824793-17824975:+	422
NAT1	chr8:18123753-18123836:+	5HSAR039277	chr8:18123467-18123667:+<br>chr8:18123672-18123752:+	380
SH2D4A	chr8:19221256-19221338:+	5HSAR049257	chr8:19215420-19215581:+	458

CSGALNACT1	chr8:19407626-19407716:-	5HSAR015500	chr8:19486989-19503603:-<br>chr8:19407757-19407882:-	654
PHYHIP	chr8:22141816-22145310:-	5HSAR010678	chr8:22145614-22145739:-	572
SLC39A14	chr8:22280790-22318168:+	5HSAR024165	chr8:22280719-22280790:+	175
KIAA1967	chr8:22518822-22519231:+	5HSAR054040	chr8:22518762-22518842:+	431
ENTPD4	chr8:23363359-23363453:-	5HSAR015669	chr8:23363394-23371066:-	164
ADAMDEC1	chr8:24297999-24306749:+	5HSAR021768	chr8:24297745-24297834:+<br>chr8:24297963-24306730:+	338
GNRH1	chr8:25336764-25336870:-	5HSAR023024	chr8:25338282-25338401:-<br>chr8:25338204-25338281:-	1710
PNMA2	chr8:26422189-26422271:-	5HSAR017323	chr8:26423086-26427292:-<br>chr8:26422492-26423053:-	770
PTK2B	chr8:27239063-27311018:+	5HSAR028777	chr8:27225029-27225757:+<br>chr8:27226712-27235918:+	808
CLU	chr8:27525039-27525125:-	5HSAR043242	chr8:27525089-27525163:-	147
FZD3	chr8:28416363-28416449:+	5HSAR018847	chr8:28413394-28416303:+	478
INTS9	chr8:28803319-28803408:-	5HSAR020657	chr8:28803438-28803608:-<br>chr8:28803358-28803435:-	299
NRG1	chr8:32624681-32624778:+	5HSAR052538	chr8:32623803-32623898:+<br>chr8:32624001-32624132:+	986
FBXO25	chr8:346975-353079:+	5HSAR017788	chr8:346901-347041:+	266
TM2D2	chr8:38972180-38972274:-	5HSAR010448	chr8:38972229-38973115:-	294
GOLGA7	chr8:41467426-41467530:+	5HSAR014376	chr8:41467374-41467478:+	201
AGPAT6	chr8:41575719-41575815:+	5HSAR022241	chr8:41574978-41575100:+<br>chr8:41575202-41575279:+	939
CSMD1	chr8:4839347-4839429:-	5HSAR027037	chr8:4839423-4839530:-	390
PLAG1	chr8:57242613-57246286:-	5HSAR024952	chr8:57286243-57286374:-	283
CHCHD7	chr8:57286870-57289726:+	5HSAR025901	chr8:57287893-57287967:+	98
SDR16C5	chr8:57391461-57395244:-	5HSAR020790	chr8:57395513-57395683:-<br>chr8:57395258-57395365:-	638
IMPAD1	chr8:58068699-58068797:-	5HSAR019489	chr8:58068733-58068840:-	283

TOX	chr8:60194101-60194190:-	5HSAR031586	chr8:60194155-60194232:-	221
CLVS1	chr8:62374869-62374940:+	5HSAR014468	chr8:62374796-62374894:+	324
MTFR1	chr8:66719555-66744741:+	5HSAR023304	chr8:66744670-66744741:+	212
CRH	chr8:67252267-67253157:-	5HSAR038540	chr8:67253219-67253380:-	333
SGK3	chr8:67868448-67868525:+	5HSAR015749	chr8:67850084-67868457:+	214
C8orf34	chr8:69514198-69514293:+	5HSAR000019	chr8:69512716-69512784:+<br>chr8:69512786-69512878:+	1592
KCNB2	chr8:73642435-73642523:+	5HSAR021708	chr8:73612339-73612407:+<br>chr8:73612468-73612674:+	588
LOC729339	chr8:7414768-7414850:+	5HSAR044880	chr8:7414400-7414705:+	475
LOC100132485	chr8:7422417-7422499:	5HSAR044881	chr8:7422106-7422354:+	415
LOC729346	chr8:7430062-7430144:+	5HSAR044882	chr8:7429751-7429999:+	416
STAU2	chr8:74783935-74814546:-	5HSAR052394	chr8:74814614-74821608:-	341
LOC100132221	chr8:7661350-7661432:-	5HSAR044889	chr8:7661495-7661800:-	475
PEX2	chr8:78058970-78074840:-	5HSAR049966	chr8:78074868-78075686:-	322
LOC729459	chr8:7920402-7920489:+	5HSAR044891	chr8:7917060-7917347:+<br>chr8:7917437-7917511:+	3513
LOC729462	chr8:7928054-7928136:+	5HSAR044893	chr8:7927743-7927991:+	416
PKIA	chr8:79591189-79673174:+	5HSAR003353	chr8:79590895-79591083:+<br>chr8:79591101-79591247:+	395
FAM164A	chr8:79740847-79740938:+	5HSAR021090	chr8:79740840-79740920:+	102
ZNF704	chr8:81896385-81949433:-	5HSAR001244	chr8:81949420-81949548:-	232
C8orf59	chr8:86316981-86318817:-	5HSAR012194	chr8:86318795-86319835:-	237
MFHAS1	chr8:8787979-8788067:-	5HSAR021257	chr8:8788329-8788418:-<br>chr8:8788138-8788221:-	563
OSGIN2	chr8:90990977-90991056:+	5HSAR010013	chr8:90983282-90983362:+<br>chr8:90983452-90990977:+	346
SLC26A7	chr8:92330972-92331055:+	5HSAR003903	chr8:92330779-92331005:+	239

RUNX1T1	chr8:93098745-93098825:-	5HSAR022572	chr8:93098897-93098971:-<br>chr8:93098804-93098872:-	340
C8orf83	chr8:93998394-94002978:-	5HSAR056260	chr8:94035839-94035913:-	473
LPPR1	chr9:102987312-102987597:+	5HSAR000435	chr9:102987188-102987289:+	266
BAAT	chr9:103173508-103185596:-	5HSAR021770	chr9:103187006-103187089:-	109
C9orf125	chr9:103279196-103289074:-	5HSAR002768	chr9:103279260-103289254:-	322
SMC2	chr9:105896534-105897486:+	5HSAR016396	chr9:105896377-105896442:+<br>chr9:105896471-105896572:+	279
SLC44A1	chr9:107046884-107046970:+	5HSAR002018	chr9:107046785-107046859:+	221
KLF4	chr9:109291275-109291357:-	5HSAR023752	chr9:109291540-109291722:-	594
IKBKAP	chr9:110733248-110736011:-	5HSAR025616	chr9:110736279-110736374:-	520
MUSK	chr9:112470908-112471005:+	5HSAR058412	chr9:112470935-112471003:+	134
ZNF483	chr9:113329400-113329496:+	5HSAR011419	chr9:113327352-113329449:+	224
PTGR1	chr9:113399524-113401782:-	5HSAR026525	chr9:113401825-113401956:-	263
CDC26	chr9:115074609-115075487:-	5HSAR010868	chr9:115075533-115077685:-	359
HDHD3	chr9:115176456-115176531:-	5HSAR001734	chr9:115178011-115178100:-<br>chr9:115176576-115177744:-	784
ASTN2	chr9:118488906-118488982:-	5HSAR040298	chr9:118489150-118489215:-<br>chr9:118489054-118489143:-	410
CDK5RAP2	chr9:122382078-122382161:-	5HSAR017901	chr9:122382080-122382238:-	181
STRBP	chr9:124981289-124983008:-	5HSAR051120	chr9:124983075-124986329:-	455
DENND1A	chr9:125732006-125732090:-	5HSAR000413	chr9:125732118-125732210:-	233
LHX2	chr9:125814356-125814448:+	5HSAR003146	chr9:125814110-125814199:+	739
C9orf150	chr9:12765622-12765714:+	5HSAR000325	chr9:12765020-12765100:+<br>chr9:12765114-12765215:+	703
GARNL3	chr9:129066889-129066977:+	5HSAR014537	chr9:129066604-129066744:+<br>chr9:129066789-129066857:+	401
ST6GALNAC4	chr9:129714727-129716794:-	5HSAR017214	chr9:129718980-129719084:-	441

PTGES2	chr9:129929818-129929915:-	5HSAR031122	chr9:129929880-129930041:-	478
ODF2	chr9:130262322-130262658:+	5HSAR019709	chr9:130258283-130259401:+<br>chr9:130259496-130262274:+	585
PKN3	chr9:130504916-130505015:+	5HSAR015740	chr9:130504673-130504939:+	393
CCBL1	chr9:130647506-130684079:-	5HSAR024265	chr9:130683999-130684163:-	185
DOLK	chr9:130749404-130749506:-	5HSAR031645	chr9:130749558-130749779:-	430
FAM78A	chr9:133141388-133141478:-	5HSAR010546	chr9:133141519-133141653:-	340
NTNG2	chr9:134031966-134032039:+	5HSAR021651	chr9:134027174-134027272:+<br>chr9:134031665-134031739:+	776
SETX	chr9:134214637-134218951:-	5HSAR011886	chr9:134218927-134220162:-	183
BARHL1	chr9:134447899-134448005:+	5HSAR019955	chr9:134447826-134447933:+	192
TSC1	chr9:134794081-134800256:-	5HSAR031244	chr9:134800301-134809813:-<br>chr9:134794153-134800298:-	234
GTF3C5	chr9:134896127-134896219:+	5HSAR009524	chr9:134896040-134896105:+	337
RALGDS	chr9:135014189-135014286:-	5HSAR027733	chr9:135014295-135014378:-	221
C9orf7	chr9:135314924-135315009:+	5HSAR014494	chr9:135314908-135314979:+	102
NACC2	chr9:138082189-138126896:-	5HSAR014377	chr9:138082218-138126945:-	156
LCNL1	chr9:138997764-138997859:+	5HSAR022764	chr9:138997296-138997394:+	594
ABCA2	chr9:139042414-139042522:-	5HSAR010386	chr9:139042436-139042501:-	148
FUT7	chr9:139046265-139046363:-	5HSAR023791	chr9:139046977-139047084:-<br>chr9:139046463-139046933:-	849
SSNA1	chr9:139202903-139202999:+	5HSAR011407	chr9:139202875-139202997:+	125
LOC643596	chr9:139242515-139242608:+	5HSAR053435	chr9:139241972-139242450:+	480
NELF	chr9:139473376-139473476:-	5HSAR012905	chr9:139473405-139473551:-	232
PNPLA7	chr9:139564471-139564560:-	5HSAR019841	chr9:139564599-139564673:-	337
ZDHHC21	chr9:14664339-14670079:-	5HSAR059416	chr9:14670111-14683400:-	478

FREM1	chr9:14766252-14769515:-	5HSAR049904	chr9:14900907-14900975:-<br>chr9:14900841-14900906:-	89
TTC39B	chr9:15201383-15204212:-	5HSAR054750	chr9:15240060-15240170:-<br>chr9:15201390-15204205:-	407
CBWD1	chr9:168970-169071:-	5HSAR021842	chr9:167798-168969:-	106
CDKN2B	chr9:21998953-21999037:-	5HSAR029397	chr9:21999006-21999083:-	360
TOPORS	chr9:32542435-32542513:-	5HSAR052229	chr9:32542468-32542590:-	188
APTX	chr9:32979890-32987330:-	5HSAR023519	chr9:32987289-32991602:-	152
UBAP1	chr9:34193449-34210912:+	5HSAR048562	chr9:34169013-34193488:+	398
RUSC2	chr9:35529101-35536518:+	5HSAR014664	chr9:35528641-35528940:+<br>chr9:35528946-35536443:+	569
GBA2	chr9:35738702-35738776:-	5HSAR015350	chr9:35738782-35738955:-	524
TMEM8B	chr9:35831741-35832435:+	5HSAR019941	chr9:35824548-35824643:+	1015
GLIS3	chr9:4115865-4142090:-	5HSAR016209	chr9:4142108-4142176:-	194
LOC643395	chr9:45246630-45246736:-	5HSAR044964	chr9:45246812-45246958:-	341
C9orf68	chr9:4656251-4656520:-	5HSAR039168	chr9:4656507-4656653:-	246
LOC100132771	chr9:46684151-46684247:-	5HSAR044969	chr9:46686085-46686240:-<br>chr9:46685965-46686057:-	2203
LOC100132249	chr9:66233607-66233713:-	5HSAR044971	chr9:66233828-66233932:-	340
LOC728701	chr9:67899149-67899247:-	5HSAR044975	chr9:67900104-67900301:-<br>chr9:67900020-67900088:-	1187
TRPM6	chr9:76691985-76692071:-	5HSAR040931	chr9:76692755-76692820:-	99
PCSK5	chr9:77695821-77695917:+	5HSAR013711	chr9:77695384-77695773:+	538
GCNT1	chr9:78307032-78307117:+	5HSAR024737	chr9:78246477-78246542:+<br>chr9:78246549-78246644:+	939
GNA14	chr9:79452530-79452617:-	5HSAR020482	chr9:79452537-79452611:-	523
CEP78	chr9:80040994-80041086:+	5HSAR020562	chr9:80040839-80040913:+	276
TLE4	chr9:81377437-81377525:+	5HSAR011100	chr9:81376926-81377102:+<br>chr9:81377285-81377515:+	828

GKAP1	chr9:85611253-85621798:-	5HSAR01<br>5315	chr9:85611269-85621805:-	496
AGTPBP1	chr9:87517269-87546560:-	5HSAR00<br>9526	chr9:87517280-87546697:-	288
S1PR3	chr9:90805853-90805935:+	5HSAR01<br>8900	chr9:90796397-90805838:+<br>chr9:90805839-90805925:+	396
IARS	chr9:94091523-94095621:-	5HSAR01<br>5125	chr9:94095638-94095772:-	257
ASPN	chr9:94277001-94284401:-	5HSAR02<br>8991	chr9:94284414-94284596:-	300
IPPK	chr9:94472092-94472178:-	5HSAR01<br>6522	chr9:94472233-94472352:-	277
FBP1	chr9:96441414-96442213:-	5HSAR04<br>3745	chr9:96442213-96442329:-	230
LOC10013084<br>0	chr9:97308782-97308869:+	5HSAR00<br>6556	chr9:97308581-97308655:+	532
TRMT2B	chrX:100183935-100192964:-	5HSAR03<br>4119	chrX:100193545-100193712:-<br>chrX:100193002-100193365:-	529
CENPI	chrX:100241630-100242715:+	5HSAR01<br>7369	chrX:100241588-100241668:+	270
MORF4L2	chrX:102818612-102820145:-	5HSAR01<br>6745	[chrX:102820158-102826295:-	455
ZCCHC18	chrX:103245373-103245458:+	5HSAR02<br>2613	chrX:103244125-103244238:+<br>chrX:103244253-103244324:+	1316
MID1	chrX:10495588-10548365:-	5HSAR02<br>7010	chrX:10495590-10548380:-	401
RNF128	chrX:105823800-105823888:+	5HSAR01<br>7741	chrX:105823733-105823864:+	165
PRPS1	chrX:106758419-106758514:+	5HSAR02<br>3962	chrX:106758397-106758480:+	205
RGAG1	chrX:109575108-109580501:+	5HSAR01<br>9981	chrX:109548988-109575076:+	246
PAK3	chrX:110233125-110252987:+	5HSAR01<br>5445	chrX:110227480-110227566:+	621
DCX	chrX:110540859-110540947:-	5HSAR02<br>3064	chrX:110540916-110541020:-	172
KLHL13	chrX:116990820-116990909:-	5HSAR05<br>8204	chrX:116991655-116991720:-<br>chrX:116991559-116991654:-	910
FAM70A	chrX:119329193-119329295:-	5HSAR00<br>0743	chrX:119329269-119329418:-	227
LAMP2	chrX:119487053-119487142:-	5HSAR03<br>0345	chrX:119487139-119487207:-	180
FRMPD4	chrX:12066916-12067011:+	5HSAR01<br>6079	chrX:12066792-12066965:+	506

STAG2	chrX:122984064-122984158:+	5HSAR020003	chrX:122923273-122982944:+	313
SH2D1A	chrX:123308078-123308173:+	5HSAR057333	chrX:123307889-123307981:+	361
CCDC160	chrX:133198972-133206496:+	5HSAR013867	chrX:133198772-133198996:+	321
LOC100128949	chrX:133983288-133983377:+	5HSAR045740	chrX:133982213-133982290:+<br>chrX:133982334-133982402:+	1169
CXorf48	chrX:134132762-134132851:-	5HSAR018449	chrX:134133281-134133361:-<br>chrX:134133002-134133172:-	656
ZNF75D	chrX:134255733-134255812:-	5HSAR053129	chrX:134257496-134257567:-<br>chrX:134255738-134255809:-	390
MAP7D3	chrX:135166145-135166230:-	5HSAR049806	chrX:135166171-135166299:-	163
TCEANC	chrX:13583345-13587726:+	5HSAR020595	chrX:13581236-13583346:+	245
TRAPPC2	chrX:13648004-13662158:-	5HSAR056994	chrX:13662124-13662192:-	272
CSF2RA	chrX:1367502-1367707:+	5HSAR059908	chrX:1347762-1361631:+<br>chrX:1367426-1367494:+	362
CDR1	chrX:139694198-139694282:-	5HSAR000907	chrX:139694276-139694377:-	192
SLITRK2	chrX:144711322-144711635:+	5HSAR018458	chrX:144707190-144710573:+<br>chrX:144711041-144711617:+	990
FANCB	chrX:14793554-14796981:-	5HSAR026258	chrX:14796984-14801078:-	268
LOC100133153	chrX:148439304-148439398:-	5HSAR045746	chrX:148439809-148439949:-<br>chrX:148439645-148439722:-	691
HSFX1	chrX:148664055-148664147:+	5HSAR036237	chrX:148663560-148663655:+<br>chrX:148663666-148663938:+	614
HSFX2	chrX:148664055-148664147:+	5HSAR034260	chrX:148663560-148663655:+<br>chrX:148663666-148663938:+	614
CXorf40B	chrX:148852751-148852944:-	5HSAR000619	chrX:148857072-148857350:-	516
LOC100130935	chrX:151647479-151647581:-	5HSAR047039	chrX:151647534-151647614:-	153
CSAG1	chrX:151654541-151655158:+	5HSAR000712	chrX:151653928-151654598:+	328
CSAG2	chrX:151678440-151678542:+	5HSAR011747	chrX:151678395-151678487:+	153
PNCK	chrX:152592825-152592895:-	5HSAR029089	chrX:152592885-152592974:-	186
BCAP31	chrX:152642314-152642410:-	5HSAR017069	chrX:152642383-152642616:-	430

TKTL1	chrX:153177319-153177406:+	5HSAR024053	chrX:153177289-153177372:+	186
DKC1	chrX:153644340-153644434:+	5HSAR018993	chrX:153644340-153644434:+	210
F8	chrX:153904022-153904124:-	5HSAR029936	chrX:153904072-153904152:-	171
TMEM27	chrX:15592820-15592905:-	5HSAR003108	chrX:15592907-15592999:-	256
CTPS2	chrX:16630947-16640819:-	5HSAR018478	chrX:16640013-16640243:-<br>chrX:16639868-16640011:-	254
CDKL5	chrX:18435045-18435137:+]	5HSAR018193	chrX:18370440-18435034:+	249
PDHA1	chrX:19271979-19272076:+	5HSAR015169	chrX:19271937-19272002:+	145
CNKSR2	chrX:21302852-21302936:+	5HSAR053073	chrX:21302587-21302691:+	480
ZBED1	chrX:2418761-2428902:-	5HSAR058159	chrX:2418797-2428966:-	204
CXorf21	chrX:30488275-30488362:-	5HSAR031977	chrX:30504893-30505822:-<br>chrX:30488338-30504807:-	395
PRKX	chrX:3641295-3641387:-	5HSAR057502	chrX:3641392-3641616:-	381
SYTL5	chrX:37777987-37778086:+	5HSAR035729	chrX:37777868-37777963:+	356
OTC	chrX:38096805-38096893:+	5HSAR029594	chrX:38096707-38096772:+	214
MID1IP1	chrX:38549052-38549143:+	5HSAR013092	chrX:38548095-38548325:+<br>chrX:38548470-38548571:+	858
NYX	chrX:41192003-41192086:+	5HSAR016629	chrX:41191796-41191927:+	430
KDM6A	chrX:44617649-44617741:+	5HSAR029095	chrX:44617505-44617642:+	375
CDK16	chrX:46962908-46962995:+	5HSAR039022	chrX:46962563-46962715:+<br>chrX:46962770-46962856:+	524
ZNF41	chrX:47211825-47211904:-	5HSAR009908	chrX:47227042-47227149:-<br>chrX:47212051-47227019:-	640
CFP	chrX:47374188-47374268:-	5HSAR025734	chrX:47374509-47374589:-	126
SLC38A5	chrX:48211256-48212752:-	5HSAR011740	chrX:48212705-48213584:-	178
FTSJ1	chrX:48219712-48221379:+	5HSAR016446	chrX:48219510-48219650:+	323
WDR13	chrX:48342188-48342283:+	5HSAR034878	chrX:48341203-48341373:+<br>chrX:48341392-48341634:+	1116

PQBP1	chrX:48640653-48640736:+	5HSAR023068	chrX:48640542-48640616:+	254
TFE3	chrX:48787697-48787798:-	5HSAR023482	chrX:48787805-48787870:-	238
NUDT10	chrX:51091953-51092557:+	5HSAR021074	chrX:51091856-51091933:+	220
HUWE1	chrX:53697777-53723807:-	5HSAR019671	chrX:53723749-53729946:-	402
ALAS2	chrX:55070966-55074146:-	5HSAR028124	chrX:55074147-55074221:-	173
KLF8	chrX:56276411-56276492:+	5HSAR027827	chrX:56275802-56275888:+<br>chrX:56276378-56276458:+	486
SPIN3	chrX:57038106-57038472:-	5HSAR022646	chrX:57038545-57038664:-	342
SPIN2B	chrX:57163788-57164484:-	5HSAR001026	chrX:57164561-57164665:-	311
NLGN4X	chrX:6079508-6079600:-	5HSAR021686	chrX:6079658-6156603:-	430
ARHGEF9	chrX:62890918-62891004:-	5HSAR025842	chrX:62891588-62891743:-<br>chrX:62891392-62891463:-	839
FAM123B	chrX:63329892-63329977:-	5HSAR029400	chrX:63329964-63342349:-	273
HEPH	chrX:65300982-65307158:+	5HSAR058374	chrX:65300984-65307139:+	238
STARD8	chrX:67851862-67852941:+	5HSAR059470	chrX:67849500-67851942:+	348
GDPD2	chrX:69561969-69562365:+	5HSAR032242	chrX:69559653-69559856:+	484
FOXO4	chrX:70233012-70233103:+	5HSAR037931	chrX:70232763-70232933:+<br>chrX:70233002-70233073:+	380
NONO	chrX:70420207-70427212:+	5HSAR022165	chrX:70420006-70420089:+<br>chrX:70420100-70420258:+	539
FLJ44635	chrX:71281028-71281106:+	5HSAR001277	chrX:71280763-71280933:+<br>chrX:71280969-71281049:+	348
KIAA2022	chrX:73882211-74061483:-	5HSAR023999	chrX:74061444-74061620:-	617
HMGN5	chrX:80263705-80263804:-	5HSAR059901	chrX:80263826-80343988:-	329
POF1B	chrX:84521116-84521342:-	5HSAR019877	chrX:84521153-84521388:-	146
FAM133A	chrX:92850978-92851074:+	5HSAR058817	chrX:92815675-92815758:+<br>chrX:92815761-92815859:+	437
DIAPH2	chrX:95826614-95826713:+	5HSAR026476	chrX:95826363-95826698:+	396

PCDH19	chrX:99550252-99550347:-	5HSAR026444	chrX:99551711-99551821:-<br>chrX:99551409-99551507:-	1676
UTY	chrY:14100940-14101023:-	5HSAR011492	chrY:14101782-14101916:-<br>chrY:14101655-14101720:-	1005
HSFY1	chrY:19167974-19168061:+	5HSAR025973	chrY:19167974-19168060:+	97
HSFY2	chrY:19394893-19394980:-	5HSAR003245	chrY:19394894-19394980:-	97
CYorf15B	chrY:20218816-20218906:+	5HSAR018441	chrY:20217897-20217998:+<br>chrY:20218002-20218082:+	1077
C20orf135	Hchr20:61963246-61963337:+	5HSAR000582	chr20:61963077-61963193:+<br>chr20:61963201-61963326:+	328
ZRANB2	Homo sapiens	5HSAR019346	chr1:71319267-71319296:-	294
GTF2H1	Homo sapiens	5HSAR023414	chr11:18300517-18300792:+<br>chr11:18304212-18311192:+	487
NR3C1	Homo sapiens	5HSAR030192	chr5:142795092-142795265:-<br>chr5:142794905-142795024:-	492
SERPINB6	Homo sapiens	5HSAR019582	chr6:2904566-2916963:-	643
TNFRSF10C	Homo sapiens	5HSAR024994	chr8:23016385-23016459:+	308
RAB9A	Homo sapiens	5HSAR021955	chrX:13617269-13636786:+	283
SLC37A4	hr11:118404276-118404672:-	5HSAR053441	chr11:118404280-118405450:-	402
SCNN1A	hr12:6356694-6356776:-	5HSAR029881	chr12:6354236-6355010:-	91
CEP63	hr3:135687674-135696852:+	5HSAR025302	chr3:135687279-135687374:+	173

* Data source is from UTRdb

5..109

112..186

26..117

No gene
founded.

No gene
founded.
No gene
founded.
No gene
founded.
