# Supplementary material for: Overexpression of FGF9 in colon cancer cells is mediated by hypoxia-induced translational activation
Source: Nucleic Acids Res. 2013 Dec 10;42(5):2932–44. doi: 10.1093/nar/gkt1286 (PMC3950685; doi:10.1093/nar/gkt1286)
Supplement: Supplementary Data [file supp_gkt1286_nar-02603-x-2013-File009.doc]

**SUPPLEMENTARY DATA**

Contains three tables and five figures.

**SUPPLEMENTARY Tables**

**Supplementary Table S1.** **Primers used in this study**

| Name | Sequence from 5′ to 3′ | Description |
| --- | --- | --- |
| FGF9_FL-F | GCATCCATGGTGTGCCAGTGAAACAGCAGA | For amplification of full length FGF9 5’UTR (nt.1-178) |
| FGF9_FL -R | GTAACCATGGCAGAGGACTCGGCTTTTGGA |
| FGF9_IRES-F | AACCTGTGCCAGGTAAGGGAGGGGA | For amplification of IRES element (nt.84-178) |
| FGF9_IRES-R | TCCCCTCCCTTACCTGGCACAGGTT |
| FGF9_ΔIRES-R | ATCCTCGAGATGGACAGCAAGATGAAGAAGATGGCAGAG | FGF9 5’UTR-F andΔIRES-R for deletion of IRES element (nt. 1-83) |
| Slot 1 | AATATCCATTAAATGCATAAATAAAAGTAATCTGCTGTTTCA | For slot blotting |
| Slot 2 | GGTTCTTTTCTTCAATCCATTAAATGCATAAATATCCATTA | For slot blotting |
| Slot 3 | GCAGAGAGAGAGAGAAAAAAAAGGTTCTTTTCTTC | For slot blotting |
| Slot 4 | CCCCTCCCTTACTGCAGTTGCAGAG | For slot blotting |
| Slot 5 | TAGGATATTAGGCGAGGTATATCCAACTCCC | For slot blotting |
| Slot 6 | CACAATAATGATGGTGTCAACCCAGGAGATATTAG | For slot blotting |
| Slot 7 | CTCGGGCTTTTGGAGCACAAGAATAAACACAAT | For slot blotting |
| Slot 8 | CCTAAGGGAGCCATCAGAGGACTCG | For slot blotting |
| Slot 9 | GCACACCGAAATAGTTCCCAACTTCACCTAA | For slot blotting |
| Slot 10 | CACATTCCCAAACGGTACCGCATCCT | For slot blotting |
| β-actin 5’UTR | CGATATCATCATCCATGGTGAGCTGGCG | Positive control for slot blotting |
| FGF9 3’UTR | CGTGAAACCTTTATAGTGGTTACTTTTTTAAGGGCTCAA | Negative control for slot blotting |

**Supplementary Table S2**: Clinical parameters of patients

| **ID** | **Sex^1^** | **Age** | **Differentiation^2^** | **Tumor status** | **Nodal status** | **AJCC stage** |
| --- | --- | --- | --- | --- | --- | --- |
| 27 | M | 75 | M | T3 | N2 | IIIC |
| 29 | M | 64 | M | T2 | N1 | IIIA |
| 37 | F | 47 | p | T4 | N2 | IV |
| 39 | F | 40 | M | T3 | N0 | IIB |
| 40 | M | 45 | P | T4 | N1 | IV |
| 41 | M | 65 | P | T3 | N1 | IIIB |
| 42 | M | 75 | M | T3 | N0 | IIA |
| 43 | F | 62 | W | T1 | N0 | I |
| 44 | F | 82 | M | T3 | N1 | IIIB |
| 45 | F | 57 | W | T3 | N2 | IV |
| 47 | M | 59 | P | T3 | N1 | IIIB |
| 48 | F | 71 | M | T3 | N0 | IIA |
| 50 | F | 66 | M | T3 | N2 | IIIC |
| 52 | M | 84 | M | T3 | N0 | IIA |
| 53 | M | 47 | M | T3 | N1 | IIIB |
| 54 | M | 67 | M | T3 | N0 | IIB |
| 56 | M | 62 | M | T2 | N0 | I |
| 57 | F | 48 | M | T3 | N2 | IIIC |
| 59 | M | 58 | M | T2 | N0 | I |
| 60 | F | 86 | M | T4 | N2 | IV |
| 62 | F | 58 | M | T3 | N1 | IIIB |
| 65 | F | 75 | M | T4 | N2 | IIIC |
| 66 | F | 68 | M | T3 | N0 | IIA |
| 67 | M | 35 | M | T3 | N0 | IIB |
| 70 | M | 39 | P | T4 | N0 | IV |
| 78 | M | 28 | M | T4 | N1 | IV |
| 94 | M | 73 | M | T3 | N2 | IV |
| 101 | F | 82 | M | T3 | N1 | IV |
| 107 | M | 69 | M | T3 | N1 | IV |
| 111 | M | 91 | M | T3 | N0 | IIA |
| 126 | M | 68 | W | T3 | N2 | IV |
| 130 | M | 73 | M | T1 | N0 | I |
| 132 | F | 60 | W | T1 | N0 | I |
| 134 | M | 66 | W | T1 | N0 | I |
| 140 | F | 53 | W | T1 | N0 | I |
| 151 | M | 56 | M | T4 | N2 | IV |
| 179 | M | 60 | M | T1 | N0 | I |
| 214 | M | 71 | W | T1 | N0 | I |
| 279 | M | 60 | M | T0 | N0 | Ⅰ |
| 298 | M | 65 | W | T1 | N0 | Ⅰ |
| 12^3^ | F | 56 | W | NA^4^ | N1 | IIC |
| 15^3^ | F | 72 | M | NA | N2 | IV |
| 17^3^ | F | 55 | W | T4 | N0 | IV |
| 18^3^ | M | 72 | M | T4 | N0 | IIB |
| 20^3^ | M | 65 | M | T3 | N0 | IIB |
| 21^3^ | F | 78 | M | T3 | N0 | I |
| 24^3^ | F | 56 | M | T3 | N2 | IIIC |
| 27^3^ | M | 63 | M | T3 | N0 | IV |
| 28^3^ | M | 60 | M | T2 | N0 | IB |
| 30^3^ | M | 57 | M | T4 | N2 | IIIC |
| 31^3^ | F | 50 | M | NA | NA | IV |
| 39^3^ | F | 67 | W | T2 | N0 | IB |
| 41^3^ | F | 47 | M | T3 | N1 | IVB |
| 44^3^ | M | 64 | M | T2 | N0 | IB |
| 1: M: male; F: female | | | | | | |
| 2: W: well differentiated; M: moderate differentiated; P: poorly differentiated | | | | | | |
| 3: additional colon cancer samples from the Human Biobank, Research Center of Clinical Medicine, National Cheng Kung University Hospital. | | | | | | |
| 4: NA: not available | | | | | | |

**Supplementary Table S3.** The association of IRES-containing transcripts with uORF **(In a separated Excel document)**

**SUPPLEMENTAL FIGURE LEGEND**

**Supplementary Figure S1. Hypoxia has no effect on *FGF9* 5’UTR uORF-mediated translation.** Constructs with *FGF9* full-length 5’UTR (Wt) or mutant uROF (mATGI, mATGII and dmATG) were transfected into HEK293 cells under normoxia (□) or hypoxia (■) conditions. Luciferase activities showed no detectable differences in hypoxia and normoxia. Thus it implies the FGF9-uORF is not response to hypoxia.

**Supplementary Figure S2. Hypoxia enhances the translational efficiency of *FGF9* mRNA.** RNA was isolated form fractionated cellular fraction of HEK293 cells cultured in normoxic or hypoxic condition. Polysome profiles of *FGF9* and *β-actin* mRNAs were established. Relative mRNA level of FGF9 were quantified by RT-qPCR analysis and showed as ratio to total FGF9 mRNA amount (first panel). *GAPDH* mRNA was used as a control for hypoxia treatment that represses house-keeping gene expression (Thomas and Johannes, 2007); second panel); *β-actin* mRNA was used as an internal control (third panel). Total RNAs from each fraction was shown (fourth panel).

**Supplementary Figure S3. Change in translation initiation site of *FGF9* mRNA demonstrated a hypoxia-mediated RNA switch.** S6-IP pulled down RNAs were hybridized to various probes on FGF9 5’UTR locations. Results from 3 independent experiments and slot blots are shown. The “+“ and “-“ are signals from the probes of β-actin 5’UTR and FGF9 3’UTR, respectively, which were used as the positive and negative controls in this study. The results presented in the bottom panel were from experiments using undigested RNA.

**Supplementary Figure S4. Secondary structure of FGF9 5’UTR are changed in different free energy condition.** Results of the minimum free-energy algorithm showed that secondary structures were formed in FGF9 5'UTR at lower free-energy conditions (A, right), including one internal loop (I-loop), one multibranch loop (M-loop), and two hairpin loops (H-loop). **B.** FGF9 IRES region showed a conserved sharp Y secondary structure not influenced by the energy change.

**Supplementary Figure S5. hnRNPA1 are not binding to FGF9 IRES.** To analyze proteins bound to FGF9 IRES element, plasmids containing full-length or partial 5’UTRs were linearized by BamH1 restriction enzymes and used as template for biotinylated probe synthesis *in vitro* (Roche). 100 ug of cytoplasmic extract was incubated with 1 ug of biotinylated RNA for 1 hour at room temperature to pull-down proteins that bind to biotinylated probes, and followed by Western blot analysis. hnRNPA1 is the known ITAF for FGF2 and a quick test revealed no binding activity on FGF9 IRES. In contrary, the well-known RNA-binding protein HuR binds to FGF9 at non-IRES region of 5’UTR. No template control (NTC) was used as a negative control for pull-down assay; input was used as a positive control for Western blotting.

**Supplementary Figure S1**

**
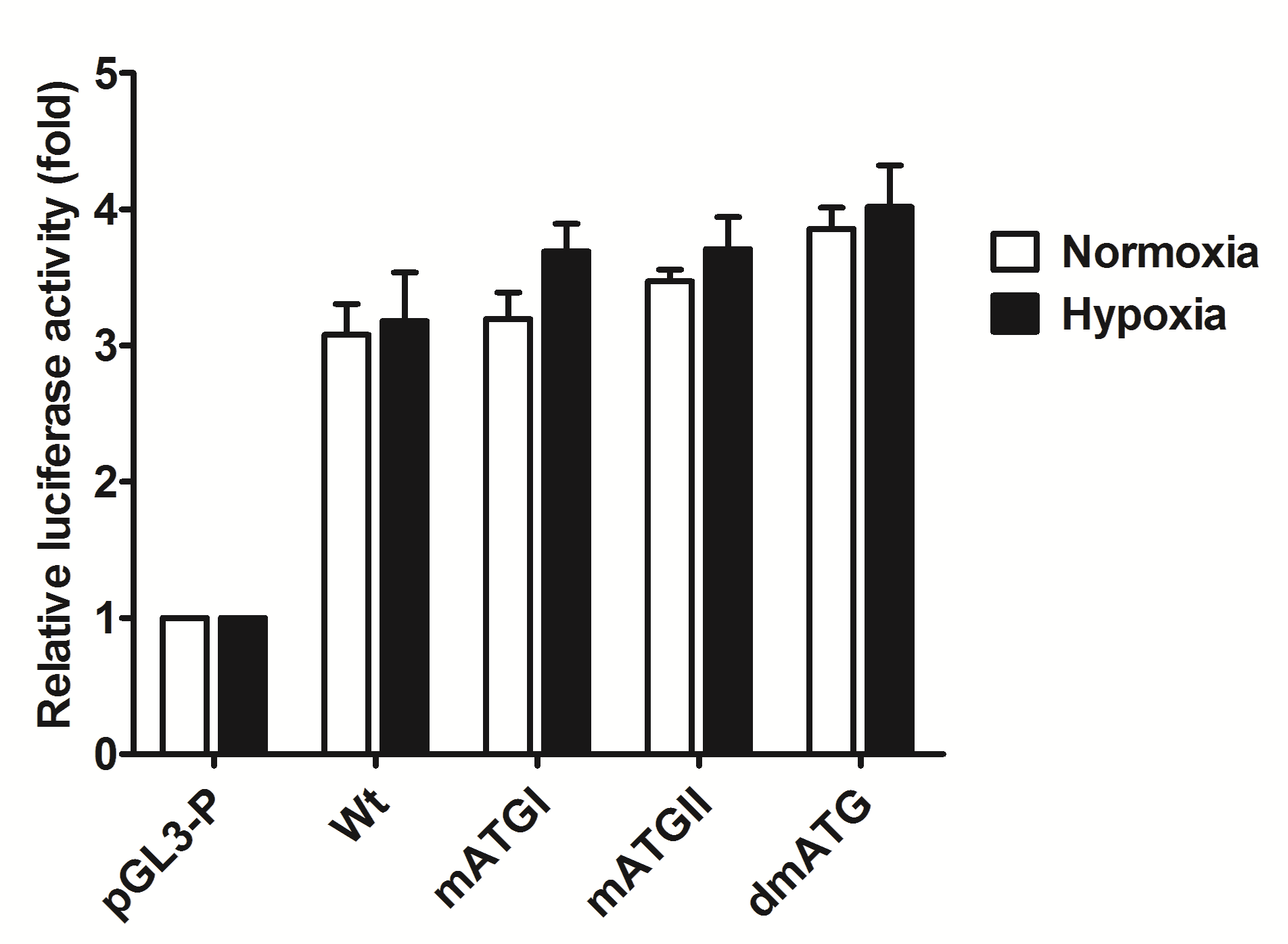
**

**Supplementary Figure S2**

**
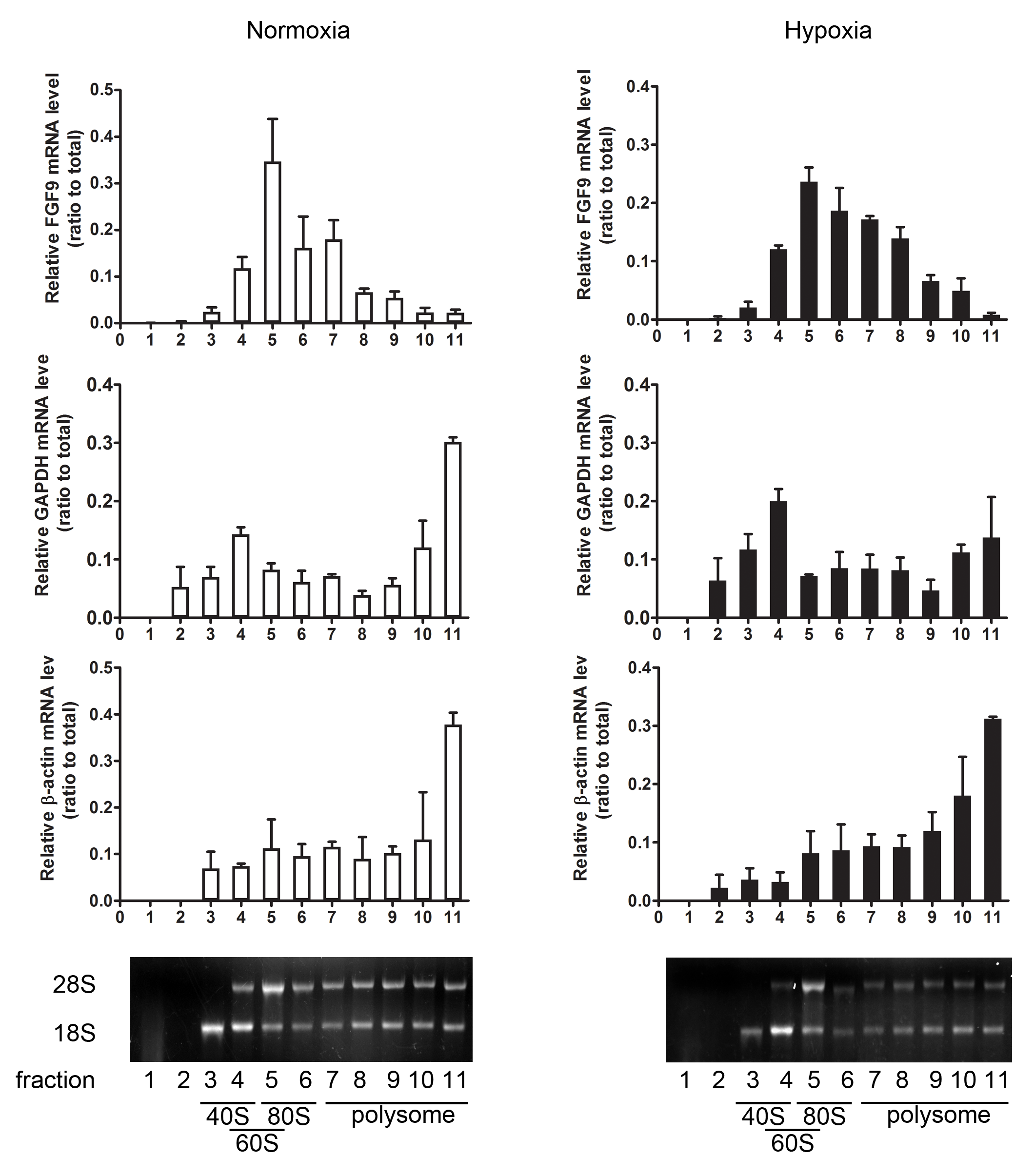
**

**Supplementary Figure S3**

**
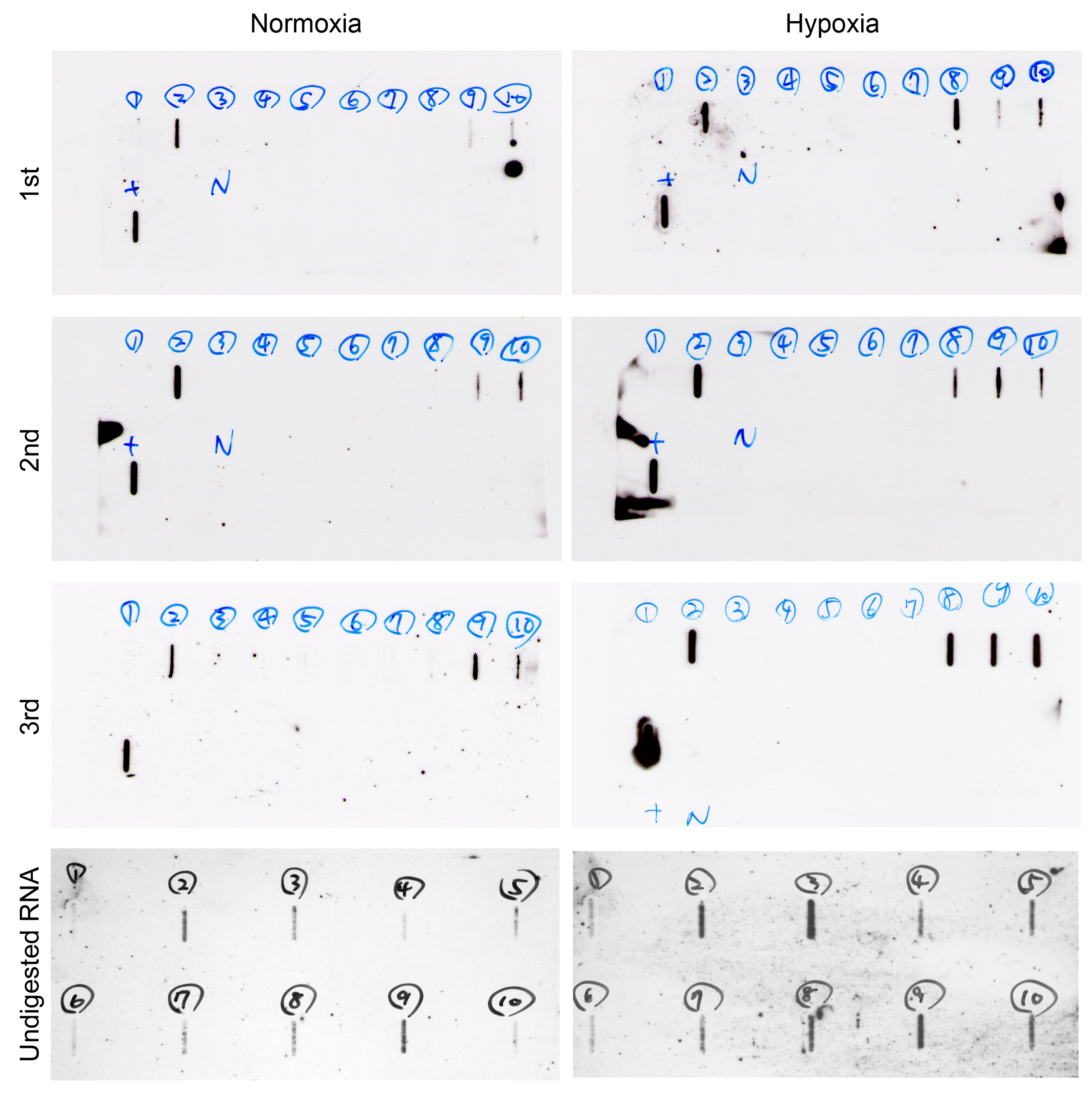
**

**Supplementary Figure S4**

**
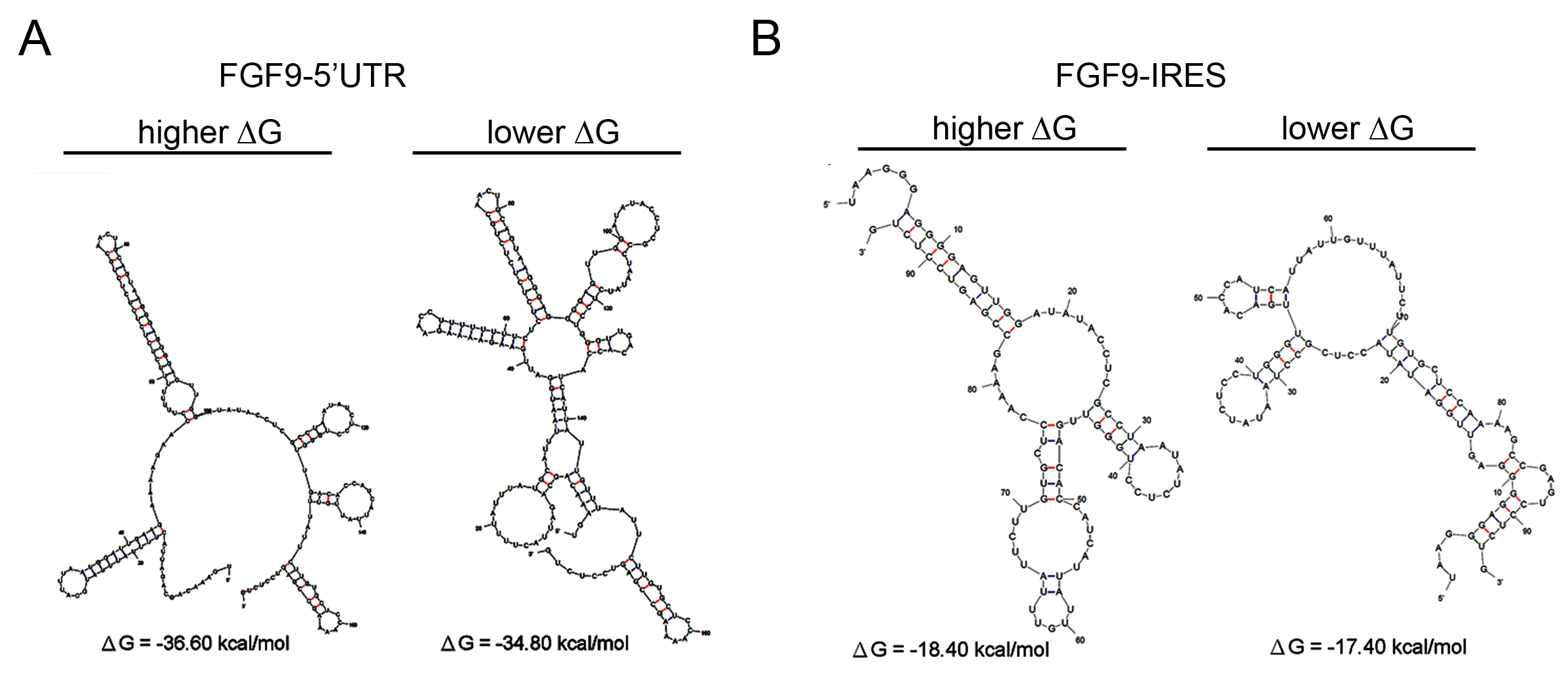
**

**Supplementary Figure S5**

**
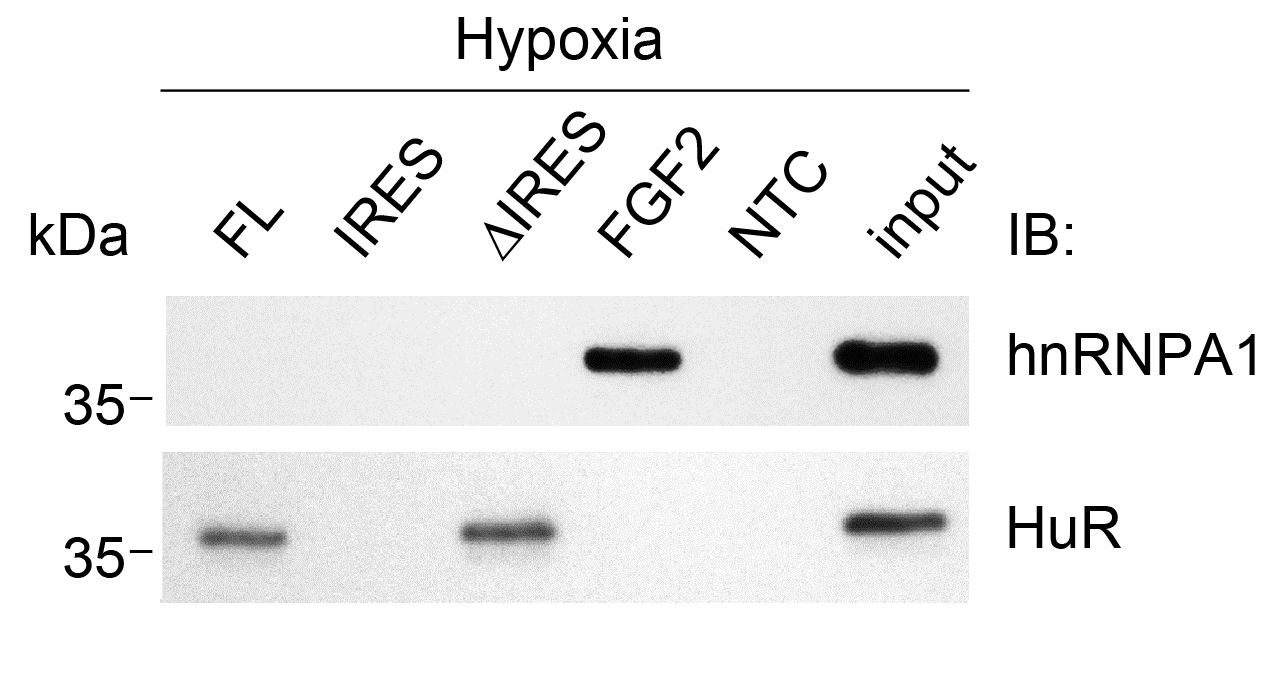
**

**Reference.**

Thomas, J.D. and Johannes, G.J. (2007) Identification of mRNAs that continue to associate with polysomes during hypoxia. Rna 13(7), 1116-31.
